# The Cerebellar Connectome Disruptions in Ischemic Stroke

**DOI:** 10.1002/cns.70759

**Published:** 2026-01-23

**Authors:** Xiuqin Wang, Tongyue Li, Jinhui Wang, Yanhui Fu, Zhenqiang Ma, Xiaoyan Wu, Yiying Wang, Yufeng Zang, Yulin Song, Yating Lv

**Affiliations:** ^1^ College of Life and Environmental Sciences Hangzhou Normal University Hangzhou China; ^2^ Department of Neurology The Affiliated Hospital of Hangzhou Normal University Hangzhou China; ^3^ Institute of Psychological Science Hangzhou Normal University Hangzhou China; ^4^ Zhejiang Key Laboratory for Research in Assessment of Cognitive Impairments Hangzhou China; ^5^ Institute for Brain Research and Rehabilitation South China Normal University Guangzhou China; ^6^ Department of Neurology Anshan Changda Hospital Anshan China; ^7^ Department of Image Anshan Changda Hospital Anshan China; ^8^ Department of Ultrasonics Anshan Changda Hospital Anshan China

**Keywords:** cerebellum, functional connectivity, ischemic stroke

## Abstract

**Background:**

Supratentorial focal lesions following ischemic stroke can lead to crossed cerebellar diaschisis (CCD). However, it remains unclear how CCD affects the functional connectivity between the cerebellum and the rest of the brain in ischemic stroke patients.

**Methods:**

This case–control study involved resting‐state fMRI data from 65 patients with basal ganglia ischemic stroke (Stroke) and 72 healthy controls (HC). Cerebral, cerebellar, and cerebrocerebellar inter‐module functional connectivity in both 7‐module and 17‐module conditions were calculated and compared between the Stroke and HC groups. Spearman correlation analyses were further conducted to examine the relationships between connectivity alterations and both stroke severity and lesion size in Stroke patients.

**Results:**

The Stroke patients exhibited disrupted inter‐module functional connectivity, characterized by increased intra‐hemispheric and decreased inter‐hemispheric connectivity between cerebral modules, increased inter‐module connectivity in the cerebellum, and reduced connectivity between ipsilesional cerebral modules and cerebellar modules while increasing connectivity between contralesional cerebral modules and cerebellar modules. Moreover, these connectivity changes, particularly disruptions in the cerebellar connectome, may be associated with lesion size and stroke severity in Stroke patients.

**Conclusions:**

These findings highlight the importance of cerebellar connectome disruptions in ischemic stroke, which may provide valuable insights into the disease's underlying brain mechanisms.

## Graphical abstract

Ischemic stroke disrupts the brain's modular organization (based on the Yeo/Buckner template). In patients with basal ganglia ischemic stroke, inter‐module functional connectivity is altered, characterized by increased intra‐cerebellar connectivity, reduced connectivity between ipsilesional cerebral and cerebellar modules, and enhanced connectivity between contralesional cerebral and cerebellar modules. Moreover, these cerebellar connectivity changes showed preliminary correlations with lesion size and clinical severity in exploratory analyses. These distinct network disruptions provide novel insights into post‐stroke deficits and potential targets for neuromodulation therapies.

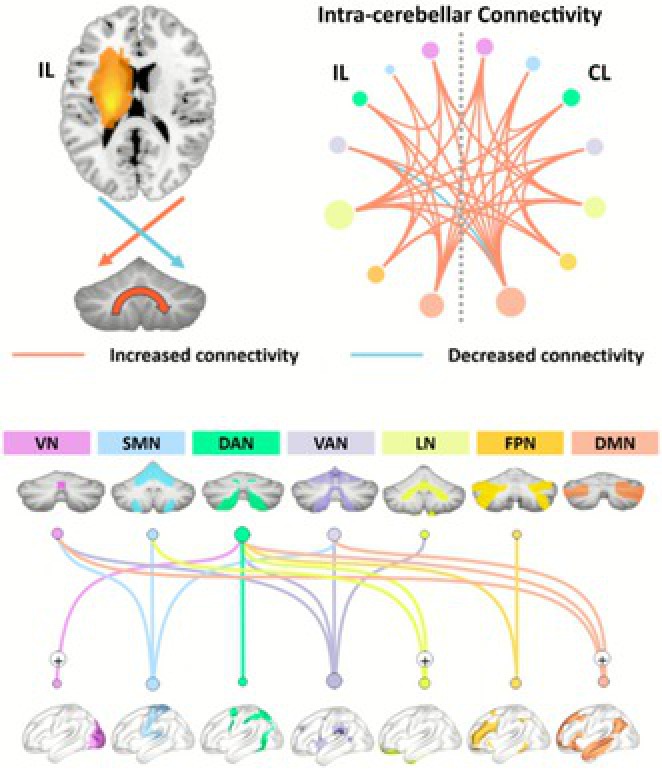

## 1 Introduction

Ischemic stroke is a major type of stroke caused by the obstruction or narrowing of arteries supplying blood to the brain, leading to reduced blood flow and oxygen supply to the affected brain tissues. Focal lesions after ischemic stroke onset could cause widespread dysfunctions in brain areas distant from the infarction, even in the contralateral hemisphere [1, 2]. Growing evidence suggests that the connectivity‐based network approaches are more appropriate to investigate how the brain responds to a focal lesion after ischemic stroke [1, 2, 3, 4]. Functional neuroimaging studies have consistently identified the alterations within and between different cerebral networks, including somatomotor network [5, 6, 7, 8], default mode network [9, 10, 11], dorsal attention network [12, 13], decreases in the modularity of intra‐hemispheric network organization [13, 14, 15], and reduced inter‐hemispheric functional connectivity particularly between homotopic areas in both hemispheres [13, 15, 16]. However, these studies have predominantly focused on the connectivity changes in cerebral networks, with less attention given to the cerebellum.

The cerebellum has traditionally been viewed as responsible for motor control. However, it has been reported that the cerebellum has a complex internal structure and close functional connections to both motor and non‐motor areas of the cerebral cortex, particularly those related to high‐level cognitive processes [17, 18]. Buckner and colleagues further demonstrated that the cerebellum exhibits a modular organization (i.e., distinct functional networks) similar to the cerebrum, as revealed by cerebrocerebellar functional connectivity analyses [19, 20]. Following ischemic stroke, the supratentorial lesions can lead to crossed cerebellar diaschisis (CCD), where cerebellar blood flow and metabolic activity decrease contralateral to the lesions [21]. CCD reflects functional inhibition between the cerebrum and the contralateral cerebellum resulting from both structural disconnection of cortico‐ponto‐cerebellar pathways and metabolic deactivation of cerebellar Purkinje cells [22, 23]. Studies have shown decreased connectivity between the ipsilesional cerebral hemisphere and contralateral cerebellum, as well as increased connectivity between the contralesional cerebral hemisphere and ipsilateral cerebellum, particularly within the somatomotor network [24, 25]. In post‐stroke recovery, both the preserved structural integrity of cerebellar outflow pathways and the adaptive reorganization of cortex‐cerebellar functional connectivity were significantly associated with motor function recovery [24, 26]. These findings highlight the cerebellar connectome's potential as a biomarker for predicting recovery risk and personalizing rehabilitation. However, it remains unclear whether CCD affects the connectivity between nonmotor cerebrocerebellar networks or inter‐ and intra‐hemispheric cerebellar functional networks. Additionally, infarcts in the basal ganglia are more likely to cause CCD [27, 28], with the lesion size and ischemic stroke severity potentially influencing connectivity changes [27]. Thus, the impact of lesion size and stroke severity on connectivity alterations in ischemic stroke patients warrants further investigation.

This study employs resting‐state functional magnetic resonance imaging (rs‐fMRI) to investigate cerebral, cerebellar, and cerebrocerebellar connectivity disruptions within and between different functional networks/modules in patients with basal ganglia ischemic stroke, and to further investigate how these connectivity impairments are associated with lesion size or disease severity in ischemic stroke patients. We hypothesize that basal ganglia ischemic stroke leads to cerebellar connectome disruptions, including: (1) impaired intra‐ and inter‐hemispheric connectivity between cerebellar modules; (2) abnormal inter‐module cerebrocerebellar connectivity extending beyond the ipsilesional cortical‐cerebellar circuit, with significant compromise in contralesional pathways. Furthermore, we hypothesize that these connectivity alterations correlate with lesion size and clinical severity in stroke patients. By testing these hypotheses, this study aims to provide initial evidence regarding the impact of cerebellar connectivity changes on post‐stroke impairment and to lay the groundwork for exploring cerebellar‐targeted prognostic and rehabilitation strategies.

## 2 Materials and Methods

### 2.1 Participants

A total of 73 patients with first‐time ischemic stroke (Stroke) were recruited from the Department of Neurology, Anshan Changda Hospital. Each patient met the following inclusion criteria: (1) diagnosis of ischemic stroke by neurologists; (2) admitted < 1 month after stroke onset; (3) unilateral focal brain lesions; (4) infarction in basal ganglia areas; (5) no significant cognitive impairment (Mini‐Mental State Examination (MMSE) scores > 24) [29]. Exclusion criteria included: (1) history of psychiatric or neurological disorders (e.g., dementia, Parkinson's disease, traumatic brain injury); (2) intracranial hemorrhage, epilepsy, or migraine; (3) contraindications to MRI; (4) other systemic diseases affecting cognitive functions (e.g., severe renal/hepatic failure). Additionally, to avoid the potential confounding effects of acute interventions on functional connectivity, patients who had undergone reperfusion therapies (i.e., thrombolysis or thrombectomy) were also excluded.

In addition, 74 age‐ and gender‐matched healthy controls (HC) were recruited from the local community. None of the healthy controls had a history of physical or psychiatric diseases.

Data from 8 stroke patients and 2 healthy controls were excluded due to excessive head motion and poor data quality, including incomplete scanning of the cerebellum and inconsistent scanning parameters (see Preprocessing of rs‐fMRI data). Leaving 65 patients in the Stroke group (27 females, 58.08 ± 8.19 years) and 72 healthy controls in the HC group (30 females, 57.44 ± 6.92 years) for the final analyses.

### 2.2 Data Acquisition

#### 2.2.1 Clinical Data Collection

To evaluate stroke severity, each patient underwent the National Institutes of Health Stroke Scale (NIHSS) assessment within 24 h before the MRI scan [30, 31].

#### 2.2.2 MRI Data Acquisition

Rs‐fMRI, structural MRI (sMRI), and diffusion‐weighted imaging (DWI) data were collected from all participants using a 3T scanner (GE MR‐750, Waukesha, WI) at Anshan Changda Hospital. Please refer to the Supporting Information for parameters of MRI data acquisition.

After careful visual inspection, 8 stroke patients and 2 healthy controls were excluded from further analysis due to excessive head motion (3), incomplete coverage of the cerebellum in the scan (5), and inconsistent scanning parameters (2).

### 2.3 Data Preprocessing

#### 2.3.1 Processing of sMRI Image and Lesion Map

According to the high‐resolution sMRI and DWI images for each patient, one experienced radiologist (F.C.) manually traced lesion masks using ITK‐SNAP software (https://www.itksnap.org). The lesion masks were smoothed with a 3 mm full‐width half maximum to remove jagged edges [32]. Subsequently, the lesion masks, DWI, and sMRI images from patients with a right‐hemispheric lesion were flipped to the left hemisphere [33, 34]. Based on the masks, the superior longitudinal fasciculus algorithm was used to refill the lesions on individual sMRI images (http://act.udg.edu/salem/slfToolbox/ software.html).

The corrected sMRI images were then segmented and normalized to the Montreal Neurological Institute (MNI) space using the prior templates provided in SPM to obtain deformation information. Two lesion masks from sMRI and DWI images were further normalized to the MNI space via deformation fields derived from individual sMRI tissue segmentation without applying any lesion‐specific template. Finally, the union of two masks was calculated to obtain the lesion map for each patient. Overlapping lesions for the Stroke patients were displayed in Figure S1.

#### 2.3.2 Preprocessing of rs‐fMRI Data

The rs‐fMRI data were preprocessed using the GRETNA package [35] based on SPM12 (http://www.fil.ion.ucl.ac.uk/spm) in the MATLAB platform. The preprocessing steps included: (1) right‐hemisphere lesions were flipped to standardize ipsilesional hemispheres as left [33, 34]; (2) removal of initial 5 volumes; (3) slice timing correction; (4) head motion correction (exclusion criteria: > 3 mm displacement, > 3°rotation or > 0.5 mean framewise displacement) [36]. We did not perform scrubbing to preserve temporal continuity and degrees of freedom [37]; (5) Nuisance regression (24 motion parameters [38], global signal, white matter and cerebrospinal fluid signals thresholded individual mask at 0.9); (6) band‐pass filtering (0.01–0.08 Hz); (7) spatial normalization to MNI space via deformation fields derived from tissue segmentation of sMRI images; (8) hemodynamic lag correction shifted the time series of each voxel according to their hemodynamic lags as calculated by the time‐shift analysis approach [39] (see details in Supporting Information), addressing known connectivity artifacts in ischemic stroke [40, 41].

#### 2.3.3 Cerebral and Cerebellar Module Definition

This study utilized two well‐established parcellation schemes: (1) the 7‐network and 17‐network cortical functional parcellations proposed by Yeo et al. (2011) [19], and (2) the cerebellar functional parcellation developed by Buckner et al. (2011) [20]. Given their widespread recognition and extensive application in neuroimaging research, these schemes were selected to investigate cerebrocerebellar connectivity alterations.

Both the cerebrum and cerebellum were separately parcellated into 7 and 17 modules based on intrinsic functional connectivity patterns: (1) 7 functional modules: visual network (VN), somatomotor network (SMN), dorsal attention network (DAN), ventral attention network (VAN), limbic network (LN), frontoparietal network (FPN), and default mode network (DMN); (2) 17 functional modules: VN A, VN B, SMN A, SMN B, DAN A, DAN B, VAN A, VAN B, LN A, LN B, FPN C, FPN A, FPN B, temporal parietal network (TPN), DMN C, DMN A, and DMN B [19, 20]. Due to few voxels, the first network (VN A) of 17 functional modules in the cerebellum was excluded. Subsequently, each module was further divided into two hemispheres, resulting in 14 and 34 modules in the cerebrum, and 14 and 32 modules in the cerebellum, respectively.

#### 2.3.4 Module‐Level Cerebral and Cerebellar Connectivity Estimation

The time series of each module were first calculated as the average BOLD signal over all voxels (removed those within the lesions) within the module. Pearson correlation coefficients between the time series of each module pair were then calculated to estimate inter‐module functional connectivity. For each participant, we constructed two module‐level correlation matrices: one 28 × 28 (for 7 modules) and one 66 × 66 (for 17 modules) matrix. These correlation matrices were further converted to z‐values using Fisher's r‐to‐z transformation.

### 2.4 Statistical Analysis

#### 2.4.1 Group Difference in Demographic and Clinical Characteristics Analysis

All statistical analyses for demographic and clinical data were carried out online using SPSS (https://www.spsspro.com/). For continuous variables, normality was assessed using the Shapiro–Wilk test, followed by a two‐sample *t*‐test for group comparison (e.g., age). For categorical variables, intergroup differences were evaluated using the chi‐square test (e.g., gender).

#### 2.4.2 Group Difference in Module‐Level Functional Connectivity

A nonparametric permutation test was applied to compare inter‐module functional connectivity differences between the Stroke and HC groups, in which gender and age as covariates. For each module pair, we initially calculated the between‐group differences of the mean values. An empirical distribution of the differences was then obtained by randomly reallocating values into two groups and recomputing the mean differences between the two randomized groups (10,000 permutations). A false discovery rate (FDR) procedure (*p* < 0.01) was applied to control the false positive rate in multiple comparisons, with this stringent threshold selected to enhance result reliability.

To consolidate our findings, we also reanalyzed between‐group differences in inter‐module functional connectivity by controlling for age, gender, MMSE, and educational level simultaneously. Separately, to assess the possible effects of lesion laterality, all patients were divided into a left‐hemisphere lesion group (*n* = 36) and a right‐hemisphere lesion group (*n* = 29). The differences in inter‐module functional connectivity were then compared separately between each subgroup and the HC group.

#### 2.4.3 Relationship Between Connectivity Metrics and Clinical Variables in Stroke

For any inter‐module functional connectivity exhibiting significant between‐group differences, exploratory Spearman correlation analysis was then performed to assess its potential associations with NIHSS score and lesion size, with statistical significance set at *p* < 0.01.

To further examine the robustness of the correlation results, we performed Spearman correlation analyses by treating age, sex, MMSE, education, and days since onset as nuisance covariates.

## 3 Results

### 3.1 Group Differences in Demographics and Clinical Characteristics

There were no significant differences in age and gender (*p* > 0.05) between the Stroke and HC groups. Demographic and detailed clinical information are presented in Table 1.

**TABLE 1:** Demographic and clinical characteristics of participants.

	Stroke (*n* = 65)	HC (*n* = 72)	*p*
Gender (M/F)	38/27	42/30	0.988*
Age (years)	58.08 ± 8.19	57.44 ± 6.92	0.625^†^
MMSE score	29.50 ± 1.17	29.40 ± 1.34	0.628^†^
Vascular risk factors, *n* (%)			
Hypertension	54 (83.08%)		
Diabetes mellitus	21 (32.31%)		
Onset time (days)^a^	6.62 ± 4.74		
Lesion hemisphere (L/R)	36/29		
Lesion size (voxels)	16–1459		
NIHSS score	3.23 ± 2.80		

Abbreviations: F, female; HC, healthy controls; L, left; M, male; MMSE, Mini‐Mental State Examination; NIHSS, National Institutes of Health Stroke Scale; R, right; stroke, ischemic stroke patients.

* The *p*‐value was obtained by a chi‐square test.

^†^ The *p*‐values were obtained by a two‐sample *t*‐test.

^a^ Onset time (days) refers to the time between stroke onset and MRI scanning.

### 3.2 Group Differences in Module‐Level Functional Connectivity

Nonparametric permutation test results revealed significant between‐group differences in module‐level functional connectivity in the Stroke group, delineated across cerebral, cerebellar, and cerebrocerebellar patterns (*p* < 0.01, FDR corrected).

#### 3.2.1 Alterations in Cerebral Inter‐Module Functional Connectivity

**FIGURE 1:**
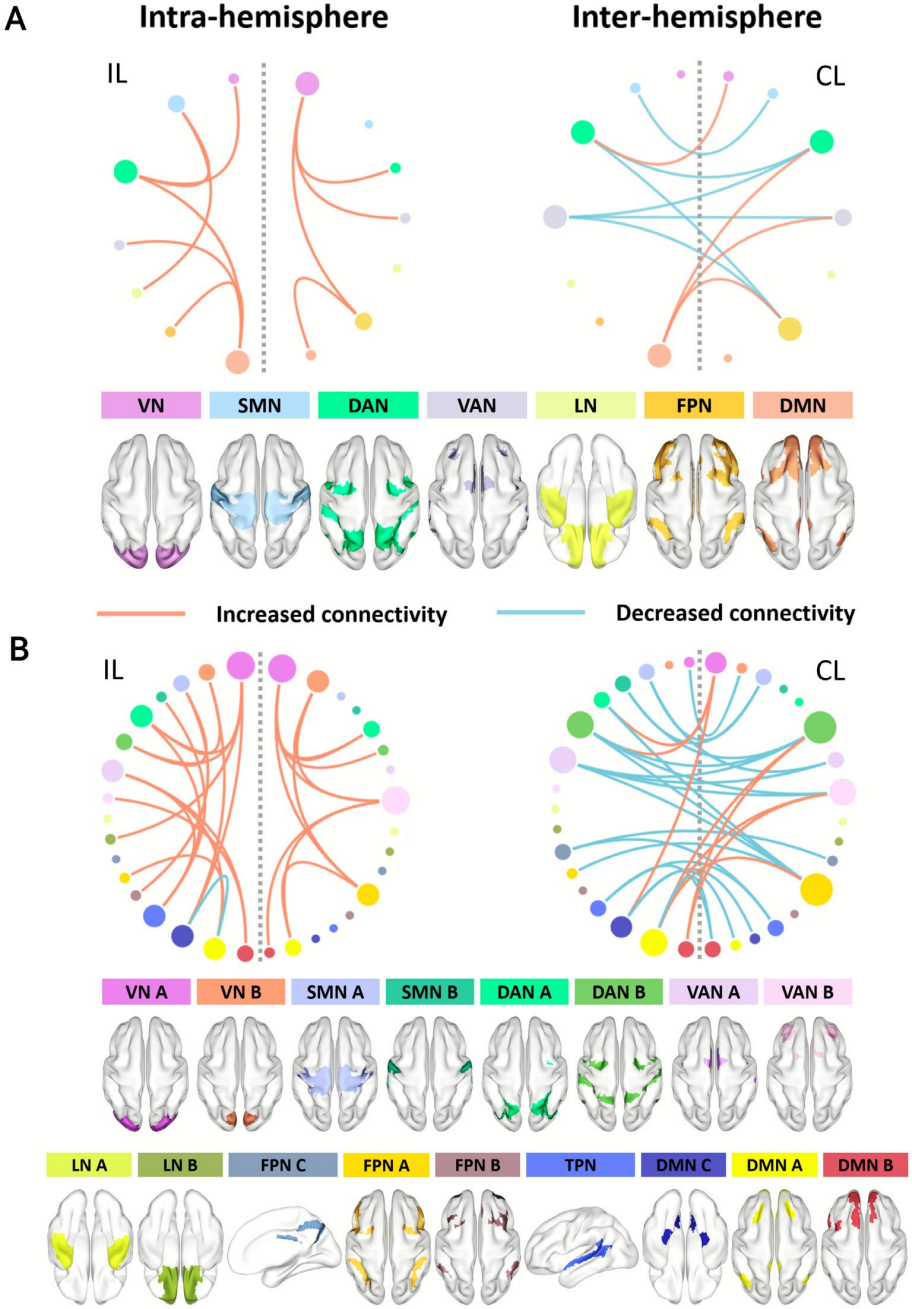
Between‐group differences in cerebral inter‐module functional connectivity. The ischemic stroke patients showed disrupted intra‐hemispheric (left) and inter‐hemispheric (right) connectivity between cerebral modules in both (A) 7‐module and (B) 17‐module configurations. The red line indicates significantly increased connectivity in the Stroke group compared to the HC group. The blue line indicates significantly decreased connectivity in the Stroke group compared to the HC group. The gray dashed line separates the ipsilesional hemisphere (IL) and the contralesional hemisphere (CL). DAN, dorsal attention network; DMN, default mode network; FPN, frontoparietal network; LN, limbic network; SMN, somatomotor network; TPN, temporal parietal network; VAN, ventral attention network; VN, visual network.

The disruptions of cerebral inter‐module functional connectivity in stroke patients were mainly manifested as increased intra‐hemispheric and decreased inter‐hemispheric connectivity (Figure 1; Table S1–S3).

For 7 modules, Stroke patients showed 20 disrupted connectivity pairs compared with HC (*p* < 0.01, FDR corrected), including 10 pairs of increased intra‐hemispheric connections (6 in the ipsilesional hemisphere (IL) and 4 in the contralesional hemisphere (CL)) and 10 pairs of inter‐hemispheric changes (6 decreases and 4 increases) (Table S2). These disruptions were primarily involved the DAN (10) and DMN (7) (Figure 1A; Table S3).

For 17 modules, Stroke patients exhibited 55 pairs of abnormal connectivity as compared to HC (*p* < 0.01, FDR corrected): 25 increased intra‐hemispheric (15 in IL, 10 in CL) with 1 decreased ipsilesional connection, plus 29 inter‐hemispheric connections (21 decreases and 8 increases) (Table S2). These abnormalities were mainly connected with DMN (24) and DAN (19) (Figure 1B; Table S3).

#### 3.2.2 Alterations in Cerebellar Inter‐Module Functional Connectivity

**FIGURE 2:**
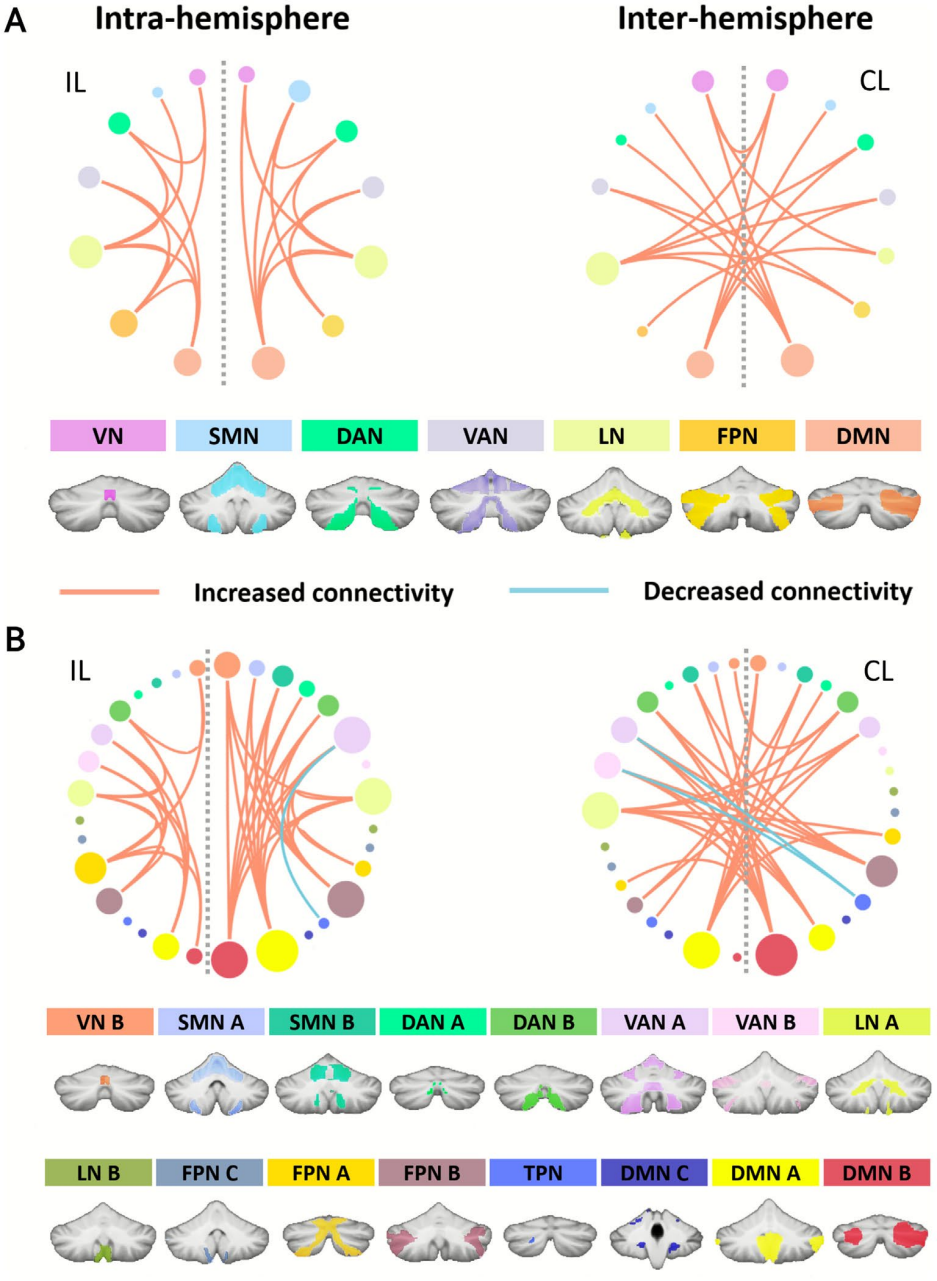
Between‐group differences in cerebellar inter‐module functional connectivity. The ischemic stroke patients showed disrupted intra‐hemispheric (left) and inter‐hemispheric (right) connectivity between cerebellar modules in both (A) 7‐module and (B) 17‐module configurations. The red line indicates significantly increased connectivity in the Stroke group compared to the HC group. The blue line indicates significantly decreased connectivity in the Stroke group compared to the HC group. The gray dashed line separates the ipsilesional hemisphere (IL) and the contralesional hemisphere (CL). DAN, dorsal attention network; DMN, default mode network; FPN, frontoparietal network; LN, limbic network; SMN, somatomotor network; TPN, temporal parietal network; VAN, ventral attention network; VN, visual network.

Enhancement in cerebellar inter‐module functional connectivity was mainly observed among cerebellar modules in stroke patients (Figure 2; Table S1–S3).

For 7 modules, the Stroke patients demonstrated 40 pairs of increased connectivity compared to HC (*p* < 0.01, FDR corrected), including 23 pairs of intra‐hemispheric connections (11 in IL and 12 in CL) and 17 pairs of inter‐hemispheric communications (Table S2). These disruptions were primarily associated with DMN (18) and LN (17) (Figure 2A; Table S3).

For 17 modules, the Stroke patients exhibited 70 pairs of abnormal connectivity compared to HC (*p* < 0.01, FDR corrected), which included 38 pairs of increased intra‐hemispheric connections (15 in IL, 23 in CL), 1 pair of decreased connectivity within the contralesional hemisphere (TPN with VAN A), and 31 pairs of inter‐hemispheric connections (29 increases and 2 decreases: CL TPN with IL VAN) (Table S2). These abnormalities were predominantly linked to the DMN (36) and FPN (27) (Figure 2B; Table S3).

#### 3.2.3 Alterations in Cerebrocerebellar Inter‐Module Functional Connectivity

**FIGURE 3:**
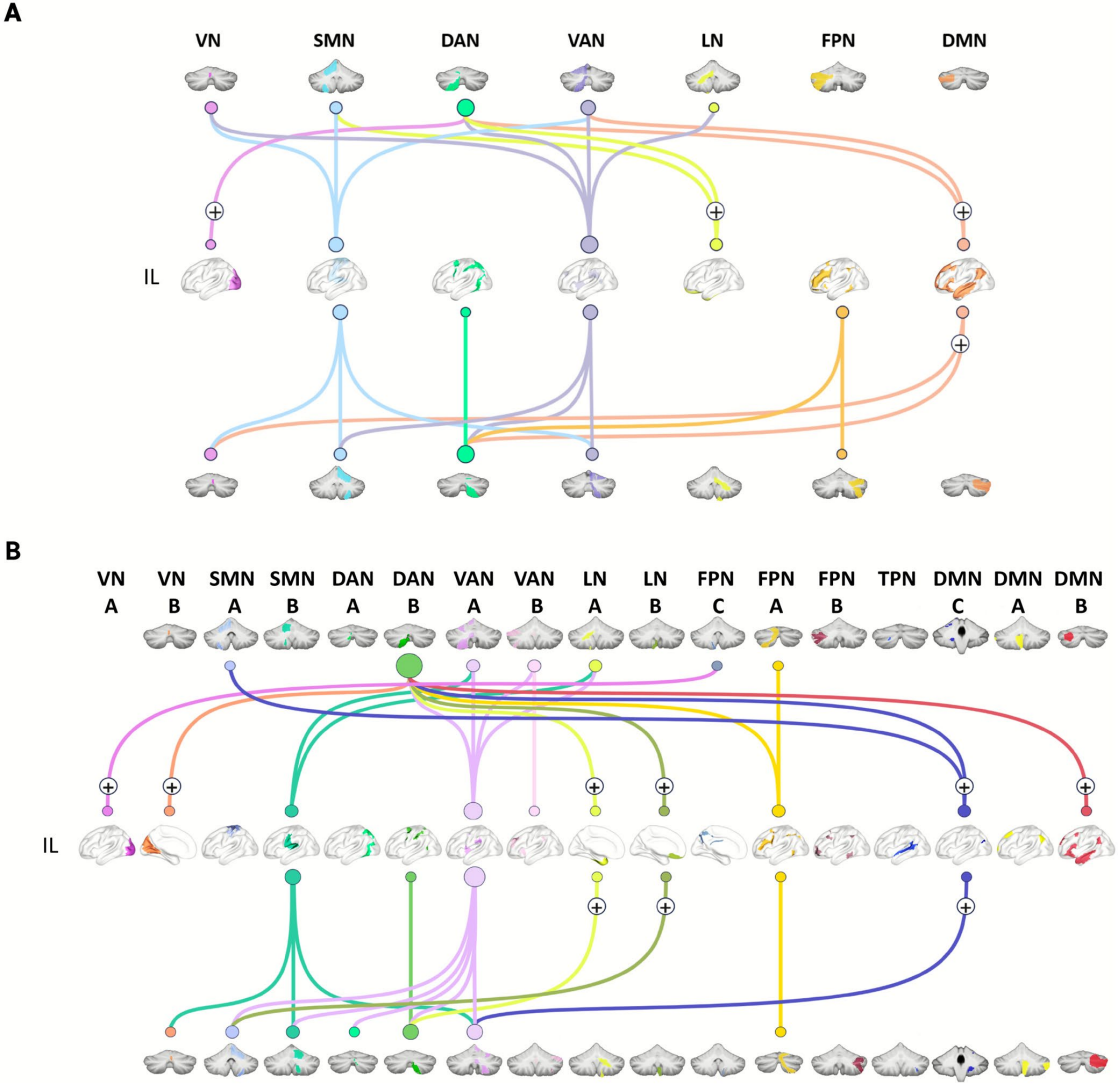
Between‐group differences in functional connectivity between ipsilesional cerebral modules and bilateral cerebellar modules. The ischemic stroke patients primarily exhibited decreased connectivity between ipsilesional (IL) cerebral modules and bilateral cerebellar modules in both (A) 7‐module and (B) 17‐module configurations. The plus sign indicates significantly increased connectivity in the Stroke group compared to the HC group. DAN, dorsal attention network; DMN, default mode network; FPN, frontoparietal network; LN, limbic network; SMN, somatomotor network; TPN, temporal parietal network; VAN, ventral attention network; VN, visual network.

**FIGURE 4:**
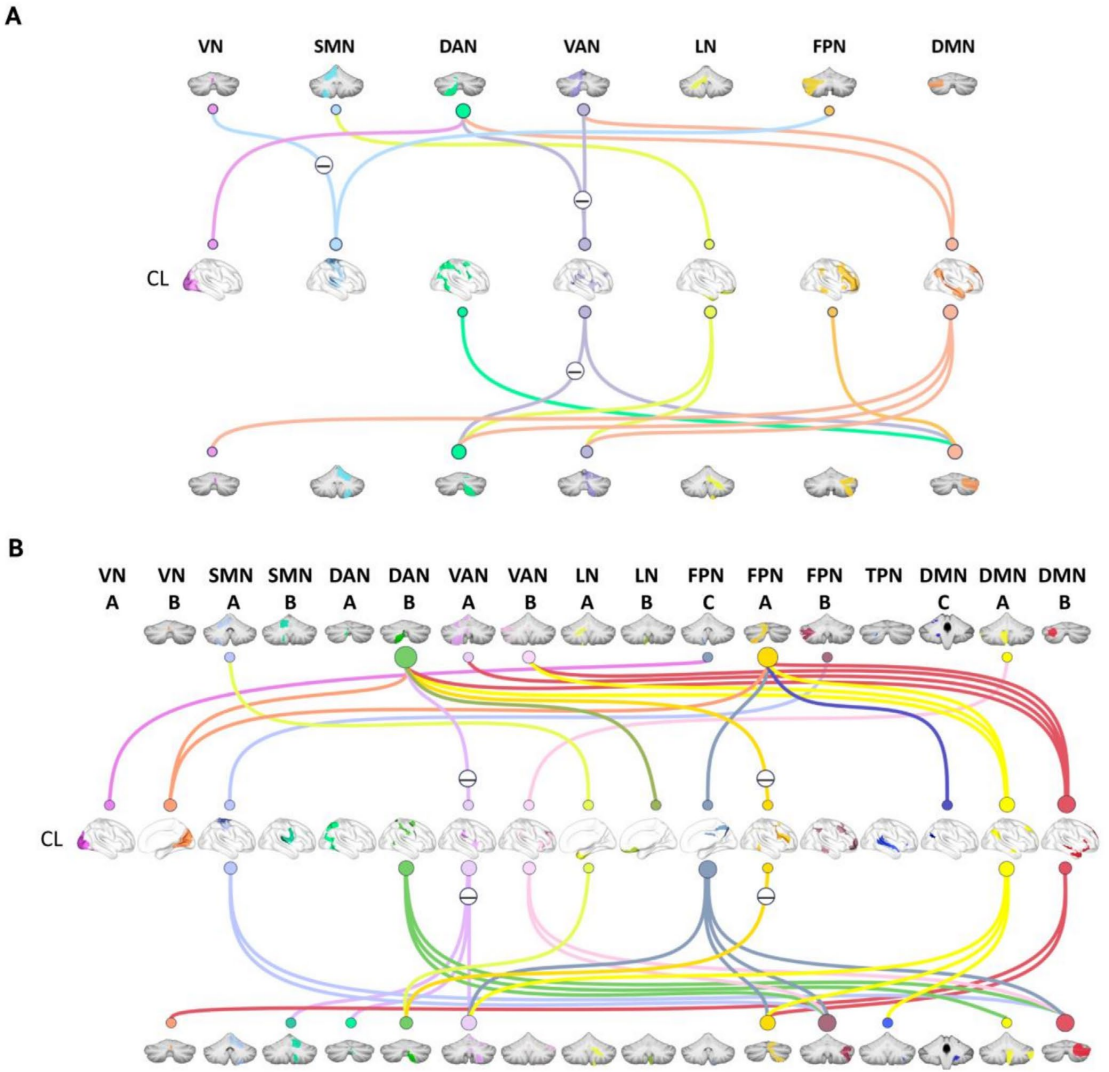
Between‐group differences in functional connectivity between contralesional cerebral modules and bilateral cerebellar modules. The ischemic stroke patients primarily exhibited increased connectivity between contralesional (CL) cerebral modules and bilateral cerebellar modules in both (A) 7‐module and (B) 17‐module configurations. The minus sign indicates significantly decreased connectivity in the Stroke group compared to the HC group. DAN, dorsal attention network; DMN, default mode network; FPN, frontoparietal network; LN, limbic network; SMN, somatomotor network; TPN, temporal parietal network; VAN, ventral attention network; VN, visual network.

Significant group differences were observed in cerebrocerebellar inter‐module functional connectivity, primarily exhibited as decreased connectivity between ipsilesional cerebral modules and bilateral cerebellar modules (Figure 3; Table S1–S3), while there was increased connectivity between contralesional cerebral modules and bilateral cerebellar modules (Figure 4; Table S1–S3).

Specifically, compared with HC, the Stroke patients showed 23 pairs of abnormal connectivity between ipsilesional cerebral modules and cerebellar modules in 7‐module condition, with 11 pairs connecting with the contralateral cerebellum (9 decreases vs. 2 increases) and 12 pairs with the ipsilateral cerebellum (7 decreases vs. 5 increases) (Table S2). Increased connectivity primarily involved the ipsilesional cerebral DMN (4), cerebellar DAN (4), while decreases were more frequent in the cerebral VAN (7) and SMN (6), cerebellar DAN (4) and VAN (4) (Figure 3A; Table S3). Similarly, in the 17‐module condition, 29 pairs of altered connections were observed, with 13 pairs connected to the contralateral cerebellum (10 decreased, 3 increased) and 16 pairs to the ipsilateral cerebellum (9 decreased, 7 increased) (Table S2). Here, increased connectivity predominantly involved the ipsilesional cerebral DMN (4) and LN (4), cerebellar DAN (6), while decreased connections were mainly connected with cerebral VAN (10), cerebellar VAN (6) and DAN (5) (Figure 3B; Table S3).

Additionally, the Stroke patients showed 17 pairs of abnormal connectivity between contralesional cerebral modules and cerebellar modules in 7 modules condition. Among these, 8 pairs connected with the contralateral cerebellum (5 increases vs. 3 decreases) and 9 pairs with the ipsilateral cerebellum (8 increases vs. 1 decrease) (Table S2). Most increased connections were associated with contralesional cerebral DMN (5) and cerebellar DAN (4), while decreased connections were mainly connected with cerebral VAN (3) and cerebellar DAN (2) (Figure 4A; Table S3). Meanwhile, the Stroke patients exhibited 39 pairs of altered connections between contralesional cerebral modules and cerebellar modules compared to HC in 17 modules condition, which included 18 pairs connecting with the contralateral cerebellum (16 increases vs. 2 decreases) and 21 pairs to the ipsilateral cerebellum (17 increases vs. 4 decreases) (Table S2). Most increased connections were connected with contralesional cerebral DMN (13) and cerebellar FPN (14), while decreased connections were mainly linked to the cerebral VAN (4) and cerebellar DAN (4) (Figure 4B; Table S3).

Notably, the reported stroke‐related alterations in module‐level functional connectivity were largely reproducible after correcting for age, gender, MMSE, and education (Table S1), and were consistent across lesion laterality subgroups (Table S4). Additionally, the robustness of these between‐group differences was further confirmed as they remained largely unchanged when using preprocessing pipelines that either included global signal regression, motion scrubbing, or when the hemodynamic lag correction step was omitted (Table S5).

### 3.3 Connectivity‐Clinical Relationships in Stroke

**FIGURE 5:**
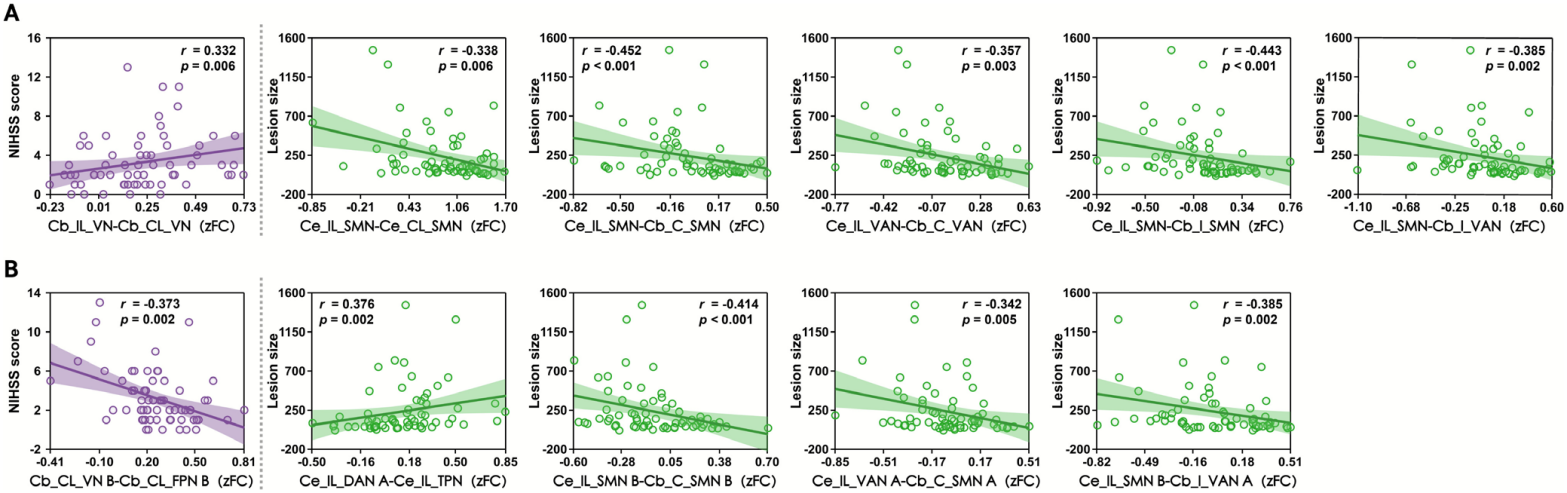
Spearman correlation between inter‐module functional connectivity and clinical measures (NIHSS score/lesion size). Exploratory Spearman correlation analyses (uncorrected for multiple comparisons) showed that cerebellar‐related connectivity changes were significantly associated with lesion size or NIHSS score in the ischemic stroke patients for both (A) 7‐module and (B) 17‐module parcellations. The vertical gray dashed line demarcates the scatter plots: NIHSS score vs. functional connectivity (left) and lesion size vs. functional connectivity (right). C, contralateral; Cb, cerebellum; Ce, cerebrum; CL, contralesional; DAN, dorsal attention network; FPN, frontoparietal network; HC, healthy controls; I, ipsilateral; IL, ipsilesional; NIHSS, National Institutes of Health Stroke Scale; SMN, somatomotor network; Stroke, stroke patients; TPN, temporal parietal network; VAN, ventral attention network; VN, visual network; zFC, z‐values of functional connectivity.

Exploratory Spearman correlation analyses showed that alterations in cerebrocerebellar connectivity were associated with lesion size, while cerebellar connectivity changes were related to NIHSS score in the Stroke patients in both 7‐ and 17‐module configurations (Figure 5; Tables S6 and S7).

Specifically, for 7 modules, four cerebrocerebellar connectivity with SMN and VAN showed significant negative correlations with lesion size (*r* = −0.36 to −0.45). Similarly, one cerebral inter‐hemispheric SMN connectivity displayed negative correlation with lesion size (*r* = −0.34). Only two connections survived after FDR correction (*p* < 0.05), namely, the cerebral inter‐hemispheric SMN and the ipsilesional cerebral SMN to contralateral cerebellar SMN connectivity. In contrast, a single cerebellar inter‐hemispheric VN connectivity exhibited positive correlation with NIHSS score (*r* = 0.33) (all *p* < 0.01; Figure 5A; Table S6).

Similarly, for 17 modules, three cerebrocerebellar connectivity with SMN and VAN were negatively correlated (*r* = −0.34 to −0.41), while one cerebral connectivity between ipsilesional DAN and TPN positively was correlated (*r* = 0.38) with lesion size. Notably, a cerebellar connectivity between contralesional VN and FPN demonstrated a negative correlation with NIHSS score (*r* = −0.37) (all *p* < 0.01; Figure 5B; Table S6).

It should be noted that these correlational findings are exploratory and should be interpreted with caution. Moreover, the observed correlation results remained largely unchanged after correcting for age, sex, MMSE, education, and days since onset (Table S6).

## 4 Discussion

This study applied connectivity‐based analyses to systematically explore alterations in cerebral, cerebellar, and cerebrocerebellar connectivity between different functional networks/modules in patients with basal ganglia ischemic stroke. The results revealed: (1) disrupted cerebral connectivity (increased intra‐hemispheric but decreased inter‐hemispheric connections); (2) enhanced cerebellar inter‐module connectivity; and (3) asymmetric cerebrocerebellar connectivity (decreased ipsilesional but increased contralesional connections). Notably, these cerebellar‐related connectivity changes were correlated with lesion size and clinical severity in our exploratory analyses. These findings emphasize the potential importance of investigating disruptions in cerebellar connectivity in ischemic stroke.

### 4.1 Alterations in Cerebral Inter‐Module Functional Connectivity

Compared to HC, the Stroke patients mainly exhibited increased intra‐hemispheric while decreased inter‐hemispheric connectivity between cerebral modules. The enhanced intra‐hemispheric connections likely represent compensatory plasticity for impaired contralesional connectivity or disrupted transcallosal inhibition [6, 42], while reduced interhemispheric connectivity reflects structural damage like transcallosal fiber degeneration post‐stroke [15, 43]. Our findings align with previous studies [6, 13, 14, 15, 43], reinforcing that such neural changes may serve as markers of post‐stroke functional reorganization. Moreover, these connectivity disruptions were primarily connected with DMN and DAN. The observed abnormalities in the DAN—a key network for attention control—may contribute to post‐stroke spatial neglect [5, 12]. Some evidence suggests reduced DAN‐DMN connectivity could be associated with neglect symptoms [13], while DMN dysfunction (as a major resting‐state network) might relate to cognitive deficits following stroke [42]. These collective connectivity alterations could collectively represent possible mechanisms underlying impaired attention control and cognitive function, potentially highlighting the importance of network interactions in stroke recovery.

### 4.2 Alterations in Cerebellar Inter‐Module Functional Connectivity

Our first hypothesis was that basal ganglia ischemic stroke would disrupt the patterns of intra‐ and inter‐hemispheric connectivity between cerebellar modules. Our results not only supported this hypothesis but also elucidated a specific pattern of disruption: enhanced connectivity between different functional modules within the cerebellum in patients. Despite evidence for its regional specialization, the cerebellum is often treated as functionally homogeneous in neuroimaging studies [44]. A prior longitudinal study of 23 pontine stroke patients has already revealed that cerebellar regions exhibited the most frequent functional reorganization, though it treated the cerebellum as a unified network [45]. The cerebellar modular parcellation defined by Buckner et al. (2011) was originally defined in healthy individuals [20]; it was also commonly applied to explore the cerebellar abnormalities in brain disorders [46, 47, 48]. Crucially, we validated the applicability of this template to our specific stroke cohort through modularity analysis (see details in Supporting Information), which demonstrated a high agreement between the patient‐derived modular structure and the Buckner template (e.g., Adjusted Rand Index ARI = 0.71), indicating the preservation of the fundamental cerebellar functional architecture despite stroke lesions. Using this modular parcellation of cerebellum, we found the stroke patients showed increased connectivity between cerebellar modules compared to HC. To our knowledge, this is the first study to comprehensively investigate cerebellar inter‐module functional connectivity after stroke. Functional segregation refers to the brain's division into specialized, independently processing modules, with dense intra‐module connections but sparser inter‐module links [49]‐ an organization also observed in the cerebellum [19, 20]. Increased inter‐module connectivity thus suggests decreased cerebellar network segregation, implying that executing behavioral or cognitive functions may require broader network engagement due to impaired isolated processing. One possible reason for the enhanced functional connectivity between cerebellar modules in stroke patients is the compensation for structural disconnections. Previous studies have demonstrated significant reduction of indirect structural connectivity in cerebellar regions remote from the lesions [50], and such disconnections may drive compensatory enhancement of local synaptic plasticity within cerebellum. When the lesion develops in the basal ganglia, it may disrupt indirect structural connections between the basal ganglia and cerebellum (e.g., via thalamic or pontine relay pathways) [51]. To compensate for this disconnection, intra‐cerebellar connections may be strengthened, which also aligns with Olafson et al.'s (2021) observation that less structurally damaged regions upregulate functional connectivity to facilitate functional recovery [45]. All these findings suggest that cerebral infarction triggers reorganization not only in cerebral but also in cerebellar functional networks, marked by heightened intra‐ and inter‐hemispheric cerebellar connectivity. Moreover, most connections were connected to the cerebellar LN, FPN, and DMN, which may indicate the cerebellum's role beyond motor control—extending to higher‐order cognitive functions and emotional processing [17, 18, 52].

### 4.3 Alterations in Cerebrocerebellar Inter‐Module Functional Connectivity

Our second hypothesis proposed that basal ganglia ischemic stroke leads to abnormal inter‐module cerebrocerebellar connectivity, extending beyond the ipsilesional cortical‐cerebellar circuit with significant compromise in contralesional pathways. This hypothesis was robustly supported by two key findings.

First, we demonstrated that CCD‐related alterations of functional connectivity were not only between the ipsilesional cerebrum and the contralateral cerebellum, but also between the affected cerebral hemisphere and the ipsilateral cerebellum. Supratentorial lesions following ischemic stroke can induce CCD, characterized by reduced cerebellar blood flow and metabolic activity in the cerebellum contralateral to the lesions [21]. This phenomenon has its structural underpinnings in the degeneration of corticopontine pathways following focal ischemic lesions, which subsequently induces cerebellar dysfunction and serves as a key mechanism of CCD [22]. Pontine infarction studies further confirm that disrupted cerebro‐cerebellar functional connectivity specifically depends on cortico‐ponto‐cerebellar anatomical pathway integrity [25]. Beyond this classical circuitry, emerging evidence highlights two additional pathways: First, the dentato‐thalamo‐cortical tract (DTCT), the principal cerebellar efferent pathway, whose structural integrity strongly correlates with post‐stroke motor recovery [26]. Second, the dorsal spinocerebellar tract (DSCT) and inferior cerebellar peduncle (ICP)‐mediated proprioceptive inputs, where ICP degeneration after supratentorial stroke may exacerbate cerebellar hypofunction through spinocerebellar deafferentation, offering a novel perspective on CCD pathophysiology [53]. Thus, we propose that our findings of decreased connectivity between ipsilesional cerebrum and cerebellum may be attributed to the structural disconnections caused by basal ganglia lesions through thalamic or pontine relay pathways [51]. These changes predominantly involved the cerebral VAN and SMN, as well as the cerebellar DAN and VAN, potentially contributing to the characteristic motor, cognitive, and attentional impairments observed in stroke patients. The network‐specific connectivity reductions may suggest impaired information transfer between critical motor control and attention‐related regions [25].

Second, connectivity changes between the contralesional cerebral hemisphere and bilateral cerebellar modules were mainly increased. This likely represents a compensatory mechanism following focal brain injury, where the intact hemisphere and cerebellum assume functions previously mediated by the damaged regions [24, 25, 52]. Such cross‐hemispheric communication is vital for functional recovery, with the cerebellum potentially facilitating this process by enhancing connections to the contralesional hemisphere [54]. These changes primarily involved the cerebral DMN and the cerebellar DAN and FPN. Increased DMN connectivity may reflect adaptive reorganization to preserve cognitive function [42]. Additionally, stronger DAN engagement could help maintain focus and complete tasks [13], while strengthened FPN connectivity may support cognitive control and task performance, which are both crucial for post‐stroke recovery [55]. These adaptive reorganization patterns likely help preserve executive functions and facilitate cognitive reorganization after stroke.

Altogether, our findings further illustrate that supratentorial infarction causes widespread cerebrocerebellar connectivity disruptions that extend beyond classical CCD patterns. This underscores the significance of functional network analysis, as its sensitivity enables the detection of more widespread abnormalities in brain connectivity, offering a deeper understanding of the extensive effects of stroke on brain networks.

### 4.4 Relationship Between Abnormal FC and Lesion Size, Disease Severity

Previous studies have reported conflicting results regarding whether lesion size [56, 57], location [28, 58], or disrupted pathways [56] are associated with CCD. While some link CCD to NIHSS score [59, 60], others argue it depends on impaired neurons and cortical‐pontine‐cerebellar pathway disruption [60]. These inconsistencies may arise from methodological differences [28]. By recruiting patients with basal ganglia ischemic stroke, our exploratory analyses indicated that connectivity changes were significantly associated with lesion size or stroke severity in patients primarily involved connections with the cerebellum, suggesting the potential importance of cerebellar‐related connectivity changes in understanding the functional damage following stroke.

Specifically, our analysis identified two key cerebellar connectivity patterns. First, larger infarct volumes were significantly associated with extensive disruption of cerebrocerebellar connections, particularly those involving the SMN and VAN, suggesting that these long‐range pathways integrating crucial sensory and attentional signals are highly vulnerable to lesion expansion. Second, under the more refined 17‐module parcellation scheme, weakened connectivity between the contralateral cerebellar VN and FPN was strongly correlated with more severe neurological deficits (i.e., higher NIHSS scores). Previous studies have demonstrated that disrupted FPN‐related connectivity constitutes an important substrate for post‐stroke cognitive impairment [61, 62]. Given the essential role of frontoparietal–visual network synergy in higher‐order cognitive processes such as attention and spatial awareness, the disruption of this specific connection may constitute a neural substrate linked to post‐stroke cognitive and perceptual deficits, which in turn may be associated with greater clinical severity.

Although no dedicated motor function scales were employed in this study, 56 of the 65 enrolled patients (86%) exhibited limb motor impairments, such as paralysis, weakness, or numbness. We thus suspect that the observed cerebellar‐related connectivity alterations may be related to motor deficits, especially for those connections with SMN. These findings are consistent with the growing interest in cerebellar neuromodulation for motor dysfunctions. Several recent studies support the cerebellum as an independent and promising therapeutic target: cerebellar intermittent theta‐burst stimulation (iTBS) promotes motor recovery through mechanisms distinct from those of primary motor cortex (M1) stimulation [63]; cerebellar transcranial direct current stimulation (tDCS) may be superior to M1 stimulation for gait improvement [64] and also enhances upper limb functional recovery [65]. Emerging frameworks further propose combining cerebellar neuromodulation with specific behavioral training [66], and multi‐focal stimulation strategies targeting both cerebellar and cerebral nodes have shown preliminary potential [67]. Although challenges remain, including the unclear mechanisms of cerebellar transcranial alternating current stimulation (tACS) improving walking ability through gamma oscillation modulation [68] and the need for more robust clinical evidence [69], the therapeutic value of cerebellar pathways is increasingly recognized.

In summary, the cerebellar connectivity patterns identified in our study not only suggest their potential as biomarkers for stroke severity but also provide a new theoretical basis for targeting these pathways in personalized rehabilitation. Future interventions, such as cerebellar tDCS, tACS, or iTBS, could be tailored to individual connectome profiles to enhance functional recovery. It is important to note that as the correlational findings in this study were not corrected for multiple comparisons, the above conclusions require careful validation in future research.

## 5 Limitations

This study has several limitations. First, we used a cross‐sectional design to investigate disruptions of cerebellar connectome in ischemic stroke patients within 1 month of onset. Future longitudinal studies are needed to further explore how connectivity patterns change over time after stroke. Second, while focusing on basal ganglia ischemic stroke patients facilitated observation of cerebrocerebellar functional reorganization (due to high CCD incidence) [27, 28], this selective inclusion may limit the generalizability of our findings to other stroke subtypes (e.g., cortical or cerebellar strokes). Future studies would benefit from including a more diverse patient population. Third, exploring the relationship between the alterations of cerebellar connectivity and functional recovery would be important for stroke rehabilitation. However, we failed to collect functional outcomes, i.e., modified Rankin Scale (mRS) in three months following stroke, and thus could not examine whether enhanced cerebellar connectivity was related to functional recovery in these patients. We also lacked information on medications and rehabilitation interventions, making it impossible to rule out their potential effects on our findings. Future studies by collecting the follow‐up mRS scores and detailed medication history and rehabilitation interventions of the patients could help clarify these issues. Fourth, the correlation analyses were exploratory and were not corrected for multiple comparisons. To quantitatively contextualize this limitation, we conducted post hoc power and a priori sample size analyses. These analyses demonstrated that our sample size (*n* = 65) provided adequate statistical power (> 80%) to detect the stronger correlations reported in our study (specifically, those with |*r*| ≥ 0.41). The sample size was also confirmed to meet or exceed the theoretical requirement for detecting such effects. Therefore, our key significant findings are robust. However, the study was likely underpowered to detect more subtle associations (|*r*| < 0.41). Future multicenter studies with larger samples are warranted to investigate these potential smaller effects and to validate the reproducibility of our primary findings. Finally, although our inter‐module functional connectivity approach effectively characterized cerebral, cerebellar, and cerebrocerebellar connectivity changes following focal lesions, it may have missed important alterations in intra‐network/module connectivity. More comprehensive approaches using region‐level or voxel‐level connectivity analyses in future studies could provide a more complete picture of whole‐brain disconnections across diverse stroke subtypes.

## 6 Conclusion

These findings highlight the importance of investigating cerebellar connectome disruptions in ischemic stroke, which may provide valuable insights into the underlying brain mechanisms of the disease.

## Author Contributions

Y.S. and Y.L. designed the study; Y.F., Z.M., X.W., and Y.W. collected the data; J.W. and X.W. analyzed the data; X.W. wrote the manuscript; T.L. and X.W. prepared the figures; Y.Z., Y.S., and Y.L. revised the manuscript. All authors read and approved the final manuscript.

## Funding

This work was supported by the Zhejiang Provincial Natural Science Foundation of China (No. LGJ22H180001), National Key R&D Program of China (No. 2017YFC1310000), The Construction Fund of Key Medical Disciplines of HangZhou (No. 2025HZGF02), and Medical and Health Science Program of Zhejiang Province (No. 2025HY0687).

## Ethics Statement

This study was approved by the Ethics Committee of the Center for Cognition and Brain Disorders, Hangzhou Normal University (Approval ID: 20171027), and all participants signed informed consent.

## Consent

The authors have nothing to report.

## Conflicts of Interest

The authors declare no conflicts of interest.

## Supporting information


**Figure S1:** Overlapping lesions for ischemic stroke patients.
**Table S1:** Significant between‐group differences in module‐level functional connectivity with differing covariate adjustments.
**Table S2:** The summary of significant between‐group differences in module‐level functional connectivity.
**Table S3:** Frequency of stroke‐related functional connectivity changes between key networks across brain regions.
**Table S4:** Subgroup analyses by lesion laterality: between‐group differences relative to healthy controls.
**Table S5:** Robustness analysis: modifying preprocessing steps.
**Table S6:** Significant Spearman correlation between connectivity metrics and clinical variables in Stroke.
**Table S7:** Exploratory Spearman correlations between functional connections and clinical variables.

## Data Availability

The data that support the findings of this study are available from the corresponding author upon reasonable request.
