# Supplementary material for: The Cerebellar Connectome Disruptions in Ischemic Stroke
Source: CNS Neurosci Ther. 2026 Jan 23;32(1):e70759. doi: 10.1002/cns.70759 (PMC12828662; doi:10.1002/cns.70759)
Supplement: Supplementary file 1 — Figure S1: Overlapping lesions for ischemic stroke patients. Table S1: Significant between‐group differences in module‐level functional connectivity with differing covariate adjustments. Table S2: The summary of significant between‐group differences in module‐level functional connectivity. Table S3: Frequency of stroke‐related functional connectivity changes between key networks across brain regions. Table S4: Subgroup analyses by lesion laterality: between‐group differences relative to healthy controls. Table S5: Robustness analysis: modifying preprocessing steps. Table S6: Significant Spearman correlation between connectivity metrics and clinical variables in Stroke. Table S7: Exploratory Spearman correlations between functional connections and clinical variables. [file CNS-32-e70759-s001.docx]

**Supplementary Materials**

**Contents**

[**MRI data acquisition** 2](#_Toc217399747)

[**Hemodynamic Lag Correction** 3](#_Toc217399748)

[**Validation of Buckner Cerebellar Parcellation in Stroke Cohort** 4](#_Toc217399749)

[**Figure S1. Overlapping lesions for ischemic stroke patients.** 5](#_Toc217399750)

[**Table S1. Significant between-group differences in module-level functional connectivity with differing covariate adjustments.** 6](#_Toc217399751)

[**Table S2. The summary of significant between-group differences in module-level functional connectivity.** 17](#_Toc217399752)

[**Table S3. Frequency of stroke-related functional connectivity changes between key networks across brain regions.** 18](#_Toc217399753)

[**Table S4. Subgroup analyses by lesion laterality: between-group differences relative to healthy controls.** 19](#_Toc217399754)

[**Table S5. Robustness analysis: modifying preprocessing steps.** 30](#_Toc217399755)

[**Table S6. Significant Spearman correlation between connectivity metrics and clinical variables in Stroke.** 48](#_Toc217399756)

[**Table S7. Exploratory Spearman correlations between functional connections and clinical variables.** 50](#_Toc217399757)

**MRI data acquisition**

Rs-fMRI, structural MRI (sMRI), and diffusion-weighted imaging (DWI) data were collected from all participants using a 3T scanner (GE MR-750, Waukesha, WI) at Anshan Changda Hospital. During the scanning, participants were instructed to keep their eyes closed, remain still, avoid falling asleep, and refrain from thinking about anything.

**Rs-fMRI data**. The rs-fMRI data were collected using an echo-planar imaging sequence with axial slice = 43, slice thickness/gap = 3.2/0 mm, echo time (TE) = 30 ms, repetition time (TR) = 2000 ms, field of view (FOV) = 220 × 220 mm^2^, matrix size = 64 × 64, voxel size = 3.4 mm × 3.4 mm × 3.2 mm, flip angle (FA) = 90°, volumes = 240, time = 8’, and parallel acceleration = 2.

**sMRI data**. The sMRI data were obtained using a 3D-MPRAGE sequence with sagittal slice = 176, slice thickness/gap = 1/0 mm, TE = 3.1 ms, TR = 8100 ms, FOV = 256 × 256 mm^2^, matrix size = 256 × 256, voxel size = 1 mm × 1 mm × 1 mm, FA = 8°, and time = 5’05”.

**DWI data**. The DWI data were acquired with axial slice = 22, slice thickness/gap = 5/1 mm, TR = 4000 ms, FOV = 240 × 240 mm^2^, b = 1000, and time = 1’.

**Hemodynamic Lag Correction**

To address known connectivity artifacts arising from altered hemodynamics in ischemic stroke, we performed a voxel-wise hemodynamic lag correction using a time-shift analysis approach. The mean signal from the whole brain was calculated as the reference time course. For each voxel within the brain, its time course was extracted and shifted within a window of -4 TR to +4 TR (i.e., -8 s to +8 s, TR = 2 s). The correlation coefficient between the shifted time course of each voxel and the reference time course was calculated at each TR step. Each voxel was then assigned the time shift value that yielded the maximal correlation coefficient. Then the time series of each voxel was shifted in time according to its individually determined lag value. All subsequent functional connectivity analyses were conducted using these corrected time series.

**Validation of Buckner Cerebellar Parcellation in Stroke Cohort**

To validate the applicability of the healthy population-derived Buckner parcellation to our specific stroke patient cohort, we performed a supplementary modularity analysis. We conducted module detection 100 times using the Louvain algorithm on the voxel-wise cerebellar functional connectivity matrices from our patient data, which yielded a mean modularity index (Q) of 0.32 and a mean number of communities of 5.75. This indicates a robust modular structure within our patient data.

We then assessed the agreement between the patient-derived modules and the Buckner 7-network template by calculating four similarity metrics: the Normalized Mutual Information (NMI, which measures shared information between partitions), the Rand Index (RI, which calculates the proportion of agreeing sample pairs), the Adjusted Rand Index (ARI, a chance-corrected version of RI), and the Gamma Index (which assesses similarity based on the concordance of relative node-pair orders and is particularly suited for evaluating community structure match). The results (NMI: 0.73 ± 0.10; RI: 0.91 ± 0.04; ARI: 0.71 ± 0.13; Gamma: 0.71 ± 0.13) demonstrate that, despite the presence of stroke lesions, the overall architecture of the cerebellar functional network in patients remained highly consistent with the healthy template. This provides empirical support for the use of the Buckner parcellation in this study.


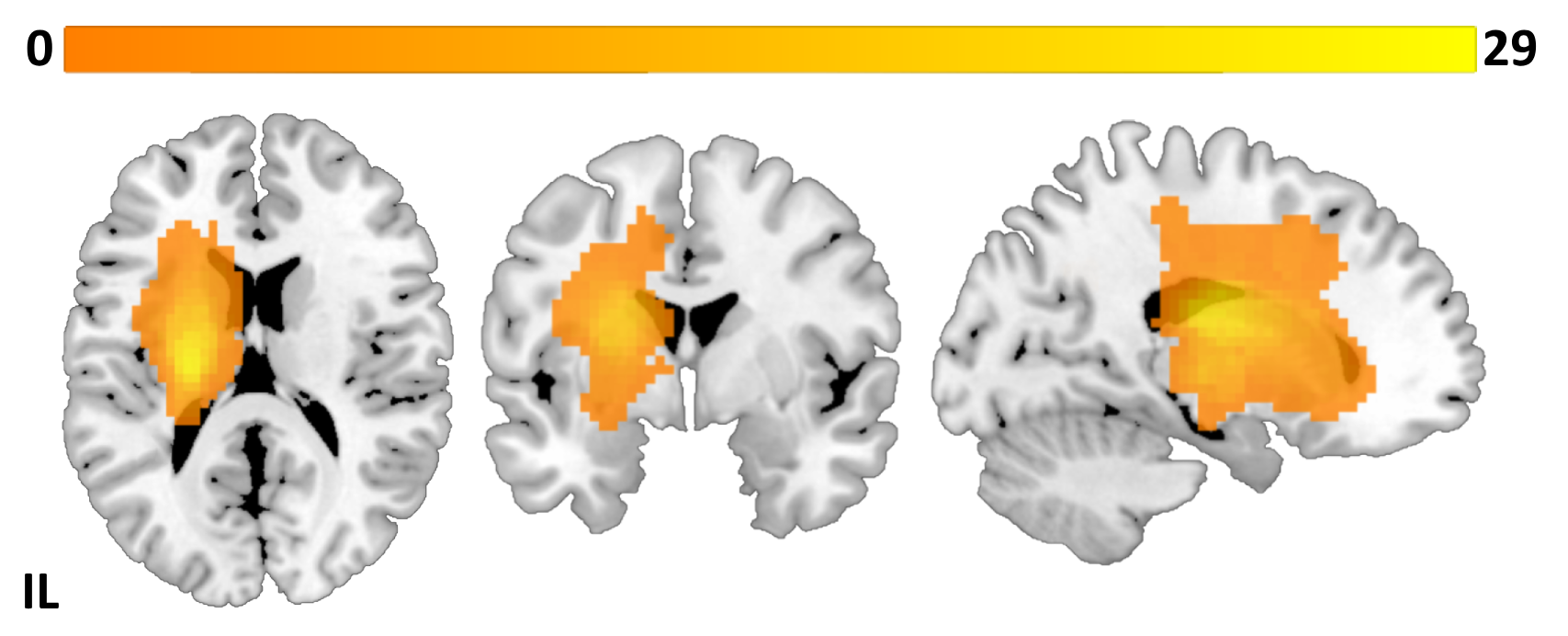


**Figure S1. Overlapping lesions for ischemic stroke patients.** IL, ipsilesional hemisphere.

**Table S1. Significant between-group differences in module-level functional connectivity with differing covariate adjustments.**

|  |  | **Stroke vs. HC†** | | **Stroke vs. HC‡** | |
| --- | --- | --- | --- | --- | --- |
|  | **Connections** | **Δ** | ***p*** | **Δ** | ***p*** |
| **7 modules** | |  |  |  |  |
| 1 | Ce_IL_VN-Ce_IL_DAN | 0.2051 | 0.0001 | 0.1149 | 0.0071 |
| 2 | Ce_IL_SMN-Ce_IL_DAN | 0.1639 | 0.0005 | 0.0965 | 0.0247 |
| 3 | Ce_IL_SMN-Ce_IL_LN | 0.1353 | 0.0004 | 0.0904 | 0.0112 |
| 4 | Ce_IL_DAN-Ce_IL_DMN | 0.1493 | 0.0017 | 0.0848 | 0.0425 |
| 5 | Ce_IL_VAN-Ce_IL_DMN | 0.1924 | 0.0006 | 0.1171 | 0.0215 |
| 6 | Ce_IL_FPN-Ce_IL_DMN | 0.1844 | 0.0002 | 0.1186 | 0.0056 |
| 7 | Ce_CL_VN-Ce_CL_DAN | 0.2307 | 0.0001 | 0.1398 | 0.0016 |
| 8 | Ce_CL_VN-Ce_CL_VAN | 0.1500 | 0.0006 | 0.0746 | 0.0468 |
| 9 | Ce_CL_VN-Ce_CL_FPN | 0.2363 | 0.0001 | 0.1228 | 0.0050 |
| 10 | Ce_CL_FPN-Ce_CL_DMN | 0.2095 | 0.0001 | 0.1528 | 0.0010 |
| 11 | Ce_IL_SMN-Ce_CL_SMN | -0.2866 | 0.0002 | -0.1581 | 0.0153 |
| 12 | Ce_IL_DAN-Ce_CL_VN | 0.2048 | 0.0001 | 0.1327 | 0.0044 |
| 13 | Ce_IL_DAN-Ce_CL_DAN | -0.1957 | 0.0001 | -0.0996 | 0.0261 |
| 14 | Ce_IL_DAN-Ce_CL_FPN | -0.1341 | 0.0019 | -0.0960 | 0.0222 |
| 15 | Ce_IL_VAN-Ce_CL_DAN | -0.1952 | 0.0002 | -0.1727 | 0.0006 |
| 16 | Ce_IL_VAN-Ce_CL_VAN | -0.3240 | 0.0001 | -0.2273 | 0.0010 |
| 17 | Ce_IL_VAN-Ce_CL_FPN | -0.1875 | 0.0001 | -0.1303 | 0.0026 |
| 18 | Ce_IL_DMN-Ce_CL_DAN | 0.1848 | 0.0002 | 0.1049 | 0.0139 |
| 19 | Ce_IL_DMN-Ce_CL_VAN | 0.1746 | 0.0007 | 0.1053 | 0.0213 |
| 20 | Ce_IL_DMN-Ce_CL_FPN | 0.1679 | 0.0006 | 0.1240 | 0.0063 |
| 21 | Cb_IL_VN-Cb_IL_DAN | 0.1199 | 0.0009 | 0.0717 | 0.0236 |
| 22 | Cb_IL_VN-Cb_IL_FPN | 0.1473 | 0.0002 | 0.0617 | 0.0670 |
| 23 | Cb_IL_SMN-Cb_IL_LN | 0.1428 | 0.0023 | 0.0786 | 0.0661 |
| 24 | Cb_IL_DAN-Cb_IL_LN | 0.1856 | 0.0001 | 0.0737 | 0.0575 |
| 25 | Cb_IL_DAN-Cb_IL_DMN | 0.1921 | 0.0003 | 0.1050 | 0.0233 |
| 26 | Cb_IL_VAN-Cb_IL_LN | 0.1603 | 0.0004 | 0.0580 | 0.1101 |
| 27 | Cb_IL_VAN-Cb_IL_FPN | 0.2217 | 0.0002 | 0.0844 | 0.0757 |
| 28 | Cb_IL_VAN-Cb_IL_DMN | 0.2468 | 0.0001 | 0.1346 | 0.0127 |
| 29 | Cb_IL_LN-Cb_IL_FPN | 0.1821 | 0.0001 | 0.0839 | 0.0255 |
| 30 | Cb_IL_LN-Cb_IL_DMN | 0.1157 | 0.0018 | 0.0810 | 0.0226 |
| 31 | Cb_IL_FPN-Cb_IL_DMN | 0.1808 | 0.0008 | 0.1408 | 0.0068 |
| 32 | Cb_CL_VN-Cb_CL_LN | 0.1281 | 0.0002 | 0.0551 | 0.0410 |
| 33 | Cb_CL_VN-Cb_CL_DMN | 0.1363 | 0.0001 | 0.0687 | 0.0300 |
| 34 | Cb_CL_SMN-Cb_CL_DAN | 0.1298 | 0.0012 | 0.0466 | 0.1171 |
| 35 | Cb_CL_SMN-Cb_CL_FPN | 0.1795 | 0.0002 | 0.0621 | 0.1024 |
| 36 | Cb_CL_SMN-Cb_CL_DMN | 0.2090 | 0.0001 | 0.1078 | 0.0041 |
| 37 | Cb_CL_DAN-Cb_CL_LN | 0.1217 | 0.0025 | 0.0473 | 0.1317 |
| 38 | Cb_CL_DAN-Cb_CL_DMN | 0.2247 | 0.0001 | 0.1307 | 0.0056 |
| 39 | Cb_CL_VAN-Cb_CL_LN | 0.1856 | 0.0002 | 0.1301 | 0.0032 |
| 40 | Cb_CL_VAN-Cb_CL_FPN | 0.2022 | 0.0001 | 0.0870 | 0.0445 |
| 41 | Cb_CL_VAN-Cb_CL_DMN | 0.2359 | 0.0001 | 0.1475 | 0.0018 |
| 42 | Cb_CL_LN-Cb_CL_FPN | 0.1967 | 0.0001 | 0.1210 | 0.0037 |
| 43 | Cb_CL_LN-Cb_CL_DMN | 0.2317 | 0.0001 | 0.1176 | 0.0023 |
| 44 | Cb_IL_VN-Cb_CL_VN | 0.1260 | 0.0018 | 0.0408 | 0.1579 |
| 45 | Cb_IL_VN-Cb_CL_LN | 0.1536 | 0.0001 | 0.0888 | 0.0097 |
| 46 | Cb_IL_VN-Cb_CL_DMN | 0.1308 | 0.0002 | 0.0936 | 0.0048 |
| 47 | Cb_IL_SMN-Cb_CL_DMN | 0.1698 | 0.0002 | 0.0899 | 0.0222 |
| 48 | Cb_IL_DAN-Cb_CL_DMN | 0.2110 | 0.0001 | 0.1418 | 0.0040 |
| 49 | Cb_IL_VAN-Cb_CL_FPN | 0.2050 | 0.0002 | 0.1133 | 0.0175 |
| 50 | Cb_IL_VAN-Cb_CL_DMN | 0.2980 | 0.0001 | 0.1787 | 0.0006 |
| 51 | Cb_IL_LN-Cb_CL_VN | 0.1141 | 0.0006 | 0.0467 | 0.0957 |
| 52 | Cb_IL_LN-Cb_CL_DAN | 0.1993 | 0.0001 | 0.1180 | 0.0018 |
| 53 | Cb_IL_LN-Cb_CL_VAN | 0.1724 | 0.0001 | 0.1062 | 0.0143 |
| 54 | Cb_IL_LN-Cb_CL_FPN | 0.1903 | 0.0001 | 0.1183 | 0.0045 |
| 55 | Cb_IL_LN-Cb_CL_DMN | 0.2154 | 0.0001 | 0.1301 | 0.0010 |
| 56 | Cb_IL_FPN-Cb_CL_LN | 0.1331 | 0.0022 | 0.0859 | 0.0329 |
| 57 | Cb_IL_DMN-Cb_CL_VN | 0.1409 | 0.0003 | 0.0778 | 0.0252 |
| 58 | Cb_IL_DMN-Cb_CL_SMN | 0.1217 | 0.0010 | 0.0950 | 0.0080 |
| 59 | Cb_IL_DMN-Cb_CL_DAN | 0.2012 | 0.0003 | 0.1211 | 0.0089 |
| 60 | Cb_IL_DMN-Cb_CL_VAN | 0.1767 | 0.0003 | 0.1276 | 0.0042 |
| 61 | Ce_IL_SMN-Cb_C_VN | -0.1424 | 0.0001 | -0.1093 | 0.0028 |
| 62 | Ce_IL_SMN-Cb_C_SMN | -0.1788 | 0.0003 | -0.1159 | 0.0121 |
| 63 | Ce_IL_SMN-Cb_C_VAN | -0.1380 | 0.0023 | -0.0977 | 0.0241 |
| 64 | Ce_IL_DAN-Cb_C_DAN | -0.2006 | 0.0001 | -0.1297 | 0.0031 |
| 65 | Ce_IL_VAN-Cb_C_SMN | -0.1684 | 0.0002 | -0.1005 | 0.0131 |
| 66 | Ce_IL_VAN-Cb_C_DAN | -0.1991 | 0.0001 | -0.1422 | 0.0013 |
| 67 | Ce_IL_VAN-Cb_C_VAN | -0.1579 | 0.0005 | -0.1197 | 0.0066 |
| 68 | Ce_IL_FPN-Cb_C_DAN | -0.1518 | 0.0007 | -0.0728 | 0.0562 |
| 69 | Ce_IL_FPN-Cb_C_FPN | -0.1625 | 0.0017 | -0.1196 | 0.0136 |
| 70 | Ce_IL_DMN-Cb_C_VN | 0.0941 | 0.0015 | 0.0670 | 0.0233 |
| 71 | Ce_IL_DMN-Cb_C_DAN | 0.1148 | 0.0018 | 0.0574 | 0.0813 |
| 72 | Ce_IL_VN-Cb_I_DAN | 0.1537 | 0.0005 | 0.0719 | 0.0530 |
| 73 | Ce_IL_SMN-Cb_I_VN | -0.1424 | 0.0001 | -0.1014 | 0.0034 |
| 74 | Ce_IL_SMN-Cb_I_SMN | -0.1579 | 0.0010 | -0.1066 | 0.0142 |
| 75 | Ce_IL_SMN-Cb_I_VAN | -0.1852 | 0.0008 | -0.1205 | 0.0115 |
| 76 | Ce_IL_VAN-Cb_I_VN | -0.1036 | 0.0015 | -0.0841 | 0.0070 |
| 77 | Ce_IL_VAN-Cb_I_DAN | -0.1849 | 0.0002 | -0.1668 | 0.0001 |
| 78 | Ce_IL_VAN-Cb_I_VAN | -0.2240 | 0.0001 | -0.1386 | 0.0061 |
| 79 | Ce_IL_VAN-Cb_I_LN | -0.1330 | 0.0003 | -0.0682 | 0.0352 |
| 80 | Ce_IL_LN-Cb_I_SMN | 0.1498 | 0.0010 | 0.0822 | 0.0480 |
| 81 | Ce_IL_LN-Cb_I_DAN | 0.1438 | 0.0002 | 0.1002 | 0.0100 |
| 82 | Ce_IL_DMN-Cb_I_DAN | 0.1275 | 0.0016 | 0.0857 | 0.0283 |
| 83 | Ce_IL_DMN-Cb_I_VAN | 0.1365 | 0.0018 | 0.0547 | 0.1173 |
| 84 | Ce_CL_VN-Cb_C_DAN | 0.1719 | 0.0004 | 0.0990 | 0.0152 |
| 85 | Ce_CL_SMN-Cb_C_VN | -0.1081 | 0.0017 | -0.0542 | 0.0641 |
| 86 | Ce_CL_SMN-Cb_C_FPN | 0.1416 | 0.0018 | 0.0890 | 0.0323 |
| 87 | Ce_CL_VAN-Cb_C_DAN | -0.1446 | 0.0017 | -0.1377 | 0.0022 |
| 88 | Ce_CL_VAN-Cb_C_VAN | -0.1957 | 0.0002 | -0.1053 | 0.0264 |
| 89 | Ce_CL_LN-Cb_C_SMN | 0.1555 | 0.0008 | 0.0757 | 0.0587 |
| 90 | Ce_CL_DMN-Cb_C_DAN | 0.1639 | 0.0004 | 0.1009 | 0.0113 |
| 91 | Ce_CL_DMN-Cb_C_VAN | 0.1938 | 0.0001 | 0.1051 | 0.0158 |
| 92 | Ce_CL_DAN-Cb_I_DMN | 0.1807 | 0.0001 | 0.1007 | 0.0114 |
| 93 | Ce_CL_VAN-Cb_I_DAN | -0.1364 | 0.0018 | -0.0983 | 0.0148 |
| 94 | Ce_CL_VAN-Cb_I_DMN | 0.1408 | 0.0017 | 0.0796 | 0.0423 |
| 95 | Ce_CL_LN-Cb_I_DAN | 0.1656 | 0.0001 | 0.0764 | 0.0418 |
| 96 | Ce_CL_LN-Cb_I_VAN | 0.1680 | 0.0006 | 0.0740 | 0.0624 |
| 97 | Ce_CL_FPN-Cb_I_DMN | 0.1430 | 0.0005 | 0.1000 | 0.0079 |
| 98 | Ce_CL_DMN-Cb_I_VN | 0.0921 | 0.0018 | 0.0635 | 0.0211 |
| 99 | Ce_CL_DMN-Cb_I_DAN | 0.1529 | 0.0003 | 0.0657 | 0.0557 |
| 100 | Ce_CL_DMN-Cb_I_VAN | 0.1581 | 0.0001 | 0.1084 | 0.0051 |
| **17 modules** | |  |  |  |  |
| 1 | Ce_IL_VN_A-Ce_IL_DAN_A | 0.1995 | 0.0001 | 0.1154 | 0.0113 |
| 2 | Ce_IL_VN_A-Ce_IL_DAN_B | 0.1566 | 0.0008 | 0.0925 | 0.0274 |
| 3 | Ce_IL_VN_A-Ce_IL_LN_B | 0.1423 | 0.0005 | 0.0732 | 0.0336 |
| 4 | Ce_IL_VN_A-Ce_IL_DMN_C | 0.1826 | 0.0007 | 0.1063 | 0.0216 |
| 5 | Ce_IL_VN_B-Ce_IL_DAN_A | 0.1460 | 0.0005 | 0.0834 | 0.0389 |
| 6 | Ce_IL_VN_B-Ce_IL_DMN_C | 0.1839 | 0.0005 | 0.1140 | 0.0228 |
| 7 | Ce_IL_SMN_A-Ce_IL_DAN_B | 0.1813 | 0.0003 | 0.1134 | 0.0117 |
| 8 | Ce_IL_SMN_A-Ce_IL_FPN_B | 0.1424 | 0.0008 | 0.0996 | 0.0135 |
| 9 | Ce_IL_SMN_B-Ce_IL_TPN | 0.2544 | 0.0001 | 0.1928 | 0.0004 |
| 10 | Ce_IL_DAN_A-Ce_IL_TPN | 0.1622 | 0.0007 | 0.1081 | 0.0113 |
| 11 | Ce_IL_VAN_A-Ce_IL_TPN | 0.2432 | 0.0002 | 0.1687 | 0.0062 |
| 12 | Ce_IL_VAN_A-Ce_IL_DMN_A | 0.1896 | 0.0003 | 0.1195 | 0.0139 |
| 13 | Ce_IL_VAN_A-Ce_IL_DMN_B | 0.1745 | 0.0001 | 0.1168 | 0.0084 |
| 14 | Ce_IL_VAN_B-Ce_IL_DMN_B | 0.1841 | 0.0003 | 0.1205 | 0.0086 |
| 15 | Ce_IL_FPN_A-Ce_IL_DMN_A | 0.1696 | 0.0003 | 0.1230 | 0.0032 |
| 16 | Ce_IL_DMN_C-Ce_IL_DMN_A | -0.2568 | 0.0002 | -0.1952 | 0.0011 |
| 17 | Ce_CL_VN_A-Ce_CL_DAN_A | 0.2238 | 0.0001 | 0.1325 | 0.0045 |
| 18 | Ce_CL_VN_A-Ce_CL_DAN_B | 0.1540 | 0.0005 | 0.0789 | 0.0349 |
| 19 | Ce_CL_VN_A-Ce_CL_VAN_B | 0.1508 | 0.0008 | 0.0443 | 0.1555 |
| 20 | Ce_CL_VN_A-Ce_CL_FPN_A | 0.2073 | 0.0002 | 0.0986 | 0.0117 |
| 21 | Ce_CL_VN_B-Ce_CL_DAN_A | 0.1472 | 0.0005 | 0.1246 | 0.0025 |
| 22 | Ce_CL_VN_B-Ce_CL_VAN_B | 0.1994 | 0.0001 | 0.1055 | 0.0061 |
| 23 | Ce_CL_VN_B-Ce_CL_FPN_A | 0.1853 | 0.0002 | 0.1179 | 0.0031 |
| 24 | Ce_CL_VAN_B-Ce_CL_DMN_A | 0.2013 | 0.0003 | 0.1356 | 0.0054 |
| 25 | Ce_CL_VAN_B-Ce_CL_DMN_B | 0.1865 | 0.0001 | 0.1046 | 0.0162 |
| 26 | Ce_CL_FPN_A-Ce_CL_DMN_A | 0.1713 | 0.0002 | 0.1309 | 0.0039 |
| 27 | Ce_IL_VN_A-Ce_CL_VN_B | -0.1494 | 0.0008 | -0.0724 | 0.0595 |
| 28 | Ce_IL_SMN_A-Ce_CL_SMN_A | -0.2797 | 0.0001 | -0.1406 | 0.0204 |
| 29 | Ce_IL_SMN_A-Ce_CL_VAN_A | -0.1837 | 0.0004 | -0.1510 | 0.0028 |
| 30 | Ce_IL_SMN_B-Ce_CL_SMN_A | -0.2016 | 0.0005 | -0.1236 | 0.0279 |
| 31 | Ce_IL_SMN_B-Ce_CL_DAN_B | -0.1865 | 0.0002 | -0.1431 | 0.0051 |
| 32 | Ce_IL_DAN_A-Ce_CL_VN_A | 0.2156 | 0.0001 | 0.1219 | 0.0070 |
| 33 | Ce_IL_DAN_A-Ce_CL_FPN_A | -0.1958 | 0.0001 | -0.1336 | 0.0025 |
| 34 | Ce_IL_DAN_B-Ce_CL_VN_A | 0.2012 | 0.0001 | 0.0987 | 0.0182 |
| 35 | Ce_IL_DAN_B-Ce_CL_DAN_B | -0.1998 | 0.0002 | -0.1012 | 0.0385 |
| 36 | Ce_IL_DAN_B-Ce_CL_VAN_B | -0.1867 | 0.0002 | -0.1429 | 0.0008 |
| 37 | Ce_IL_DAN_B-Ce_CL_FPN_A | -0.1925 | 0.0001 | -0.1179 | 0.0057 |
| 38 | Ce_IL_VAN_A-Ce_CL_DAN_B | -0.2868 | 0.0001 | -0.2188 | 0.0003 |
| 39 | Ce_IL_VAN_A-Ce_CL_VAN_A | -0.3529 | 0.0001 | -0.2430 | 0.0005 |
| 40 | Ce_IL_VAN_A-Ce_CL_VAN_B | -0.2644 | 0.0001 | -0.1854 | 0.0002 |
| 41 | Ce_IL_VAN_A-Ce_CL_FPN_A | -0.1798 | 0.0001 | -0.1310 | 0.0021 |
| 42 | Ce_IL_FPN_C-Ce_CL_FPN_C | -0.2060 | 0.0001 | -0.1322 | 0.0044 |
| 43 | Ce_IL_FPN_C-Ce_CL_TPN | -0.2133 | 0.0001 | -0.1374 | 0.0010 |
| 44 | Ce_IL_FPN_A-Ce_CL_FPN_A | -0.1661 | 0.0003 | -0.1194 | 0.0062 |
| 45 | Ce_IL_TPN-Ce_CL_TPN | -0.1975 | 0.0002 | -0.1400 | 0.0055 |
| 46 | Ce_IL_TPN-Ce_CL_DMN_B | -0.2151 | 0.0001 | -0.1436 | 0.0023 |
| 47 | Ce_IL_DMN_C-Ce_CL_VN_A | 0.1492 | 0.0008 | 0.0861 | 0.0381 |
| 48 | Ce_IL_DMN_C-Ce_CL_DMN_C | -0.2588 | 0.0001 | -0.1351 | 0.0115 |
| 49 | Ce_IL_DMN_C-Ce_CL_DMN_A | -0.2399 | 0.0001 | -0.1590 | 0.0011 |
| 50 | Ce_IL_DMN_A-Ce_CL_DAN_B | 0.1802 | 0.0002 | 0.1032 | 0.0121 |
| 51 | Ce_IL_DMN_A-Ce_CL_VAN_B | 0.1932 | 0.0002 | 0.1341 | 0.0065 |
| 52 | Ce_IL_DMN_A-Ce_CL_FPN_A | 0.1727 | 0.0001 | 0.1352 | 0.0018 |
| 53 | Ce_IL_DMN_A-Ce_CL_DMN_B | -0.1701 | 0.0001 | -0.1553 | 0.0016 |
| 54 | Ce_IL_DMN_B-Ce_CL_DAN_B | 0.1599 | 0.0006 | 0.0846 | 0.0359 |
| 55 | Ce_IL_DMN_B-Ce_CL_VAN_B | 0.1871 | 0.0001 | 0.1164 | 0.0059 |
| 56 | Cb_IL_VN_B-Cb_IL_DAN_B | 0.1305 | 0.0002 | 0.0708 | 0.0201 |
| 57 | Cb_IL_VN_B-Cb_IL_FPN_A | 0.1275 | 0.0007 | 0.0482 | 0.1006 |
| 58 | Cb_IL_DAN_B-Cb_IL_LN_A | 0.1516 | 0.0003 | 0.0605 | 0.0880 |
| 59 | Cb_IL_DAN_B-Cb_IL_DMN_A | 0.2294 | 0.0001 | 0.1210 | 0.0064 |
| 60 | Cb_IL_VAN_A-Cb_IL_LN_A | 0.1665 | 0.0003 | 0.0623 | 0.0921 |
| 61 | Cb_IL_VAN_A-Cb_IL_FPN_B | 0.2173 | 0.0002 | 0.1028 | 0.0365 |
| 62 | Cb_IL_VAN_A-Cb_IL_DMN_A | 0.2287 | 0.0001 | 0.1128 | 0.0226 |
| 63 | Cb_IL_VAN_B-Cb_IL_FPN_B | 0.2438 | 0.0001 | 0.1289 | 0.0067 |
| 64 | Cb_IL_VAN_B-Cb_IL_DMN_A | 0.1836 | 0.0006 | 0.1167 | 0.0138 |
| 65 | Cb_IL_VAN_B-Cb_IL_DMN_B | 0.2401 | 0.0001 | 0.1486 | 0.0039 |
| 66 | Cb_IL_LN_A-Cb_IL_FPN_A | 0.1904 | 0.0002 | 0.0836 | 0.0322 |
| 67 | Cb_IL_LN_A-Cb_IL_FPN_B | 0.1793 | 0.0001 | 0.0862 | 0.0201 |
| 68 | Cb_IL_FPN_A-Cb_IL_FPN_B | 0.1899 | 0.0001 | 0.1374 | 0.0041 |
| 69 | Cb_IL_FPN_A-Cb_IL_DMN_A | 0.2024 | 0.0001 | 0.1607 | 0.0010 |
| 70 | Cb_IL_FPN_A-Cb_IL_DMN_B | 0.2375 | 0.0001 | 0.1682 | 0.0021 |
| 71 | Cb_CL_VN_B-Cb_CL_LN_A | 0.1196 | 0.0007 | 0.0537 | 0.0611 |
| 72 | Cb_CL_VN_B-Cb_CL_FPN_B | 0.1226 | 0.0005 | 0.0667 | 0.0286 |
| 73 | Cb_CL_VN_B-Cb_CL_DMN_A | 0.1380 | 0.0004 | 0.0934 | 0.0089 |
| 74 | Cb_CL_VN_B-Cb_CL_DMN_B | 0.1251 | 0.0004 | 0.0654 | 0.0300 |
| 75 | Cb_CL_SMN_A-Cb_CL_FPN_B | 0.1661 | 0.0001 | 0.0584 | 0.0935 |
| 76 | Cb_CL_SMN_A-Cb_CL_DMN_A | 0.1509 | 0.0003 | 0.0836 | 0.0314 |
| 77 | Cb_CL_SMN_B-Cb_CL_FPN_B | 0.2170 | 0.0001 | 0.0992 | 0.0136 |
| 78 | Cb_CL_SMN_B-Cb_CL_DMN_A | 0.2038 | 0.0001 | 0.1207 | 0.0042 |
| 79 | Cb_CL_SMN_B-Cb_CL_DMN_B | 0.1702 | 0.0001 | 0.0958 | 0.0097 |
| 80 | Cb_CL_DAN_A-Cb_CL_DMN_A | 0.1504 | 0.0005 | 0.0872 | 0.0308 |
| 81 | Cb_CL_DAN_A-Cb_CL_DMN_B | 0.1576 | 0.0002 | 0.0856 | 0.0263 |
| 82 | Cb_CL_DAN_B-Cb_CL_FPN_B | 0.1983 | 0.0001 | 0.0852 | 0.0337 |
| 83 | Cb_CL_DAN_B-Cb_CL_DMN_A | 0.2431 | 0.0001 | 0.1273 | 0.0045 |
| 84 | Cb_CL_DAN_B-Cb_CL_DMN_B | 0.1953 | 0.0001 | 0.1016 | 0.0169 |
| 85 | Cb_CL_VAN_A-Cb_CL_LN_A | 0.1802 | 0.0001 | 0.1109 | 0.0116 |
| 86 | Cb_CL_VAN_A-Cb_CL_FPN_A | 0.1548 | 0.0002 | 0.0520 | 0.1208 |
| 87 | Cb_CL_VAN_A-Cb_CL_FPN_B | 0.2789 | 0.0001 | 0.1348 | 0.0018 |
| 88 | Cb_CL_VAN_A-Cb_CL_TPN | -0.1055 | 0.0004 | -0.0580 | 0.0335 |
| 89 | Cb_CL_VAN_A-Cb_CL_DMN_A | 0.2567 | 0.0001 | 0.1656 | 0.0014 |
| 90 | Cb_CL_VAN_A-Cb_CL_DMN_B | 0.2339 | 0.0001 | 0.1415 | 0.0009 |
| 91 | Cb_CL_LN_A-Cb_CL_FPN_A | 0.1869 | 0.0001 | 0.1158 | 0.0035 |
| 92 | Cb_CL_LN_A-Cb_CL_FPN_B | 0.2427 | 0.0001 | 0.1212 | 0.0046 |
| 93 | Cb_CL_LN_A-Cb_CL_DMN_A | 0.2099 | 0.0002 | 0.1126 | 0.0114 |
| 94 | Cb_CL_LN_A-Cb_CL_DMN_B | 0.1975 | 0.0001 | 0.1020 | 0.0084 |
| 95 | Cb_IL_VN_B-Cb_CL_DAN_B | 0.1109 | 0.0007 | 0.0658 | 0.0260 |
| 96 | Cb_IL_SMN_A-Cb_CL_DMN_B | 0.1288 | 0.0006 | 0.0670 | 0.0344 |
| 97 | Cb_IL_SMN_B-Cb_CL_FPN_B | 0.1621 | 0.0004 | 0.0847 | 0.0298 |
| 98 | Cb_IL_SMN_B-Cb_CL_DMN_B | 0.1581 | 0.0001 | 0.0883 | 0.0146 |
| 99 | Cb_IL_DAN_B-Cb_CL_FPN_B | 0.1647 | 0.0002 | 0.0804 | 0.0425 |
| 100 | Cb_IL_DAN_B-Cb_CL_DMN_A | 0.2106 | 0.0001 | 0.1363 | 0.0028 |
| 101 | Cb_IL_DAN_B-Cb_CL_DMN_B | 0.1865 | 0.0001 | 0.1055 | 0.0093 |
| 102 | Cb_IL_VAN_A-Cb_CL_FPN_B | 0.2469 | 0.0001 | 0.1421 | 0.0036 |
| 103 | Cb_IL_VAN_A-Cb_CL_TPN | -0.1215 | 0.0001 | -0.0507 | 0.0525 |
| 104 | Cb_IL_VAN_A-Cb_CL_DMN_A | 0.2571 | 0.0001 | 0.1585 | 0.0043 |
| 105 | Cb_IL_VAN_A-Cb_CL_DMN_B | 0.2586 | 0.0001 | 0.1552 | 0.0018 |
| 106 | Cb_IL_VAN_B-Cb_CL_FPN_B | 0.1802 | 0.0002 | 0.1002 | 0.0218 |
| 107 | Cb_IL_VAN_B-Cb_CL_TPN | -0.1236 | 0.0005 | -0.0653 | 0.0274 |
| 108 | Cb_IL_VAN_B-Cb_CL_DMN_A | 0.1728 | 0.0007 | 0.1328 | 0.0083 |
| 109 | Cb_IL_VAN_B-Cb_CL_DMN_B | 0.2400 | 0.0001 | 0.1498 | 0.0023 |
| 110 | Cb_IL_LN_A-Cb_CL_DAN_B | 0.1658 | 0.0001 | 0.0952 | 0.0094 |
| 111 | Cb_IL_LN_A-Cb_CL_VAN_A | 0.1835 | 0.0001 | 0.1080 | 0.0113 |
| 112 | Cb_IL_LN_A-Cb_CL_FPN_A | 0.1883 | 0.0001 | 0.1161 | 0.0027 |
| 113 | Cb_IL_LN_A-Cb_CL_FPN_B | 0.2208 | 0.0001 | 0.1212 | 0.0027 |
| 114 | Cb_IL_LN_A-Cb_CL_DMN_A | 0.1809 | 0.0001 | 0.1128 | 0.0093 |
| 115 | Cb_IL_LN_A-Cb_CL_DMN_B | 0.2020 | 0.0001 | 0.1190 | 0.0012 |
| 116 | Cb_IL_FPN_A-Cb_CL_DMN_B | 0.1816 | 0.0003 | 0.1569 | 0.0019 |
| 117 | Cb_IL_FPN_B-Cb_CL_SMN_B | 0.1753 | 0.0001 | 0.1249 | 0.0012 |
| 118 | Cb_IL_FPN_B-Cb_CL_VAN_A | 0.1717 | 0.0004 | 0.1212 | 0.0051 |
| 119 | Cb_IL_TPN-Cb_CL_VN_B | 0.1199 | 0.0001 | 0.0695 | 0.0161 |
| 120 | Cb_IL_DMN_A-Cb_CL_VN_B | 0.1519 | 0.0001 | 0.0945 | 0.0071 |
| 121 | Cb_IL_DMN_A-Cb_CL_SMN_B | 0.1352 | 0.0008 | 0.0983 | 0.0120 |
| 122 | Cb_IL_DMN_A-Cb_CL_DAN_A | 0.1498 | 0.0001 | 0.0990 | 0.0037 |
| 123 | Cb_IL_DMN_A-Cb_CL_DAN_B | 0.2288 | 0.0001 | 0.1399 | 0.0026 |
| 124 | Cb_IL_DMN_A-Cb_CL_VAN_A | 0.2346 | 0.0001 | 0.1643 | 0.0002 |
| 125 | Cb_IL_DMN_A-Cb_CL_FPN_A | 0.1821 | 0.0001 | 0.1517 | 0.0005 |
| 126 | Ce_IL_SMN_B-Cb_C_VN_B | -0.1240 | 0.0006 | -0.0928 | 0.0083 |
| 127 | Ce_IL_SMN_B-Cb_C_SMN_B | -0.1802 | 0.0003 | -0.1060 | 0.0135 |
| 128 | Ce_IL_SMN_B-Cb_C_VAN_A | -0.1764 | 0.0004 | -0.1127 | 0.0105 |
| 129 | Ce_IL_DAN_B-Cb_C_DAN_B | -0.1435 | 0.0008 | -0.0970 | 0.0205 |
| 130 | Ce_IL_VAN_A-Cb_C_SMN_A | -0.1536 | 0.0001 | -0.0980 | 0.0110 |
| 131 | Ce_IL_VAN_A-Cb_C_SMN_B | -0.1715 | 0.0002 | -0.1191 | 0.0028 |
| 132 | Ce_IL_VAN_A-Cb_C_DAN_A | -0.1295 | 0.0002 | -0.1009 | 0.0038 |
| 133 | Ce_IL_VAN_A-Cb_C_DAN_B | -0.1742 | 0.0002 | -0.1368 | 0.0008 |
| 134 | Ce_IL_VAN_A-Cb_C_VAN_A | -0.1919 | 0.0001 | -0.1449 | 0.0005 |
| 135 | Ce_IL_LN_A-Cb_C_DAN_B | 0.1281 | 0.0007 | 0.0509 | 0.0984 |
| 136 | Ce_IL_LN_B-Cb_C_SMN_A | 0.1468 | 0.0002 | 0.0899 | 0.0077 |
| 137 | Ce_IL_FPN_A-Cb_C_FPN_A | -0.2602 | 0.0001 | -0.1848 | 0.0004 |
| 138 | Ce_IL_DMN_C-Cb_C_VAN_A | 0.1364 | 0.0004 | 0.0740 | 0.0288 |
| 139 | Ce_IL_VN_A-Cb_I_FPN_C | 0.1313 | 0.0004 | 0.0494 | 0.0948 |
| 140 | Ce_IL_VN_B-Cb_I_DAN_B | 0.1401 | 0.0005 | 0.0827 | 0.0210 |
| 141 | Ce_IL_SMN_B-Cb_I_VAN_A | -0.1649 | 0.0004 | -0.0955 | 0.0269 |
| 142 | Ce_IL_SMN_B-Cb_I_LN_A | -0.1493 | 0.0002 | -0.0975 | 0.0128 |
| 143 | Ce_IL_VAN_A-Cb_I_DAN_B | -0.1921 | 0.0002 | -0.1694 | 0.0006 |
| 144 | Ce_IL_VAN_A-Cb_I_VAN_A | -0.1986 | 0.0002 | -0.1267 | 0.0097 |
| 145 | Ce_IL_VAN_A-Cb_I_VAN_B | -0.1959 | 0.0001 | -0.1097 | 0.0182 |
| 146 | Ce_IL_VAN_A-Cb_I_LN_A | -0.1384 | 0.0006 | -0.0816 | 0.0218 |
| 147 | Ce_IL_VAN_B-Cb_I_VAN_B | -0.2239 | 0.0001 | -0.1301 | 0.0039 |
| 148 | Ce_IL_LN_A-Cb_I_DAN_B | 0.1284 | 0.0006 | 0.0780 | 0.0330 |
| 149 | Ce_IL_LN_B-Cb_I_DAN_B | 0.1429 | 0.0004 | 0.1005 | 0.0058 |
| 150 | Ce_IL_FPN_A-Cb_I_DAN_B | -0.1260 | 0.0006 | -0.0718 | 0.0260 |
| 151 | Ce_IL_FPN_A-Cb_I_FPN_A | -0.1763 | 0.0001 | -0.1182 | 0.0036 |
| 152 | Ce_IL_DMN_C-Cb_I_SMN_A | 0.1450 | 0.0004 | 0.0562 | 0.0919 |
| 153 | Ce_IL_DMN_C-Cb_I_DAN_B | 0.1329 | 0.0003 | 0.0933 | 0.0085 |
| 154 | Ce_IL_DMN_B-Cb_I_DAN_B | 0.1373 | 0.0007 | 0.0795 | 0.0242 |
| 155 | Ce_CL_VN_A-Cb_C_FPN_C | 0.1493 | 0.0001 | 0.0794 | 0.0208 |
| 156 | Ce_CL_LN_B-Cb_C_DAN_B | 0.1373 | 0.0003 | 0.0868 | 0.0139 |
| 157 | Ce_CL_FPN_C-Cb_C_FPN_A | 0.1371 | 0.0006 | 0.1079 | 0.0066 |
| 158 | Ce_CL_FPN_A-Cb_C_DAN_B | -0.1559 | 0.0006 | -0.0864 | 0.0262 |
| 159 | Ce_CL_DMN_C-Cb_C_FPN_A | 0.1344 | 0.0006 | 0.1082 | 0.0066 |
| 160 | Ce_CL_DMN_A-Cb_C_FPN_A | 0.1933 | 0.0001 | 0.1457 | 0.0025 |
| 161 | Ce_CL_DMN_A-Cb_C_DAN_B | 0.1418 | 0.0003 | 0.0932 | 0.0119 |
| 162 | Ce_CL_DMN_A-Cb_C_VAN_B | 0.1603 | 0.0007 | 0.1337 | 0.0021 |
| 163 | Ce_CL_DMN_B-Cb_C_FPN_A | 0.1973 | 0.0001 | 0.1411 | 0.0017 |
| 164 | Ce_CL_DMN_B-Cb_C_DAN_B | 0.1637 | 0.0005 | 0.0794 | 0.0420 |
| 165 | Ce_CL_DMN_B-Cb_C_VAN_A | 0.1717 | 0.0005 | 0.0881 | 0.0313 |
| 166 | Ce_CL_DMN_B-Cb_C_VAN_B | 0.1701 | 0.0001 | 0.1142 | 0.0057 |
| 167 | Ce_CL_VN_B-Cb_C_FPN_A | 0.1776 | 0.0005 | 0.1224 | 0.0074 |
| 168 | Ce_CL_VN_B-Cb_C_DAN_B | 0.1428 | 0.0004 | 0.1109 | 0.0068 |
| 169 | Ce_CL_SMN_A-Cb_C_FPN_B | 0.1456 | 0.0006 | 0.0900 | 0.0236 |
| 170 | Ce_CL_VAN_A-Cb_C_DAN_B | -0.1541 | 0.0008 | -0.1434 | 0.0009 |
| 171 | Ce_CL_VAN_B-Cb_C_DMN_A | 0.1439 | 0.0005 | 0.0906 | 0.0216 |
| 172 | Ce_CL_LN_A-Cb_C_DAN_B | 0.1551 | 0.0006 | 0.0832 | 0.0429 |
| 173 | Ce_CL_SMN_A-Cb_I_FPN_B | 0.1768 | 0.0001 | 0.0890 | 0.0213 |
| 174 | Ce_CL_SMN_A-Cb_I_DMN_B | 0.1519 | 0.0001 | 0.0589 | 0.0680 |
| 175 | Ce_CL_DAN_B-Cb_I_FPN_B | 0.1647 | 0.0001 | 0.0798 | 0.0345 |
| 176 | Ce_CL_DAN_B-Cb_I_DMN_A | 0.1770 | 0.0002 | 0.0879 | 0.0290 |
| 177 | Ce_CL_DAN_B-Cb_I_DMN_B | 0.1717 | 0.0001 | 0.0957 | 0.0151 |
| 178 | Ce_CL_VAN_A-Cb_I_SMN_B | -0.1369 | 0.0008 | -0.0918 | 0.0192 |
| 179 | Ce_CL_VAN_A-Cb_I_DAN_A | -0.1267 | 0.0007 | -0.0798 | 0.0206 |
| 180 | Ce_CL_VAN_A-Cb_I_VAN_A | -0.1597 | 0.0004 | -0.1166 | 0.0053 |
| 181 | Ce_CL_VAN_B-Cb_I_FPN_B | 0.1654 | 0.0002 | 0.0933 | 0.0183 |
| 182 | Ce_CL_VAN_B-Cb_I_DMN_B | 0.1791 | 0.0001 | 0.1148 | 0.0076 |
| 183 | Ce_CL_LN_A-Cb_I_DAN_B | 0.1533 | 0.0004 | 0.0756 | 0.0328 |
| 184 | Ce_CL_FPN_C-Cb_I_VAN_A | 0.1321 | 0.0004 | 0.0797 | 0.0166 |
| 185 | Ce_CL_FPN_C-Cb_I_FPN_A | 0.2070 | 0.0002 | 0.1265 | 0.0034 |
| 186 | Ce_CL_FPN_C-Cb_I_FPN_B | 0.1679 | 0.0002 | 0.0936 | 0.0164 |
| 187 | Ce_CL_FPN_C-Cb_I_DMN_B | 0.1777 | 0.0002 | 0.0918 | 0.0248 |
| 188 | Ce_CL_FPN_A-Cb_I_DAN_B | -0.1408 | 0.0004 | -0.0807 | 0.0252 |
| 189 | Ce_CL_DMN_A-Cb_I_VAN_A | 0.1361 | 0.0005 | 0.1075 | 0.0034 |
| 190 | Ce_CL_DMN_A-Cb_I_FPN_A | 0.1670 | 0.0003 | 0.1139 | 0.0091 |
| 191 | Ce_CL_DMN_A-Cb_I_TPN | 0.0995 | 0.0004 | 0.0581 | 0.0311 |
| 192 | Ce_CL_DMN_B-Cb_I_VN_B | 0.1111 | 0.0005 | 0.0635 | 0.0194 |
| 193 | Ce_CL_DMN_B-Cb_I_FPN_A | 0.1403 | 0.0005 | 0.0894 | 0.0186 |

† Model adjusted for age and sex.

‡ Model adjusted for age, sex, MMSE, and education level.

Δ, Delta (Stroke - HC); Stroke, stroke patients; HC, healthy controls; Ce, cerebrum; Cb, cerebellum; IL, ipsilesional; CL, contralesional; I, ipsilateral; C, contralateral; VN, visual network; SMN, somatomotor network; DAN, dorsal attention network; VAN, ventral attention network; LN, limbic network; FPN, frontoparietal network; TPN, temporal parietal network; DMN, default mode network.

**Table S2. The summary of significant between-group differences in module-level functional connectivity.**

| **Region** | **Stroke-HC** | **Intra-hemispheric connections** | | **Inter-hemispheric connections** | | **Total** |
| --- | --- | --- | --- | --- | --- | --- |
|  |  | **IL** | **CL** |  |  |  |
| **7 modules** |  |  |  |  | | 100 |
| cerebrum | decreased | 0 | 0 | 6 | | 20 |
|  | increased | 6 | 4 | 4 | |  |
| cerebellum | decreased | 0 | 0 | 0 | | 40 |
|  | increased | 11 | 12 | 17 | |  |
|  |  | **IL→I** | **CL→I** | **IL→C** | **CL→C** |  |
| cerebro-cerebellum | decreased | 7 | 1 | 9 | 3 | 40 |
|  | increased | 5 | 8 | 2 | 5 |  |
| **17 modules** |  |  |  |  |  | 193 |
| cerebrum | decreased | 1 | 0 | 21 | | 55 |
|  | increased | 15 | 10 | 8 | |  |
| cerebellum | decreased | 0 | 1 | 2 | | 70 |
|  | increased | 15 | 23 | 29 | |  |
|  |  | **IL→I** | **CL→I** | **IL→C** | **CL→C** |  |
| cerebro-cerebellum | decreased | 9 | 4 | 10 | 2 | 68 |
|  | increased | 7 | 17 | 3 | 16 |  |

Stroke, stroke patients; HC, healthy controls; IL, ipsilesional hemisphere; CL, contralesional hemisphere; I, ipsilateral; C, contralateral; IL→I, ipsilesional cerebrum to ipsilateral cerebellum; CL→I, contralesional cerebrum to ipsilateral cerebellum; IL→C, ipsilesional cerebrum to contralateral cerebellum; CL→C, contralesional cerebrum to contralateral cerebellum.

**Table S3. Frequency of stroke-related functional connectivity changes between key networks across brain regions.**

| **Region** | **networks** | **7 modules** | | **17 modules** | |
| --- | --- | --- | --- | --- | --- |
| cerebrum | DAN | 10 | | 19 | |
|  | DMN | 7 | | 24 | |
| cerebellum | LN | 17 | | - | |
|  | FPN | - | | 27 | |
|  | DMN | 18 | | 36 | |
|  |  | increased | decreased | increased | decreased |
| ipsilesional cerebrum to bilateral cerebellum | cerebral SMN | - | 6 | - | - |
|  | cerebral VAN | - | 7 | - | 10 |
|  | cerebral LN | - | - | 4 | - |
|  | cerebral DMN | 4 | - | 4 | - |
|  | cerebellar DAN | 4 | 4 | 6 | 5 |
|  | cerebellar VAN | - | 4 | - | 6 |
| contralesional cerebrum to bilateral cerebellum | cerebral VAN | - | 3 | - | 4 |
|  | cerebral DMN | 5 | - | 13 | - |
|  | cerebellar DAN | 4 | 2 | - | 4 |
|  | cerebellar FPN | - | - | 14 | - |

SMN, somatomotor network; DAN, dorsal attention network; VAN, ventral attention network; LN, limbic network; FPN, frontoparietal network; DMN, default mode network.

**Table S4. Subgroup analyses by lesion laterality: between-group differences relative to healthy controls.**

|  |  | **Left lesion-HC** | | **Right lesion-HC** | |
| --- | --- | --- | --- | --- | --- |
|  | **connections** | **Δ** | ***p*** | **Δ** | ***p*** |
| **7 modules** | |  |  |  |  |
| 1 | Ce_IL_VN-Ce_IL_DAN | 0.2518 | 0.0001 | 0.1419 | 0.0076 |
| 2 | Ce_IL_SMN-Ce_IL_DAN | 0.1455 | 0.0033 | 0.1854 | 0.0022 |
| 3 | Ce_IL_SMN-Ce_IL_LN | 0.1622 | 0.0002 | 0.1048 | 0.0123 |
| 4 | Ce_IL_DAN-Ce_IL_DMN | 0.2152 | 0.0003 | 0.0639 | 0.1283 |
| 5 | Ce_IL_VAN-Ce_IL_DMN | 0.1388 | 0.0223 | 0.2517 | 0.0002 |
| 6 | Ce_IL_FPN-Ce_IL_DMN | 0.2387 | 0.0002 | 0.1109 | 0.0208 |
| 7 | Ce_CL_VN-Ce_CL_DAN | 0.2535 | 0.0001 | 0.1973 | 0.0001 |
| 8 | Ce_CL_VN-Ce_CL_VAN | 0.1662 | 0.0018 | 0.1287 | 0.0096 |
| 9 | Ce_CL_VN-Ce_CL_FPN | 0.2385 | 0.0001 | 0.2299 | 0.0001 |
| 10 | Ce_CL_FPN-Ce_CL_DMN | 0.1781 | 0.0013 | 0.2382 | 0.0001 |
| 11 | Ce_IL_SMN-Ce_CL_SMN | -0.3763 | 0.0001 | -0.1724 | 0.0234 |
| 12 | Ce_IL_DAN-Ce_CL_VN | 0.2438 | 0.0002 | 0.1551 | 0.0063 |
| 13 | Ce_IL_DAN-Ce_CL_DAN | -0.1923 | 0.0008 | -0.1916 | 0.0013 |
| 14 | Ce_IL_DAN-Ce_CL_FPN | -0.0463 | 0.1855 | -0.2333 | 0.0001 |
| 15 | Ce_IL_VAN-Ce_CL_DAN | -0.1222 | 0.0265 | -0.2809 | 0.0001 |
| 16 | Ce_IL_VAN-Ce_CL_VAN | -0.3598 | 0.0001 | -0.2752 | 0.0006 |
| 17 | Ce_IL_VAN-Ce_CL_FPN | -0.0960 | 0.0274 | -0.2980 | 0.0001 |
| 18 | Ce_IL_DMN-Ce_CL_DAN | 0.1140 | 0.0217 | 0.2687 | 0.0001 |
| 19 | Ce_IL_DMN-Ce_CL_VAN | 0.0828 | 0.0918 | 0.2835 | 0.0001 |
| 20 | Ce_IL_DMN-Ce_CL_FPN | 0.1309 | 0.0124 | 0.2055 | 0.0004 |
| 21 | Cb_IL_VN-Cb_IL_DAN | 0.1437 | 0.0002 | 0.0893 | 0.0302 |
| 22 | Cb_IL_VN-Cb_IL_FPN | 0.1730 | 0.0002 | 0.1135 | 0.0115 |
| 23 | Cb_IL_SMN-Cb_IL_LN | 0.1649 | 0.0042 | 0.1100 | 0.0604 |
| 24 | Cb_IL_DAN-Cb_IL_LN | 0.2157 | 0.0002 | 0.1463 | 0.0054 |
| 25 | Cb_IL_DAN-Cb_IL_DMN | 0.1792 | 0.0037 | 0.2059 | 0.0005 |
| 26 | Cb_IL_VAN-Cb_IL_LN | 0.1534 | 0.0043 | 0.1655 | 0.0052 |
| 27 | Cb_IL_VAN-Cb_IL_FPN | 0.2022 | 0.0027 | 0.2426 | 0.0004 |
| 28 | Cb_IL_VAN-Cb_IL_DMN | 0.1739 | 0.0069 | 0.3343 | 0.0001 |
| 29 | Cb_IL_LN-Cb_IL_FPN | 0.2086 | 0.0001 | 0.1435 | 0.0048 |
| 30 | Cb_IL_LN-Cb_IL_DMN | 0.1304 | 0.0017 | 0.0924 | 0.0342 |
| 31 | Cb_IL_FPN-Cb_IL_DMN | 0.1308 | 0.0292 | 0.2396 | 0.0007 |
| 32 | Cb_CL_VN-Cb_CL_LN | 0.1081 | 0.0035 | 0.1479 | 0.0001 |
| 33 | Cb_CL_VN-Cb_CL_DMN | 0.1575 | 0.0002 | 0.1061 | 0.0072 |
| 34 | Cb_CL_SMN-Cb_CL_DAN | 0.1397 | 0.0015 | 0.1111 | 0.0207 |
| 35 | Cb_CL_SMN-Cb_CL_FPN | 0.1735 | 0.0025 | 0.1800 | 0.0014 |
| 36 | Cb_CL_SMN-Cb_CL_DMN | 0.1809 | 0.0002 | 0.2385 | 0.0001 |
| 37 | Cb_CL_DAN-Cb_CL_LN | 0.1461 | 0.0019 | 0.0914 | 0.0393 |
| 38 | Cb_CL_DAN-Cb_CL_DMN | 0.2524 | 0.0001 | 0.1857 | 0.0016 |
| 39 | Cb_CL_VAN-Cb_CL_LN | 0.2029 | 0.0002 | 0.1633 | 0.0041 |
| 40 | Cb_CL_VAN-Cb_CL_FPN | 0.2013 | 0.0006 | 0.1937 | 0.0011 |
| 41 | Cb_CL_VAN-Cb_CL_DMN | 0.2389 | 0.0001 | 0.2250 | 0.0005 |
| 42 | Cb_CL_LN-Cb_CL_FPN | 0.2135 | 0.0004 | 0.1692 | 0.0003 |
| 43 | Cb_CL_LN-Cb_CL_DMN | 0.2422 | 0.0001 | 0.2121 | 0.0001 |
| 44 | Cb_IL_VN-Cb_CL_VN | 0.1577 | 0.0003 | 0.0830 | 0.0679 |
| 45 | Cb_IL_VN-Cb_CL_LN | 0.1238 | 0.0022 | 0.1833 | 0.0002 |
| 46 | Cb_IL_VN-Cb_CL_DMN | 0.1422 | 0.0005 | 0.1132 | 0.0063 |
| 47 | Cb_IL_SMN-Cb_CL_DMN | 0.1957 | 0.0003 | 0.1340 | 0.0043 |
| 48 | Cb_IL_DAN-Cb_CL_DMN | 0.2176 | 0.0005 | 0.2006 | 0.0004 |
| 49 | Cb_IL_VAN-Cb_CL_FPN | 0.1781 | 0.0048 | 0.2330 | 0.0002 |
| 50 | Cb_IL_VAN-Cb_CL_DMN | 0.2668 | 0.0003 | 0.3331 | 0.0001 |
| 51 | Cb_IL_LN-Cb_CL_VN | 0.1283 | 0.0028 | 0.0895 | 0.0126 |
| 52 | Cb_IL_LN-Cb_CL_DAN | 0.1979 | 0.0003 | 0.2002 | 0.0001 |
| 53 | Cb_IL_LN-Cb_CL_VAN | 0.1803 | 0.0018 | 0.1588 | 0.0031 |
| 54 | Cb_IL_LN-Cb_CL_FPN | 0.1988 | 0.0001 | 0.1757 | 0.0002 |
| 55 | Cb_IL_LN-Cb_CL_DMN | 0.2154 | 0.0001 | 0.2114 | 0.0001 |
| 56 | Cb_IL_FPN-Cb_CL_LN | 0.1484 | 0.0040 | 0.1116 | 0.0242 |
| 57 | Cb_IL_DMN-Cb_CL_VN | 0.1768 | 0.0003 | 0.0899 | 0.0276 |
| 58 | Cb_IL_DMN-Cb_CL_SMN | 0.0886 | 0.0277 | 0.1626 | 0.0009 |
| 59 | Cb_IL_DMN-Cb_CL_DAN | 0.2180 | 0.0006 | 0.1792 | 0.0029 |
| 60 | Cb_IL_DMN-Cb_CL_VAN | 0.1814 | 0.0014 | 0.1699 | 0.0031 |
| 61 | Ce_IL_SMN-Cb_C_VN | -0.1773 | 0.0003 | -0.0982 | 0.0190 |
| 62 | Ce_IL_SMN-Cb_C_SMN | -0.1440 | 0.0087 | -0.2193 | 0.0005 |
| 63 | Ce_IL_SMN-Cb_C_VAN | -0.0972 | 0.0364 | -0.1860 | 0.0025 |
| 64 | Ce_IL_DAN-Cb_C_DAN | -0.1789 | 0.0007 | -0.2241 | 0.0002 |
| 65 | Ce_IL_VAN-Cb_C_SMN | -0.1331 | 0.0067 | -0.2155 | 0.0002 |
| 66 | Ce_IL_VAN-Cb_C_DAN | -0.1981 | 0.0003 | -0.1983 | 0.0009 |
| 67 | Ce_IL_VAN-Cb_C_VAN | -0.1626 | 0.0008 | -0.1535 | 0.0081 |
| 68 | Ce_IL_FPN-Cb_C_DAN | -0.2042 | 0.0002 | -0.0858 | 0.0748 |
| 69 | Ce_IL_FPN-Cb_C_FPN | -0.1767 | 0.0041 | -0.1392 | 0.0182 |
| 70 | Ce_IL_DMN-Cb_C_VN | 0.1178 | 0.0012 | 0.0667 | 0.0460 |
| 71 | Ce_IL_DMN-Cb_C_DAN | 0.1230 | 0.0071 | 0.1027 | 0.0244 |
| 72 | Ce_IL_VN-Cb_I_DAN | 0.2328 | 0.0001 | 0.0518 | 0.1723 |
| 73 | Ce_IL_SMN-Cb_I_VN | -0.1443 | 0.0006 | -0.1362 | 0.0022 |
| 74 | Ce_IL_SMN-Cb_I_SMN | -0.1376 | 0.0097 | -0.1810 | 0.0006 |
| 75 | Ce_IL_SMN-Cb_I_VAN | -0.1180 | 0.0295 | -0.2669 | 0.0001 |
| 76 | Ce_IL_VAN-Cb_I_VN | -0.0984 | 0.0077 | -0.1113 | 0.0046 |
| 77 | Ce_IL_VAN-Cb_I_DAN | -0.1256 | 0.0098 | -0.2563 | 0.0001 |
| 78 | Ce_IL_VAN-Cb_I_VAN | -0.0818 | 0.0952 | -0.3994 | 0.0001 |
| 79 | Ce_IL_VAN-Cb_I_LN | -0.0957 | 0.0221 | -0.1780 | 0.0004 |
| 80 | Ce_IL_LN-Cb_I_SMN | 0.1029 | 0.0473 | 0.2105 | 0.0005 |
| 81 | Ce_IL_LN-Cb_I_DAN | 0.1452 | 0.0025 | 0.1436 | 0.0040 |
| 82 | Ce_IL_DMN-Cb_I_DAN | 0.1410 | 0.0054 | 0.1075 | 0.0285 |
| 83 | Ce_IL_DMN-Cb_I_VAN | 0.1264 | 0.0156 | 0.1465 | 0.0069 |
| 84 | Ce_CL_VN-Cb_C_DAN | 0.1970 | 0.0002 | 0.1354 | 0.0118 |
| 85 | Ce_CL_SMN-Cb_C_VN | -0.1183 | 0.0018 | -0.0928 | 0.0192 |
| 86 | Ce_CL_SMN-Cb_C_FPN | 0.1670 | 0.0017 | 0.1086 | 0.0287 |
| 87 | Ce_CL_VAN-Cb_C_DAN | -0.1042 | 0.0308 | -0.1941 | 0.0011 |
| 88 | Ce_CL_VAN-Cb_C_VAN | -0.0891 | 0.0748 | -0.3254 | 0.0001 |
| 89 | Ce_CL_LN-Cb_C_SMN | 0.1496 | 0.0086 | 0.1654 | 0.0026 |
| 90 | Ce_CL_DMN-Cb_C_DAN | 0.1705 | 0.0024 | 0.1560 | 0.0034 |
| 91 | Ce_CL_DMN-Cb_C_VAN | 0.1272 | 0.0150 | 0.2762 | 0.0001 |
| 92 | Ce_CL_DAN-Cb_I_DMN | 0.1358 | 0.0045 | 0.2331 | 0.0003 |
| 93 | Ce_CL_VAN-Cb_I_DAN | -0.1647 | 0.0004 | -0.1016 | 0.0462 |
| 94 | Ce_CL_VAN-Cb_I_DMN | 0.1189 | 0.0181 | 0.1652 | 0.0028 |
| 95 | Ce_CL_LN-Cb_I_DAN | 0.1608 | 0.0025 | 0.1707 | 0.0009 |
| 96 | Ce_CL_LN-Cb_I_VAN | 0.1780 | 0.0008 | 0.1588 | 0.0019 |
| 97 | Ce_CL_FPN-Cb_I_DMN | 0.1042 | 0.0200 | 0.1919 | 0.0001 |
| 98 | Ce_CL_DMN-Cb_I_VN | 0.0773 | 0.0226 | 0.1121 | 0.0019 |
| 99 | Ce_CL_DMN-Cb_I_DAN | 0.1608 | 0.0017 | 0.1435 | 0.0044 |
| 100 | Ce_CL_DMN-Cb_I_VAN | 0.1420 | 0.0011 | 0.1789 | 0.0006 |
| **17 modules** | |  |  |  |  |
| 1 | Ce_IL_VN_A-Ce_IL_DAN_A | 0.1886 | 0.0006 | 0.2113 | 0.0008 |
| 2 | Ce_IL_VN_A-Ce_IL_DAN_B | 0.2260 | 0.0001 | 0.0691 | 0.1099 |
| 3 | Ce_IL_VN_A-Ce_IL_LN_B | 0.1471 | 0.0009 | 0.1414 | 0.0038 |
| 4 | Ce_IL_VN_A-Ce_IL_DMN_C | 0.1357 | 0.0134 | 0.2361 | 0.0002 |
| 5 | Ce_IL_VN_B-Ce_IL_DAN_A | 0.1515 | 0.0021 | 0.1342 | 0.0153 |
| 6 | Ce_IL_VN_B-Ce_IL_DMN_C | 0.1061 | 0.0460 | 0.2758 | 0.0003 |
| 7 | Ce_IL_SMN_A-Ce_IL_DAN_B | 0.1909 | 0.0007 | 0.1700 | 0.0046 |
| 8 | Ce_IL_SMN_A-Ce_IL_FPN_B | 0.2024 | 0.0001 | 0.0640 | 0.1063 |
| 9 | Ce_IL_SMN_B-Ce_IL_TPN | 0.1818 | 0.0043 | 0.3427 | 0.0001 |
| 10 | Ce_IL_DAN_A-Ce_IL_TPN | 0.1677 | 0.0015 | 0.1523 | 0.0050 |
| 11 | Ce_IL_VAN_A-Ce_IL_TPN | 0.1399 | 0.0347 | 0.3676 | 0.0002 |
| 12 | Ce_IL_VAN_A-Ce_IL_DMN_A | 0.1123 | 0.0433 | 0.2813 | 0.0001 |
| 13 | Ce_IL_VAN_A-Ce_IL_DMN_B | 0.1540 | 0.0085 | 0.1926 | 0.0010 |
| 14 | Ce_IL_VAN_B-Ce_IL_DMN_B | 0.2354 | 0.0001 | 0.1136 | 0.0165 |
| 15 | Ce_IL_FPN_A-Ce_IL_DMN_A | 0.2325 | 0.0001 | 0.0883 | 0.0465 |
| 16 | Ce_IL_DMN_C-Ce_IL_DMN_A | -0.1527 | 0.0186 | -0.3888 | 0.0001 |
| 17 | Ce_CL_VN_A-Ce_CL_DAN_A | 0.2012 | 0.0003 | 0.2483 | 0.0003 |
| 18 | Ce_CL_VN_A-Ce_CL_DAN_B | 0.1759 | 0.0004 | 0.1245 | 0.0235 |
| 19 | Ce_CL_VN_A-Ce_CL_VAN_B | 0.1433 | 0.0031 | 0.1568 | 0.0020 |
| 20 | Ce_CL_VN_A-Ce_CL_FPN_A | 0.1890 | 0.0004 | 0.2260 | 0.0001 |
| 21 | Ce_CL_VN_B-Ce_CL_DAN_A | 0.1442 | 0.0027 | 0.1486 | 0.0050 |
| 22 | Ce_CL_VN_B-Ce_CL_VAN_B | 0.2397 | 0.0001 | 0.1464 | 0.0025 |
| 23 | Ce_CL_VN_B-Ce_CL_FPN_A | 0.2122 | 0.0001 | 0.1498 | 0.0029 |
| 24 | Ce_CL_VAN_B-Ce_CL_DMN_A | 0.1842 | 0.0019 | 0.2152 | 0.0004 |
| 25 | Ce_CL_VAN_B-Ce_CL_DMN_B | 0.1661 | 0.0031 | 0.2054 | 0.0010 |
| 26 | Ce_CL_FPN_A-Ce_CL_DMN_A | 0.1631 | 0.0047 | 0.1734 | 0.0038 |
| 27 | Ce_IL_VN_A-Ce_CL_VN_B | -0.1393 | 0.0033 | -0.1537 | 0.0064 |
| 28 | Ce_IL_SMN_A-Ce_CL_SMN_A | -0.3102 | 0.0001 | -0.2364 | 0.0030 |
| 29 | Ce_IL_SMN_A-Ce_CL_VAN_A | -0.2068 | 0.0007 | -0.1581 | 0.0028 |
| 30 | Ce_IL_SMN_B-Ce_CL_SMN_A | -0.1976 | 0.0053 | -0.2051 | 0.0047 |
| 31 | Ce_IL_SMN_B-Ce_CL_DAN_B | -0.1425 | 0.0122 | -0.2395 | 0.0002 |
| 32 | Ce_IL_DAN_A-Ce_CL_VN_A | 0.2100 | 0.0002 | 0.2227 | 0.0006 |
| 33 | Ce_IL_DAN_A-Ce_CL_FPN_A | -0.1176 | 0.0166 | -0.2834 | 0.0001 |
| 34 | Ce_IL_DAN_B-Ce_CL_VN_A | 0.2622 | 0.0001 | 0.1275 | 0.0166 |
| 35 | Ce_IL_DAN_B-Ce_CL_DAN_B | -0.2027 | 0.0007 | -0.1881 | 0.0043 |
| 36 | Ce_IL_DAN_B-Ce_CL_VAN_B | -0.1814 | 0.0010 | -0.1926 | 0.0005 |
| 37 | Ce_IL_DAN_B-Ce_CL_FPN_A | -0.1696 | 0.0003 | -0.2104 | 0.0003 |
| 38 | Ce_IL_VAN_A-Ce_CL_DAN_B | -0.2168 | 0.0017 | -0.3696 | 0.0001 |
| 39 | Ce_IL_VAN_A-Ce_CL_VAN_A | -0.4098 | 0.0001 | -0.2751 | 0.0012 |
| 40 | Ce_IL_VAN_A-Ce_CL_VAN_B | -0.2239 | 0.0002 | -0.3096 | 0.0001 |
| 41 | Ce_IL_VAN_A-Ce_CL_FPN_A | -0.1057 | 0.0203 | -0.2669 | 0.0001 |
| 42 | Ce_IL_FPN_C-Ce_CL_FPN_C | -0.1034 | 0.0396 | -0.3354 | 0.0001 |
| 43 | Ce_IL_FPN_C-Ce_CL_TPN | -0.1139 | 0.0159 | -0.3326 | 0.0001 |
| 44 | Ce_IL_FPN_A-Ce_CL_FPN_A | -0.1373 | 0.0039 | -0.1924 | 0.0007 |
| 45 | Ce_IL_TPN-Ce_CL_TPN | -0.1742 | 0.0036 | -0.2265 | 0.0007 |
| 46 | Ce_IL_TPN-Ce_CL_DMN_B | -0.1144 | 0.0160 | -0.3377 | 0.0001 |
| 47 | Ce_IL_DMN_C-Ce_CL_VN_A | 0.1178 | 0.0130 | 0.1890 | 0.0016 |
| 48 | Ce_IL_DMN_C-Ce_CL_DMN_C | -0.1778 | 0.0084 | -0.3544 | 0.0001 |
| 49 | Ce_IL_DMN_C-Ce_CL_DMN_A | -0.1352 | 0.0221 | -0.3678 | 0.0001 |
| 50 | Ce_IL_DMN_A-Ce_CL_DAN_B | 0.1205 | 0.0137 | 0.2505 | 0.0001 |
| 51 | Ce_IL_DMN_A-Ce_CL_VAN_B | 0.1394 | 0.0131 | 0.2519 | 0.0001 |
| 52 | Ce_IL_DMN_A-Ce_CL_FPN_A | 0.1523 | 0.0030 | 0.1917 | 0.0012 |
| 53 | Ce_IL_DMN_A-Ce_CL_DMN_B | -0.1118 | 0.0295 | -0.2470 | 0.0001 |
| 54 | Ce_IL_DMN_B-Ce_CL_DAN_B | 0.1200 | 0.0150 | 0.2012 | 0.0008 |
| 55 | Ce_IL_DMN_B-Ce_CL_VAN_B | 0.1501 | 0.0042 | 0.2247 | 0.0001 |
| 56 | Cb_IL_VN_B-Cb_IL_DAN_B | 0.1646 | 0.0003 | 0.0862 | 0.0221 |
| 57 | Cb_IL_VN_B-Cb_IL_FPN_A | 0.1774 | 0.0002 | 0.0608 | 0.1024 |
| 58 | Cb_IL_DAN_B-Cb_IL_LN_A | 0.1859 | 0.0003 | 0.1089 | 0.0263 |
| 59 | Cb_IL_DAN_B-Cb_IL_DMN_A | 0.2099 | 0.0005 | 0.2520 | 0.0002 |
| 60 | Cb_IL_VAN_A-Cb_IL_LN_A | 0.1735 | 0.0024 | 0.1553 | 0.0049 |
| 61 | Cb_IL_VAN_A-Cb_IL_FPN_B | 0.1880 | 0.0035 | 0.2502 | 0.0006 |
| 62 | Cb_IL_VAN_A-Cb_IL_DMN_A | 0.1754 | 0.0067 | 0.2891 | 0.0001 |
| 63 | Cb_IL_VAN_B-Cb_IL_FPN_B | 0.1825 | 0.0015 | 0.3192 | 0.0001 |
| 64 | Cb_IL_VAN_B-Cb_IL_DMN_A | 0.1419 | 0.0109 | 0.2361 | 0.0004 |
| 65 | Cb_IL_VAN_B-Cb_IL_DMN_B | 0.1661 | 0.0060 | 0.3285 | 0.0001 |
| 66 | Cb_IL_LN_A-Cb_IL_FPN_A | 0.2214 | 0.0001 | 0.1478 | 0.0033 |
| 67 | Cb_IL_LN_A-Cb_IL_FPN_B | 0.1982 | 0.0001 | 0.1502 | 0.0037 |
| 68 | Cb_IL_FPN_A-Cb_IL_FPN_B | 0.1688 | 0.0039 | 0.2145 | 0.0006 |
| 69 | Cb_IL_FPN_A-Cb_IL_DMN_A | 0.1690 | 0.0030 | 0.2387 | 0.0004 |
| 70 | Cb_IL_FPN_A-Cb_IL_DMN_B | 0.1901 | 0.0053 | 0.2943 | 0.0003 |
| 71 | Cb_CL_VN_B-Cb_CL_LN_A | 0.1010 | 0.0107 | 0.1389 | 0.0005 |
| 72 | Cb_CL_VN_B-Cb_CL_FPN_B | 0.1611 | 0.0003 | 0.0711 | 0.0530 |
| 73 | Cb_CL_VN_B-Cb_CL_DMN_A | 0.1566 | 0.0005 | 0.1105 | 0.0082 |
| 74 | Cb_CL_VN_B-Cb_CL_DMN_B | 0.1504 | 0.0002 | 0.0904 | 0.0163 |
| 75 | Cb_CL_SMN_A-Cb_CL_FPN_B | 0.1508 | 0.0039 | 0.1792 | 0.0005 |
| 76 | Cb_CL_SMN_A-Cb_CL_DMN_A | 0.1607 | 0.0026 | 0.1342 | 0.0090 |
| 77 | Cb_CL_SMN_B-Cb_CL_FPN_B | 0.2204 | 0.0001 | 0.2075 | 0.0001 |
| 78 | Cb_CL_SMN_B-Cb_CL_DMN_A | 0.2005 | 0.0003 | 0.2025 | 0.0002 |
| 79 | Cb_CL_SMN_B-Cb_CL_DMN_B | 0.1620 | 0.0006 | 0.1758 | 0.0003 |
| 80 | Cb_CL_DAN_A-Cb_CL_DMN_A | 0.1765 | 0.0012 | 0.1121 | 0.0201 |
| 81 | Cb_CL_DAN_A-Cb_CL_DMN_B | 0.1699 | 0.0007 | 0.1376 | 0.0029 |
| 82 | Cb_CL_DAN_B-Cb_CL_FPN_B | 0.2095 | 0.0004 | 0.1798 | 0.0008 |
| 83 | Cb_CL_DAN_B-Cb_CL_DMN_A | 0.2567 | 0.0001 | 0.2227 | 0.0002 |
| 84 | Cb_CL_DAN_B-Cb_CL_DMN_B | 0.1987 | 0.0003 | 0.1883 | 0.0009 |
| 85 | Cb_CL_VAN_A-Cb_CL_LN_A | 0.1772 | 0.0020 | 0.1840 | 0.0012 |
| 86 | Cb_CL_VAN_A-Cb_CL_FPN_A | 0.1225 | 0.0100 | 0.1865 | 0.0004 |
| 87 | Cb_CL_VAN_A-Cb_CL_FPN_B | 0.2716 | 0.0001 | 0.2797 | 0.0001 |
| 88 | Cb_CL_VAN_A-Cb_CL_TPN | -0.0996 | 0.0042 | -0.1125 | 0.0026 |
| 89 | Cb_CL_VAN_A-Cb_CL_DMN_A | 0.2808 | 0.0001 | 0.2212 | 0.0003 |
| 90 | Cb_CL_VAN_A-Cb_CL_DMN_B | 0.2203 | 0.0001 | 0.2426 | 0.0002 |
| 91 | Cb_CL_LN_A-Cb_CL_FPN_A | 0.1800 | 0.0004 | 0.1909 | 0.0002 |
| 92 | Cb_CL_LN_A-Cb_CL_FPN_B | 0.2502 | 0.0001 | 0.2275 | 0.0002 |
| 93 | Cb_CL_LN_A-Cb_CL_DMN_A | 0.2599 | 0.0001 | 0.1411 | 0.0050 |
| 94 | Cb_CL_LN_A-Cb_CL_DMN_B | 0.1979 | 0.0003 | 0.1908 | 0.0001 |
| 95 | Cb_IL_VN_B-Cb_CL_DAN_B | 0.1309 | 0.0008 | 0.0845 | 0.0253 |
| 96 | Cb_IL_SMN_A-Cb_CL_DMN_B | 0.1440 | 0.0011 | 0.1087 | 0.0069 |
| 97 | Cb_IL_SMN_B-Cb_CL_FPN_B | 0.1825 | 0.0008 | 0.1296 | 0.0047 |
| 98 | Cb_IL_SMN_B-Cb_CL_DMN_B | 0.1760 | 0.0003 | 0.1317 | 0.0021 |
| 99 | Cb_IL_DAN_B-Cb_CL_FPN_B | 0.1724 | 0.0018 | 0.1517 | 0.0019 |
| 100 | Cb_IL_DAN_B-Cb_CL_DMN_A | 0.2220 | 0.0001 | 0.1931 | 0.0005 |
| 101 | Cb_IL_DAN_B-Cb_CL_DMN_B | 0.1966 | 0.0003 | 0.1716 | 0.0005 |
| 102 | Cb_IL_VAN_A-Cb_CL_FPN_B | 0.2394 | 0.0003 | 0.2506 | 0.0001 |
| 103 | Cb_IL_VAN_A-Cb_CL_TPN | -0.1253 | 0.0006 | -0.1148 | 0.0022 |
| 104 | Cb_IL_VAN_A-Cb_CL_DMN_A | 0.2626 | 0.0003 | 0.2447 | 0.0002 |
| 105 | Cb_IL_VAN_A-Cb_CL_DMN_B | 0.2481 | 0.0003 | 0.2681 | 0.0001 |
| 106 | Cb_IL_VAN_B-Cb_CL_FPN_B | 0.1309 | 0.0190 | 0.2419 | 0.0001 |
| 107 | Cb_IL_VAN_B-Cb_CL_TPN | -0.0965 | 0.0117 | -0.1541 | 0.0005 |
| 108 | Cb_IL_VAN_B-Cb_CL_DMN_A | 0.1299 | 0.0183 | 0.2272 | 0.0002 |
| 109 | Cb_IL_VAN_B-Cb_CL_DMN_B | 0.1750 | 0.0027 | 0.3208 | 0.0001 |
| 110 | Cb_IL_LN_A-Cb_CL_DAN_B | 0.1793 | 0.0003 | 0.1502 | 0.0019 |
| 111 | Cb_IL_LN_A-Cb_CL_VAN_A | 0.2071 | 0.0004 | 0.1510 | 0.0039 |
| 112 | Cb_IL_LN_A-Cb_CL_FPN_A | 0.1965 | 0.0005 | 0.1749 | 0.0001 |
| 113 | Cb_IL_LN_A-Cb_CL_FPN_B | 0.2328 | 0.0001 | 0.2031 | 0.0001 |
| 114 | Cb_IL_LN_A-Cb_CL_DMN_A | 0.2179 | 0.0002 | 0.1288 | 0.0106 |
| 115 | Cb_IL_LN_A-Cb_CL_DMN_B | 0.2000 | 0.0001 | 0.2012 | 0.0001 |
| 116 | Cb_IL_FPN_A-Cb_CL_DMN_B | 0.1770 | 0.0050 | 0.1905 | 0.0026 |
| 117 | Cb_IL_FPN_B-Cb_CL_SMN_B | 0.1680 | 0.0002 | 0.1829 | 0.0005 |
| 118 | Cb_IL_FPN_B-Cb_CL_VAN_A | 0.1835 | 0.0006 | 0.1557 | 0.0045 |
| 119 | Cb_IL_TPN-Cb_CL_VN_B | 0.1333 | 0.0004 | 0.0988 | 0.0081 |
| 120 | Cb_IL_DMN_A-Cb_CL_VN_B | 0.1756 | 0.0001 | 0.1180 | 0.0048 |
| 121 | Cb_IL_DMN_A-Cb_CL_SMN_B | 0.1074 | 0.0148 | 0.1671 | 0.0005 |
| 122 | Cb_IL_DMN_A-Cb_CL_DAN_A | 0.1102 | 0.0048 | 0.1975 | 0.0001 |
| 123 | Cb_IL_DMN_A-Cb_CL_DAN_B | 0.2003 | 0.0002 | 0.2622 | 0.0001 |
| 124 | Cb_IL_DMN_A-Cb_CL_VAN_A | 0.2244 | 0.0001 | 0.2455 | 0.0001 |
| 125 | Cb_IL_DMN_A-Cb_CL_FPN_A | 0.1817 | 0.0006 | 0.1791 | 0.0008 |
| 126 | Ce_IL_SMN_B-Cb_C_VN_B | -0.1584 | 0.0009 | -0.0800 | 0.0394 |
| 127 | Ce_IL_SMN_B-Cb_C_SMN_B | -0.1446 | 0.0062 | -0.2200 | 0.0001 |
| 128 | Ce_IL_SMN_B-Cb_C_VAN_A | -0.1669 | 0.0016 | -0.1849 | 0.0026 |
| 129 | Ce_IL_DAN_B-Cb_C_DAN_B | -0.1096 | 0.0271 | -0.1819 | 0.0018 |
| 130 | Ce_IL_VAN_A-Cb_C_SMN_A | -0.1299 | 0.0058 | -0.1848 | 0.0003 |
| 131 | Ce_IL_VAN_A-Cb_C_SMN_B | -0.1337 | 0.0037 | -0.2215 | 0.0001 |
| 132 | Ce_IL_VAN_A-Cb_C_DAN_A | -0.0974 | 0.0131 | -0.1732 | 0.0001 |
| 133 | Ce_IL_VAN_A-Cb_C_DAN_B | -0.1633 | 0.0014 | -0.1876 | 0.0011 |
| 134 | Ce_IL_VAN_A-Cb_C_VAN_A | -0.1791 | 0.0001 | -0.2096 | 0.0004 |
| 135 | Ce_IL_LN_A-Cb_C_DAN_B | 0.0984 | 0.0221 | 0.1685 | 0.0006 |
| 136 | Ce_IL_LN_B-Cb_C_SMN_A | 0.1105 | 0.0058 | 0.1943 | 0.0001 |
| 137 | Ce_IL_FPN_A-Cb_C_FPN_A | -0.2806 | 0.0001 | -0.2307 | 0.0004 |
| 138 | Ce_IL_DMN_C-Cb_C_VAN_A | 0.1799 | 0.0001 | 0.0838 | 0.0422 |
| 139 | Ce_IL_VN_A-Cb_I_FPN_C | 0.1632 | 0.0006 | 0.0891 | 0.0290 |
| 140 | Ce_IL_VN_B-Cb_I_DAN_B | 0.1820 | 0.0002 | 0.0812 | 0.0523 |
| 141 | Ce_IL_SMN_B-Cb_I_VAN_A | -0.1262 | 0.0124 | -0.2124 | 0.0004 |
| 142 | Ce_IL_SMN_B-Cb_I_LN_A | -0.1189 | 0.0076 | -0.1844 | 0.0005 |
| 143 | Ce_IL_VAN_A-Cb_I_DAN_B | -0.1494 | 0.0034 | -0.2434 | 0.0001 |
| 144 | Ce_IL_VAN_A-Cb_I_VAN_A | -0.0999 | 0.0521 | -0.3205 | 0.0001 |
| 145 | Ce_IL_VAN_A-Cb_I_VAN_B | -0.0591 | 0.1544 | -0.3663 | 0.0001 |
| 146 | Ce_IL_VAN_A-Cb_I_LN_A | -0.1036 | 0.0148 | -0.1804 | 0.0004 |
| 147 | Ce_IL_VAN_B-Cb_I_VAN_B | -0.0949 | 0.0407 | -0.3831 | 0.0001 |
| 148 | Ce_IL_LN_A-Cb_I_DAN_B | 0.1175 | 0.0121 | 0.1455 | 0.0015 |
| 149 | Ce_IL_LN_B-Cb_I_DAN_B | 0.1362 | 0.0018 | 0.1548 | 0.0006 |
| 150 | Ce_IL_FPN_A-Cb_I_DAN_B | -0.1557 | 0.0002 | -0.0923 | 0.0268 |
| 151 | Ce_IL_FPN_A-Cb_I_FPN_A | -0.1471 | 0.0019 | -0.2125 | 0.0002 |
| 152 | Ce_IL_DMN_C-Cb_I_SMN_A | 0.1530 | 0.0011 | 0.1343 | 0.0080 |
| 153 | Ce_IL_DMN_C-Cb_I_DAN_B | 0.1153 | 0.0048 | 0.1545 | 0.0004 |
| 154 | Ce_IL_DMN_B-Cb_I_DAN_B | 0.1609 | 0.0007 | 0.1032 | 0.0214 |
| 155 | Ce_CL_VN_A-Cb_C_FPN_C | 0.1451 | 0.0015 | 0.1476 | 0.0016 |
| 156 | Ce_CL_LN_B-Cb_C_DAN_B | 0.1211 | 0.0068 | 0.1635 | 0.0003 |
| 157 | Ce_CL_FPN_C-Cb_C_FPN_A | 0.1239 | 0.0105 | 0.1528 | 0.0046 |
| 158 | Ce_CL_FPN_A-Cb_C_DAN_B | -0.1614 | 0.0012 | -0.1490 | 0.0057 |
| 159 | Ce_CL_DMN_C-Cb_C_FPN_A | 0.1067 | 0.0153 | 0.1668 | 0.0011 |
| 160 | Ce_CL_DMN_A-Cb_C_FPN_A | 0.1670 | 0.0031 | 0.2214 | 0.0007 |
| 161 | Ce_CL_DMN_A-Cb_C_DAN_B | 0.1269 | 0.0093 | 0.1606 | 0.0019 |
| 162 | Ce_CL_DMN_A-Cb_C_VAN_B | 0.0687 | 0.0934 | 0.2720 | 0.0001 |
| 163 | Ce_CL_DMN_B-Cb_C_FPN_A | 0.1786 | 0.0010 | 0.2235 | 0.0001 |
| 164 | Ce_CL_DMN_B-Cb_C_DAN_B | 0.1761 | 0.0030 | 0.1497 | 0.0059 |
| 165 | Ce_CL_DMN_B-Cb_C_VAN_A | 0.1502 | 0.0060 | 0.1990 | 0.0007 |
| 166 | Ce_CL_DMN_B-Cb_C_VAN_B | 0.1119 | 0.0142 | 0.2429 | 0.0001 |
| 167 | Ce_CL_VN_B-Cb_C_FPN_A | 0.1820 | 0.0008 | 0.1702 | 0.0027 |
| 168 | Ce_CL_VN_B-Cb_C_DAN_B | 0.1684 | 0.0007 | 0.1046 | 0.0249 |
| 169 | Ce_CL_SMN_A-Cb_C_FPN_B | 0.1638 | 0.0024 | 0.1211 | 0.0123 |
| 170 | Ce_CL_VAN_A-Cb_C_DAN_B | -0.1326 | 0.0059 | -0.1797 | 0.0020 |
| 171 | Ce_CL_VAN_B-Cb_C_DMN_A | 0.1440 | 0.0048 | 0.1387 | 0.0087 |
| 172 | Ce_CL_LN_A-Cb_C_DAN_B | 0.1702 | 0.0018 | 0.1371 | 0.0123 |
| 173 | Ce_CL_SMN_A-Cb_I_FPN_B | 0.2196 | 0.0002 | 0.1186 | 0.0150 |
| 174 | Ce_CL_SMN_A-Cb_I_DMN_B | 0.1551 | 0.0010 | 0.1466 | 0.0035 |
| 175 | Ce_CL_DAN_B-Cb_I_FPN_B | 0.1780 | 0.0006 | 0.1406 | 0.0047 |
| 176 | Ce_CL_DAN_B-Cb_I_DMN_A | 0.1312 | 0.0090 | 0.2307 | 0.0004 |
| 177 | Ce_CL_DAN_B-Cb_I_DMN_B | 0.1517 | 0.0015 | 0.1908 | 0.0006 |
| 178 | Ce_CL_VAN_A-Cb_I_SMN_B | -0.0977 | 0.0313 | -0.1897 | 0.0003 |
| 179 | Ce_CL_VAN_A-Cb_I_DAN_A | -0.1325 | 0.0024 | -0.1239 | 0.0054 |
| 180 | Ce_CL_VAN_A-Cb_I_VAN_A | -0.1702 | 0.0003 | -0.1488 | 0.0061 |
| 181 | Ce_CL_VAN_B-Cb_I_FPN_B | 0.1322 | 0.0062 | 0.2072 | 0.0005 |
| 182 | Ce_CL_VAN_B-Cb_I_DMN_B | 0.1423 | 0.0051 | 0.2233 | 0.0004 |
| 183 | Ce_CL_LN_A-Cb_I_DAN_B | 0.1476 | 0.0022 | 0.1639 | 0.0005 |
| 184 | Ce_CL_FPN_C-Cb_I_VAN_A | 0.0963 | 0.0106 | 0.1794 | 0.0007 |
| 185 | Ce_CL_FPN_C-Cb_I_FPN_A | 0.1612 | 0.0020 | 0.2627 | 0.0001 |
| 186 | Ce_CL_FPN_C-Cb_I_FPN_B | 0.0870 | 0.0409 | 0.2681 | 0.0001 |
| 187 | Ce_CL_FPN_C-Cb_I_DMN_B | 0.0665 | 0.0989 | 0.3160 | 0.0001 |
| 188 | Ce_CL_FPN_A-Cb_I_DAN_B | -0.1287 | 0.0036 | -0.1521 | 0.0050 |
| 189 | Ce_CL_DMN_A-Cb_I_VAN_A | 0.0963 | 0.0194 | 0.1870 | 0.0001 |
| 190 | Ce_CL_DMN_A-Cb_I_FPN_A | 0.1782 | 0.0013 | 0.1513 | 0.0049 |
| 191 | Ce_CL_DMN_A-Cb_I_TPN | 0.1316 | 0.0004 | 0.0558 | 0.0891 |
| 192 | Ce_CL_DMN_B-Cb_I_VN_B | 0.1165 | 0.0016 | 0.1074 | 0.0038 |
| 193 | Ce_CL_DMN_B-Cb_I_FPN_A | 0.1801 | 0.0003 | 0.0909 | 0.0492 |

Left lesion, left-hemisphere lesion group (n=36); Right lesion, right-hemisphere lesion group (n=29); Δ, Delta (patients - HC); HC, healthy controls. Ce, cerebrum; Cb, cerebellum; IL, ipsilesional; CL, contralesional; I, ipsilateral; C, contralateral; VN, visual network; SMN, somatomotor network; DAN, dorsal attention network; VAN, ventral attention network; LN, limbic network; FPN, frontoparietal network; TPN, temporal parietal network; DMN, default mode network.

**Table S5. Robustness analysis: modifying preprocessing steps.**

|  | **connections** | Original | | With scrubbing | | With GSR | | No lag | |
| --- | --- | --- | --- | --- | --- | --- | --- | --- | --- |
|  |  | **Δ** | ***p*** | **Δ** | ***p*** | **Δ** | ***p*** | **Δ** | ***p*** |
| **7 modules** | |  |  |  |  |  |  |  |  |
| 1 | Ce_IL_VN-Ce_IL_DAN | 0.2051 | 0.0001 | 0.2021 | 0.0001 | 0.1827 | 0.0002 | 0.1661 | 0.0003 |
| 2 | Ce_IL_SMN-Ce_IL_DAN | 0.1639 | 0.0005 | 0.1605 | 0.0007 | 0.1641 | 0.0003 | 0.1441 | 0.0026 |
| 3 | Ce_IL_SMN-Ce_IL_LN | 0.1353 | 0.0004 | 0.1349 | 0.0003 | 0.0919 | 0.0102 | 0.1285 | 0.0007 |
| 4 | Ce_IL_DAN-Ce_IL_DMN | 0.1493 | 0.0017 | 0.1457 | 0.0020 | 0.1477 | 0.0018 | 0.1695 | 0.0003 |
| 5 | Ce_IL_VAN-Ce_IL_DMN | 0.1924 | 0.0006 | 0.1941 | 0.0007 | 0.2057 | 0.0003 | 0.1890 | 0.0006 |
| 6 | Ce_IL_FPN-Ce_IL_DMN | 0.1844 | 0.0002 | 0.1886 | 0.0001 | 0.1864 | 0.0001 | 0.2024 | 0.0001 |
| 7 | Ce_CL_VN-Ce_CL_DAN | 0.2307 | 0.0001 | 0.2246 | 0.0001 | 0.1916 | 0.0002 | 0.1873 | 0.0001 |
| 8 | Ce_CL_VN-Ce_CL_VAN | 0.1500 | 0.0006 | 0.1514 | 0.0002 | 0.1329 | 0.0019 | 0.1345 | 0.0014 |
| 9 | Ce_CL_VN-Ce_CL_FPN | 0.2363 | 0.0001 | 0.2396 | 0.0001 | 0.2129 | 0.0001 | 0.2157 | 0.0001 |
| 10 | Ce_CL_FPN-Ce_CL_DMN | 0.2095 | 0.0001 | 0.2068 | 0.0001 | 0.2183 | 0.0001 | 0.2206 | 0.0001 |
| 11 | Ce_IL_SMN-Ce_CL_SMN | -0.2866 | 0.0002 | -0.2904 | 0.0002 | -0.2893 | 0.0002 | -0.2897 | 0.0001 |
| 12 | Ce_IL_DAN-Ce_CL_VN | 0.2048 | 0.0001 | 0.1984 | 0.0002 | 0.1731 | 0.0006 | 0.1830 | 0.0002 |
| 13 | Ce_IL_DAN-Ce_CL_DAN | -0.1957 | 0.0001 | -0.2016 | 0.0002 | -0.2050 | 0.0001 | -0.2180 | 0.0001 |
| 14 | Ce_IL_DAN-Ce_CL_FPN | -0.1341 | 0.0019 | -0.1278 | 0.0030 | -0.1485 | 0.0007 | -0.1271 | 0.0039 |
| 15 | Ce_IL_VAN-Ce_CL_DAN | -0.1952 | 0.0002 | -0.1979 | 0.0004 | -0.1994 | 0.0002 | -0.2019 | 0.0001 |
| 16 | Ce_IL_VAN-Ce_CL_VAN | -0.3240 | 0.0001 | -0.3284 | 0.0001 | -0.3220 | 0.0001 | -0.3077 | 0.0001 |
| 17 | Ce_IL_VAN-Ce_CL_FPN | -0.1875 | 0.0001 | -0.1824 | 0.0005 | -0.1940 | 0.0001 | -0.1984 | 0.0001 |
| 18 | Ce_IL_DMN-Ce_CL_DAN | 0.1848 | 0.0002 | 0.1768 | 0.0001 | 0.1646 | 0.0004 | 0.2004 | 0.0002 |
| 19 | Ce_IL_DMN-Ce_CL_VAN | 0.1746 | 0.0007 | 0.1763 | 0.0007 | 0.1792 | 0.0008 | 0.1771 | 0.0006 |
| 20 | Ce_IL_DMN-Ce_CL_FPN | 0.1679 | 0.0006 | 0.1668 | 0.0007 | 0.1712 | 0.0005 | 0.1992 | 0.0001 |
| 21 | Cb_IL_VN-Cb_IL_DAN | 0.1199 | 0.0009 | 0.1055 | 0.0015 | 0.1377 | 0.0001 | 0.1026 | 0.0029 |
| 22 | Cb_IL_VN-Cb_IL_FPN | 0.1473 | 0.0002 | 0.1667 | 0.0001 | 0.1849 | 0.0001 | 0.1649 | 0.0004 |
| 23 | Cb_IL_SMN-Cb_IL_LN | 0.1428 | 0.0023 | 0.1289 | 0.0081 | 0.1215 | 0.0152 | 0.1091 | 0.0233 |
| 24 | Cb_IL_DAN-Cb_IL_LN | 0.1856 | 0.0001 | 0.1809 | 0.0001 | 0.1758 | 0.0004 | 0.1664 | 0.0002 |
| 25 | Cb_IL_DAN-Cb_IL_DMN | 0.1921 | 0.0003 | 0.1973 | 0.0001 | 0.2469 | 0.0002 | 0.2062 | 0.0002 |
| 26 | Cb_IL_VAN-Cb_IL_LN | 0.1603 | 0.0004 | 0.1562 | 0.0010 | 0.1530 | 0.0011 | 0.1352 | 0.0037 |
| 27 | Cb_IL_VAN-Cb_IL_FPN | 0.2217 | 0.0002 | 0.2276 | 0.0004 | 0.2145 | 0.0005 | 0.2369 | 0.0001 |
| 28 | Cb_IL_VAN-Cb_IL_DMN | 0.2468 | 0.0001 | 0.2553 | 0.0001 | 0.2929 | 0.0001 | 0.2748 | 0.0001 |
| 29 | Cb_IL_LN-Cb_IL_FPN | 0.1821 | 0.0001 | 0.1823 | 0.0001 | 0.1970 | 0.0001 | 0.1722 | 0.0001 |
| 30 | Cb_IL_LN-Cb_IL_DMN | 0.1157 | 0.0018 | 0.1132 | 0.0030 | 0.1319 | 0.0013 | 0.0875 | 0.0191 |
| 31 | Cb_IL_FPN-Cb_IL_DMN | 0.1808 | 0.0008 | 0.1857 | 0.0011 | 0.2058 | 0.0006 | 0.2060 | 0.0004 |
| 32 | Cb_CL_VN-Cb_CL_LN | 0.1281 | 0.0002 | 0.1290 | 0.0001 | 0.1272 | 0.0002 | 0.1241 | 0.0002 |
| 33 | Cb_CL_VN-Cb_CL_DMN | 0.1363 | 0.0001 | 0.1360 | 0.0006 | 0.1452 | 0.0001 | 0.1257 | 0.0005 |
| 34 | Cb_CL_SMN-Cb_CL_DAN | 0.1298 | 0.0012 | 0.1222 | 0.0015 | 0.1292 | 0.0011 | 0.1432 | 0.0004 |
| 35 | Cb_CL_SMN-Cb_CL_FPN | 0.1795 | 0.0002 | 0.1753 | 0.0002 | 0.1883 | 0.0001 | 0.1853 | 0.0001 |
| 36 | Cb_CL_SMN-Cb_CL_DMN | 0.2090 | 0.0001 | 0.2056 | 0.0001 | 0.2068 | 0.0001 | 0.2125 | 0.0001 |
| 37 | Cb_CL_DAN-Cb_CL_LN | 0.1217 | 0.0025 | 0.1184 | 0.0034 | 0.1294 | 0.0014 | 0.1294 | 0.0003 |
| 38 | Cb_CL_DAN-Cb_CL_DMN | 0.2247 | 0.0001 | 0.2203 | 0.0002 | 0.2474 | 0.0001 | 0.2270 | 0.0001 |
| 39 | Cb_CL_VAN-Cb_CL_LN | 0.1856 | 0.0002 | 0.1838 | 0.0002 | 0.1849 | 0.0003 | 0.1659 | 0.0005 |
| 40 | Cb_CL_VAN-Cb_CL_FPN | 0.2022 | 0.0001 | 0.1955 | 0.0001 | 0.2063 | 0.0001 | 0.2011 | 0.0001 |
| 41 | Cb_CL_VAN-Cb_CL_DMN | 0.2359 | 0.0001 | 0.2340 | 0.0001 | 0.2476 | 0.0001 | 0.2301 | 0.0001 |
| 42 | Cb_CL_LN-Cb_CL_FPN | 0.1967 | 0.0001 | 0.1924 | 0.0002 | 0.2205 | 0.0001 | 0.1916 | 0.0001 |
| 43 | Cb_CL_LN-Cb_CL_DMN | 0.2317 | 0.0001 | 0.2285 | 0.0001 | 0.2414 | 0.0001 | 0.2074 | 0.0001 |
| 44 | Cb_IL_VN-Cb_CL_VN | 0.1260 | 0.0018 | 0.1177 | 0.0035 | 0.1270 | 0.0018 | 0.1309 | 0.0012 |
| 45 | Cb_IL_VN-Cb_CL_LN | 0.1536 | 0.0001 | 0.1799 | 0.0001 | 0.1808 | 0.0001 | 0.1799 | 0.0001 |
| 46 | Cb_IL_VN-Cb_CL_DMN | 0.1308 | 0.0002 | 0.1622 | 0.0001 | 0.1820 | 0.0001 | 0.1633 | 0.0001 |
| 47 | Cb_IL_SMN-Cb_CL_DMN | 0.1698 | 0.0002 | 0.1728 | 0.0004 | 0.1690 | 0.0003 | 0.1792 | 0.0001 |
| 48 | Cb_IL_DAN-Cb_CL_DMN | 0.2110 | 0.0001 | 0.2083 | 0.0001 | 0.2381 | 0.0001 | 0.2337 | 0.0001 |
| 49 | Cb_IL_VAN-Cb_CL_FPN | 0.2050 | 0.0002 | 0.2060 | 0.0004 | 0.1995 | 0.0004 | 0.2165 | 0.0001 |
| 50 | Cb_IL_VAN-Cb_CL_DMN | 0.2980 | 0.0001 | 0.2956 | 0.0001 | 0.3090 | 0.0001 | 0.3051 | 0.0001 |
| 51 | Cb_IL_LN-Cb_CL_VN | 0.1141 | 0.0006 | 0.1065 | 0.0032 | 0.1071 | 0.0019 | 0.1044 | 0.0022 |
| 52 | Cb_IL_LN-Cb_CL_DAN | 0.1993 | 0.0001 | 0.1968 | 0.0001 | 0.1951 | 0.0001 | 0.1918 | 0.0001 |
| 53 | Cb_IL_LN-Cb_CL_VAN | 0.1724 | 0.0001 | 0.1649 | 0.0005 | 0.1716 | 0.0004 | 0.1435 | 0.0022 |
| 54 | Cb_IL_LN-Cb_CL_FPN | 0.1903 | 0.0001 | 0.1750 | 0.0002 | 0.1993 | 0.0001 | 0.1741 | 0.0002 |
| 55 | Cb_IL_LN-Cb_CL_DMN | 0.2154 | 0.0001 | 0.2002 | 0.0001 | 0.2163 | 0.0001 | 0.1855 | 0.0001 |
| 56 | Cb_IL_FPN-Cb_CL_LN | 0.1331 | 0.0022 | 0.1258 | 0.0038 | 0.1608 | 0.0002 | 0.1331 | 0.0014 |
| 57 | Cb_IL_DMN-Cb_CL_VN | 0.1409 | 0.0003 | 0.1457 | 0.0002 | 0.1710 | 0.0001 | 0.1350 | 0.0004 |
| 58 | Cb_IL_DMN-Cb_CL_SMN | 0.1217 | 0.0010 | 0.1271 | 0.0009 | 0.1389 | 0.0012 | 0.1306 | 0.0007 |
| 59 | Cb_IL_DMN-Cb_CL_DAN | 0.2012 | 0.0003 | 0.2063 | 0.0002 | 0.2328 | 0.0001 | 0.2121 | 0.0002 |
| 60 | Cb_IL_DMN-Cb_CL_VAN | 0.1767 | 0.0003 | 0.1861 | 0.0001 | 0.2251 | 0.0001 | 0.1854 | 0.0001 |
| 61 | Ce_IL_SMN-Cb_C_VN | -0.1424 | 0.0001 | -0.1369 | 0.0003 | -0.1434 | 0.0004 | -0.1454 | 0.0002 |
| 62 | Ce_IL_SMN-Cb_C_SMN | -0.1788 | 0.0003 | -0.1759 | 0.0004 | -0.1958 | 0.0003 | -0.1709 | 0.0003 |
| 63 | Ce_IL_SMN-Cb_C_VAN | -0.1380 | 0.0023 | -0.1373 | 0.0022 | -0.1686 | 0.0004 | -0.1525 | 0.0013 |
| 64 | Ce_IL_DAN-Cb_C_DAN | -0.2006 | 0.0001 | -0.2072 | 0.0001 | -0.2195 | 0.0001 | -0.1885 | 0.0002 |
| 65 | Ce_IL_VAN-Cb_C_SMN | -0.1684 | 0.0002 | -0.1682 | 0.0002 | -0.1687 | 0.0002 | -0.1575 | 0.0003 |
| 66 | Ce_IL_VAN-Cb_C_DAN | -0.1991 | 0.0001 | -0.1959 | 0.0001 | -0.2248 | 0.0001 | -0.2142 | 0.0001 |
| 67 | Ce_IL_VAN-Cb_C_VAN | -0.1579 | 0.0005 | -0.1596 | 0.0009 | -0.1675 | 0.0005 | -0.1663 | 0.0004 |
| 68 | Ce_IL_FPN-Cb_C_DAN | -0.1518 | 0.0007 | -0.1508 | 0.0009 | -0.1760 | 0.0001 | -0.1388 | 0.0016 |
| 69 | Ce_IL_FPN-Cb_C_FPN | -0.1625 | 0.0017 | -0.1652 | 0.0010 | -0.1834 | 0.0006 | -0.1778 | 0.0005 |
| 70 | Ce_IL_DMN-Cb_C_VN | 0.0941 | 0.0015 | 0.0958 | 0.0031 | 0.0881 | 0.0032 | 0.0951 | 0.0021 |
| 71 | Ce_IL_DMN-Cb_C_DAN | 0.1148 | 0.0018 | 0.1187 | 0.0031 | 0.0889 | 0.0138 | 0.1131 | 0.0031 |
| 72 | Ce_IL_VN-Cb_I_DAN | 0.1537 | 0.0005 | 0.1690 | 0.0002 | 0.1410 | 0.0018 | 0.1542 | 0.0006 |
| 73 | Ce_IL_SMN-Cb_I_VN | -0.1424 | 0.0001 | -0.1809 | 0.0001 | -0.1749 | 0.0002 | -0.1725 | 0.0001 |
| 74 | Ce_IL_SMN-Cb_I_SMN | -0.1579 | 0.0010 | -0.1621 | 0.0009 | -0.1739 | 0.0002 | -0.1554 | 0.0014 |
| 75 | Ce_IL_SMN-Cb_I_VAN | -0.1852 | 0.0008 | -0.1918 | 0.0005 | -0.1883 | 0.0001 | -0.1853 | 0.0003 |
| 76 | Ce_IL_VAN-Cb_I_VN | -0.1036 | 0.0015 | -0.1084 | 0.0016 | -0.1117 | 0.0010 | -0.1108 | 0.0008 |
| 77 | Ce_IL_VAN-Cb_I_DAN | -0.1849 | 0.0002 | -0.1863 | 0.0002 | -0.2052 | 0.0001 | -0.1887 | 0.0002 |
| 78 | Ce_IL_VAN-Cb_I_VAN | -0.2240 | 0.0001 | -0.2237 | 0.0001 | -0.2195 | 0.0001 | -0.2277 | 0.0001 |
| 79 | Ce_IL_VAN-Cb_I_LN | -0.1330 | 0.0003 | -0.1147 | 0.0026 | -0.1199 | 0.0009 | -0.1141 | 0.0024 |
| 80 | Ce_IL_LN-Cb_I_SMN | 0.1498 | 0.0010 | 0.1485 | 0.0009 | 0.1469 | 0.0033 | 0.1257 | 0.0063 |
| 81 | Ce_IL_LN-Cb_I_DAN | 0.1438 | 0.0002 | 0.1470 | 0.0004 | 0.1394 | 0.0002 | 0.1590 | 0.0003 |
| 82 | Ce_IL_DMN-Cb_I_DAN | 0.1275 | 0.0016 | 0.1285 | 0.0027 | 0.1287 | 0.0029 | 0.1404 | 0.0013 |
| 83 | Ce_IL_DMN-Cb_I_VAN | 0.1365 | 0.0018 | 0.1410 | 0.0012 | 0.1276 | 0.0028 | 0.1333 | 0.0024 |
| 84 | Ce_CL_VN-Cb_C_DAN | 0.1719 | 0.0004 | 0.1718 | 0.0001 | 0.1529 | 0.0005 | 0.1539 | 0.0006 |
| 85 | Ce_CL_SMN-Cb_C_VN | -0.1081 | 0.0017 | -0.1064 | 0.0015 | -0.0998 | 0.0044 | -0.0985 | 0.0034 |
| 86 | Ce_CL_SMN-Cb_C_FPN | 0.1416 | 0.0018 | 0.1344 | 0.0032 | 0.1242 | 0.0059 | 0.1315 | 0.0025 |
| 87 | Ce_CL_VAN-Cb_C_DAN | -0.1446 | 0.0017 | -0.1452 | 0.0019 | -0.1553 | 0.0012 | -0.1501 | 0.0013 |
| 88 | Ce_CL_VAN-Cb_C_VAN | -0.1957 | 0.0002 | -0.1992 | 0.0002 | -0.1811 | 0.0005 | -0.1934 | 0.0002 |
| 89 | Ce_CL_LN-Cb_C_SMN | 0.1555 | 0.0008 | 0.1529 | 0.0013 | 0.1703 | 0.0004 | 0.1570 | 0.0008 |
| 90 | Ce_CL_DMN-Cb_C_DAN | 0.1639 | 0.0004 | 0.1670 | 0.0003 | 0.1611 | 0.0002 | 0.1670 | 0.0002 |
| 91 | Ce_CL_DMN-Cb_C_VAN | 0.1938 | 0.0001 | 0.2030 | 0.0001 | 0.2014 | 0.0001 | 0.2001 | 0.0002 |
| 92 | Ce_CL_DAN-Cb_I_DMN | 0.1807 | 0.0001 | 0.1779 | 0.0002 | 0.1774 | 0.0002 | 0.1926 | 0.0002 |
| 93 | Ce_CL_VAN-Cb_I_DAN | -0.1364 | 0.0018 | -0.1390 | 0.0014 | -0.1594 | 0.0007 | -0.1511 | 0.0009 |
| 94 | Ce_CL_VAN-Cb_I_DMN | 0.1408 | 0.0017 | 0.1324 | 0.0020 | 0.1348 | 0.0032 | 0.1441 | 0.0012 |
| 95 | Ce_CL_LN-Cb_I_DAN | 0.1656 | 0.0001 | 0.1602 | 0.0002 | 0.1224 | 0.0021 | 0.1695 | 0.0002 |
| 96 | Ce_CL_LN-Cb_I_VAN | 0.1680 | 0.0006 | 0.1655 | 0.0004 | 0.1449 | 0.0011 | 0.1542 | 0.0012 |
| 97 | Ce_CL_FPN-Cb_I_DMN | 0.1430 | 0.0005 | 0.1376 | 0.0010 | 0.1673 | 0.0001 | 0.1585 | 0.0004 |
| 98 | Ce_CL_DMN-Cb_I_VN | 0.0921 | 0.0018 | 0.0957 | 0.0015 | 0.0889 | 0.0023 | 0.0932 | 0.0020 |
| 99 | Ce_CL_DMN-Cb_I_DAN | 0.1529 | 0.0003 | 0.1543 | 0.0006 | 0.1361 | 0.0005 | 0.1445 | 0.0005 |
| 100 | Ce_CL_DMN-Cb_I_VAN | 0.1581 | 0.0001 | 0.1603 | 0.0001 | 0.1344 | 0.0005 | 0.1260 | 0.0007 |
| **17 modules** | |  |  |  |  |  |  |  |  |
| 1 | Ce_IL_VN_A-Ce_IL_DAN_A | 0.1995 | 0.0001 | 0.1907 | 0.0001 | 0.1812 | 0.0004 | 0.1287 | 0.0045 |
| 2 | Ce_IL_VN_A-Ce_IL_DAN_B | 0.1566 | 0.0008 | 0.1552 | 0.0012 | 0.1518 | 0.0009 | 0.1290 | 0.0028 |
| 3 | Ce_IL_VN_A-Ce_IL_LN_B | 0.1423 | 0.0005 | 0.1312 | 0.0005 | 0.0606 | 0.0610 | 0.1377 | 0.0005 |
| 4 | Ce_IL_VN_A-Ce_IL_DMN_C | 0.1826 | 0.0007 | 0.1721 | 0.0004 | 0.1383 | 0.0032 | 0.1621 | 0.0013 |
| 5 | Ce_IL_VN_B-Ce_IL_DAN_A | 0.1460 | 0.0005 | 0.1259 | 0.0046 | 0.1452 | 0.0023 | 0.0979 | 0.0234 |
| 6 | Ce_IL_VN_B-Ce_IL_DMN_C | 0.1839 | 0.0005 | 0.1681 | 0.0017 | 0.1806 | 0.0005 | 0.1503 | 0.0047 |
| 7 | Ce_IL_SMN_A-Ce_IL_DAN_B | 0.1813 | 0.0003 | 0.1776 | 0.0005 | 0.2024 | 0.0001 | 0.1679 | 0.0006 |
| 8 | Ce_IL_SMN_A-Ce_IL_FPN_B | 0.1424 | 0.0008 | 0.1425 | 0.0003 | 0.1490 | 0.0003 | 0.1308 | 0.0006 |
| 9 | Ce_IL_SMN_B-Ce_IL_TPN | 0.2544 | 0.0001 | 0.2525 | 0.0001 | 0.2694 | 0.0001 | 0.2707 | 0.0001 |
| 10 | Ce_IL_DAN_A-Ce_IL_TPN | 0.1622 | 0.0007 | 0.1615 | 0.0004 | 0.1705 | 0.0005 | 0.1636 | 0.0005 |
| 11 | Ce_IL_VAN_A-Ce_IL_TPN | 0.2432 | 0.0002 | 0.2387 | 0.0003 | 0.2540 | 0.0001 | 0.2541 | 0.0001 |
| 12 | Ce_IL_VAN_A-Ce_IL_DMN_A | 0.1896 | 0.0003 | 0.1928 | 0.0003 | 0.1923 | 0.0002 | 0.1780 | 0.0006 |
| 13 | Ce_IL_VAN_A-Ce_IL_DMN_B | 0.1745 | 0.0001 | 0.1735 | 0.0003 | 0.1875 | 0.0001 | 0.1776 | 0.0006 |
| 14 | Ce_IL_VAN_B-Ce_IL_DMN_B | 0.1841 | 0.0003 | 0.1823 | 0.0002 | 0.1904 | 0.0001 | 0.1805 | 0.0002 |
| 15 | Ce_IL_FPN_A-Ce_IL_DMN_A | 0.1696 | 0.0003 | 0.1739 | 0.0001 | 0.1692 | 0.0005 | 0.1728 | 0.0002 |
| 16 | Ce_IL_DMN_C-Ce_IL_DMN_A | -0.2568 | 0.0002 | -0.2588 | 0.0001 | -0.2496 | 0.0001 | -0.2503 | 0.0001 |
| 17 | Ce_CL_VN_A-Ce_CL_DAN_A | 0.2238 | 0.0001 | 0.2221 | 0.0003 | 0.1857 | 0.0005 | 0.1626 | 0.0013 |
| 18 | Ce_CL_VN_A-Ce_CL_DAN_B | 0.1540 | 0.0005 | 0.1521 | 0.0002 | 0.1220 | 0.0037 | 0.1165 | 0.0043 |
| 19 | Ce_CL_VN_A-Ce_CL_VAN_B | 0.1508 | 0.0008 | 0.1555 | 0.0002 | 0.1229 | 0.0021 | 0.1274 | 0.0039 |
| 20 | Ce_CL_VN_A-Ce_CL_FPN_A | 0.2073 | 0.0002 | 0.2110 | 0.0001 | 0.1932 | 0.0001 | 0.1740 | 0.0002 |
| 21 | Ce_CL_VN_B-Ce_CL_DAN_A | 0.1472 | 0.0005 | 0.1437 | 0.0009 | 0.1357 | 0.0019 | 0.1112 | 0.0056 |
| 22 | Ce_CL_VN_B-Ce_CL_VAN_B | 0.1994 | 0.0001 | 0.2014 | 0.0001 | 0.1813 | 0.0002 | 0.1911 | 0.0001 |
| 23 | Ce_CL_VN_B-Ce_CL_FPN_A | 0.1853 | 0.0002 | 0.1855 | 0.0001 | 0.1828 | 0.0001 | 0.1705 | 0.0001 |
| 24 | Ce_CL_VAN_B-Ce_CL_DMN_A | 0.2013 | 0.0003 | 0.2005 | 0.0001 | 0.2051 | 0.0001 | 0.1903 | 0.0003 |
| 25 | Ce_CL_VAN_B-Ce_CL_DMN_B | 0.1865 | 0.0001 | 0.1825 | 0.0001 | 0.1919 | 0.0001 | 0.1976 | 0.0001 |
| 26 | Ce_CL_FPN_A-Ce_CL_DMN_A | 0.1713 | 0.0002 | 0.1685 | 0.0010 | 0.1736 | 0.0006 | 0.1810 | 0.0001 |
| 27 | Ce_IL_VN_A-Ce_CL_VN_B | -0.1494 | 0.0008 | -0.1540 | 0.0006 | -0.1432 | 0.0028 | -0.1772 | 0.0004 |
| 28 | Ce_IL_SMN_A-Ce_CL_SMN_A | -0.2797 | 0.0001 | -0.2787 | 0.0001 | -0.2557 | 0.0001 | -0.2856 | 0.0001 |
| 29 | Ce_IL_SMN_A-Ce_CL_VAN_A | -0.1837 | 0.0004 | -0.1834 | 0.0002 | -0.1839 | 0.0001 | -0.1771 | 0.0006 |
| 30 | Ce_IL_SMN_B-Ce_CL_SMN_A | -0.2016 | 0.0005 | -0.2074 | 0.0014 | -0.2091 | 0.0005 | -0.2006 | 0.0005 |
| 31 | Ce_IL_SMN_B-Ce_CL_DAN_B | -0.1865 | 0.0002 | -0.1958 | 0.0001 | -0.1909 | 0.0003 | -0.1828 | 0.0004 |
| 32 | Ce_IL_DAN_A-Ce_CL_VN_A | 0.2156 | 0.0001 | 0.2121 | 0.0002 | 0.1771 | 0.0002 | 0.1632 | 0.0007 |
| 33 | Ce_IL_DAN_A-Ce_CL_FPN_A | -0.1958 | 0.0001 | -0.1907 | 0.0001 | -0.1931 | 0.0001 | -0.1829 | 0.0001 |
| 34 | Ce_IL_DAN_B-Ce_CL_VN_A | 0.2012 | 0.0001 | 0.2060 | 0.0001 | 0.1769 | 0.0003 | 0.1787 | 0.0002 |
| 35 | Ce_IL_DAN_B-Ce_CL_DAN_B | -0.1998 | 0.0002 | -0.2016 | 0.0003 | -0.1973 | 0.0005 | -0.2166 | 0.0002 |
| 36 | Ce_IL_DAN_B-Ce_CL_VAN_B | -0.1867 | 0.0002 | -0.1795 | 0.0001 | -0.1883 | 0.0001 | -0.1885 | 0.0001 |
| 37 | Ce_IL_DAN_B-Ce_CL_FPN_A | -0.1925 | 0.0001 | -0.1894 | 0.0001 | -0.1985 | 0.0001 | -0.2016 | 0.0001 |
| 38 | Ce_IL_VAN_A-Ce_CL_DAN_B | -0.2868 | 0.0001 | -0.2924 | 0.0001 | -0.2930 | 0.0001 | -0.2816 | 0.0001 |
| 39 | Ce_IL_VAN_A-Ce_CL_VAN_A | -0.3529 | 0.0001 | -0.3568 | 0.0001 | -0.3575 | 0.0001 | -0.3481 | 0.0001 |
| 40 | Ce_IL_VAN_A-Ce_CL_VAN_B | -0.2644 | 0.0001 | -0.2631 | 0.0001 | -0.2542 | 0.0001 | -0.2591 | 0.0001 |
| 41 | Ce_IL_VAN_A-Ce_CL_FPN_A | -0.1798 | 0.0001 | -0.1818 | 0.0002 | -0.1868 | 0.0001 | -0.1851 | 0.0001 |
| 42 | Ce_IL_FPN_C-Ce_CL_FPN_C | -0.2060 | 0.0001 | -0.2021 | 0.0001 | -0.2001 | 0.0002 | -0.2138 | 0.0001 |
| 43 | Ce_IL_FPN_C-Ce_CL_TPN | -0.2133 | 0.0001 | -0.2036 | 0.0001 | -0.2141 | 0.0001 | -0.2227 | 0.0001 |
| 44 | Ce_IL_FPN_A-Ce_CL_FPN_A | -0.1661 | 0.0003 | -0.1614 | 0.0008 | -0.1662 | 0.0006 | -0.1487 | 0.0010 |
| 45 | Ce_IL_TPN-Ce_CL_TPN | -0.1975 | 0.0002 | -0.1950 | 0.0003 | -0.1942 | 0.0002 | -0.1827 | 0.0006 |
| 46 | Ce_IL_TPN-Ce_CL_DMN_B | -0.2151 | 0.0001 | -0.2088 | 0.0001 | -0.2338 | 0.0001 | -0.2114 | 0.0001 |
| 47 | Ce_IL_DMN_C-Ce_CL_VN_A | 0.1492 | 0.0008 | 0.1440 | 0.0011 | 0.1224 | 0.0034 | 0.1290 | 0.0031 |
| 48 | Ce_IL_DMN_C-Ce_CL_DMN_C | -0.2588 | 0.0001 | -0.2547 | 0.0001 | -0.2658 | 0.0001 | -0.2760 | 0.0001 |
| 49 | Ce_IL_DMN_C-Ce_CL_DMN_A | -0.2399 | 0.0001 | -0.2389 | 0.0001 | -0.2486 | 0.0001 | -0.2394 | 0.0001 |
| 50 | Ce_IL_DMN_A-Ce_CL_DAN_B | 0.1802 | 0.0002 | 0.1765 | 0.0001 | 0.1720 | 0.0003 | 0.1746 | 0.0002 |
| 51 | Ce_IL_DMN_A-Ce_CL_VAN_B | 0.1932 | 0.0002 | 0.1977 | 0.0001 | 0.1925 | 0.0003 | 0.1856 | 0.0006 |
| 52 | Ce_IL_DMN_A-Ce_CL_FPN_A | 0.1727 | 0.0001 | 0.1672 | 0.0003 | 0.1788 | 0.0002 | 0.1932 | 0.0001 |
| 53 | Ce_IL_DMN_A-Ce_CL_DMN_B | -0.1701 | 0.0001 | -0.1701 | 0.0007 | -0.1772 | 0.0006 | -0.1847 | 0.0001 |
| 54 | Ce_IL_DMN_B-Ce_CL_DAN_B | 0.1599 | 0.0006 | 0.1570 | 0.0005 | 0.1598 | 0.0005 | 0.1701 | 0.0002 |
| 55 | Ce_IL_DMN_B-Ce_CL_VAN_B | 0.1871 | 0.0001 | 0.1845 | 0.0001 | 0.1957 | 0.0001 | 0.2007 | 0.0001 |
| 56 | Cb_IL_VN_B-Cb_IL_DAN_B | 0.1305 | 0.0002 | 0.1240 | 0.0006 | 0.1425 | 0.0002 | 0.1188 | 0.0008 |
| 57 | Cb_IL_VN_B-Cb_IL_FPN_A | 0.1275 | 0.0007 | 0.1783 | 0.0001 | 0.1961 | 0.0001 | 0.1818 | 0.0001 |
| 58 | Cb_IL_DAN_B-Cb_IL_LN_A | 0.1516 | 0.0003 | 0.1466 | 0.0009 | 0.1377 | 0.0020 | 0.1347 | 0.0008 |
| 59 | Cb_IL_DAN_B-Cb_IL_DMN_A | 0.2294 | 0.0001 | 0.2303 | 0.0002 | 0.2339 | 0.0001 | 0.2429 | 0.0001 |
| 60 | Cb_IL_VAN_A-Cb_IL_LN_A | 0.1665 | 0.0003 | 0.1606 | 0.0002 | 0.1518 | 0.0010 | 0.1371 | 0.0019 |
| 61 | Cb_IL_VAN_A-Cb_IL_FPN_B | 0.2173 | 0.0002 | 0.2202 | 0.0001 | 0.2415 | 0.0001 | 0.2458 | 0.0001 |
| 62 | Cb_IL_VAN_A-Cb_IL_DMN_A | 0.2287 | 0.0001 | 0.2362 | 0.0001 | 0.2372 | 0.0001 | 0.2333 | 0.0001 |
| 63 | Cb_IL_VAN_B-Cb_IL_FPN_B | 0.2438 | 0.0001 | 0.2534 | 0.0001 | 0.2644 | 0.0001 | 0.2664 | 0.0001 |
| 64 | Cb_IL_VAN_B-Cb_IL_DMN_A | 0.1836 | 0.0006 | 0.2052 | 0.0003 | 0.2447 | 0.0001 | 0.2441 | 0.0002 |
| 65 | Cb_IL_VAN_B-Cb_IL_DMN_B | 0.2401 | 0.0001 | 0.2452 | 0.0001 | 0.2574 | 0.0002 | 0.2449 | 0.0001 |
| 66 | Cb_IL_LN_A-Cb_IL_FPN_A | 0.1904 | 0.0002 | 0.1850 | 0.0003 | 0.1860 | 0.0001 | 0.1805 | 0.0002 |
| 67 | Cb_IL_LN_A-Cb_IL_FPN_B | 0.1793 | 0.0001 | 0.1816 | 0.0002 | 0.1841 | 0.0001 | 0.1680 | 0.0002 |
| 68 | Cb_IL_FPN_A-Cb_IL_FPN_B | 0.1899 | 0.0001 | 0.1855 | 0.0002 | 0.2405 | 0.0001 | 0.1512 | 0.0022 |
| 69 | Cb_IL_FPN_A-Cb_IL_DMN_A | 0.2024 | 0.0001 | 0.2077 | 0.0001 | 0.2165 | 0.0001 | 0.2193 | 0.0001 |
| 70 | Cb_IL_FPN_A-Cb_IL_DMN_B | 0.2375 | 0.0001 | 0.2309 | 0.0001 | 0.2650 | 0.0001 | 0.2328 | 0.0003 |
| 71 | Cb_CL_VN_B-Cb_CL_LN_A | 0.1196 | 0.0007 | 0.1185 | 0.0009 | 0.1203 | 0.0005 | 0.1181 | 0.0003 |
| 72 | Cb_CL_VN_B-Cb_CL_FPN_B | 0.1226 | 0.0005 | 0.1209 | 0.0007 | 0.1179 | 0.0004 | 0.1000 | 0.0028 |
| 73 | Cb_CL_VN_B-Cb_CL_DMN_A | 0.1380 | 0.0004 | 0.1374 | 0.0002 | 0.1462 | 0.0001 | 0.1320 | 0.0006 |
| 74 | Cb_CL_VN_B-Cb_CL_DMN_B | 0.1251 | 0.0004 | 0.1213 | 0.0005 | 0.1417 | 0.0002 | 0.1173 | 0.0001 |
| 75 | Cb_CL_SMN_A-Cb_CL_FPN_B | 0.1661 | 0.0001 | 0.1639 | 0.0003 | 0.1726 | 0.0001 | 0.1891 | 0.0002 |
| 76 | Cb_CL_SMN_A-Cb_CL_DMN_A | 0.1509 | 0.0003 | 0.1445 | 0.0008 | 0.1276 | 0.0027 | 0.1550 | 0.0004 |
| 77 | Cb_CL_SMN_B-Cb_CL_FPN_B | 0.2170 | 0.0001 | 0.2204 | 0.0001 | 0.2242 | 0.0001 | 0.2210 | 0.0001 |
| 78 | Cb_CL_SMN_B-Cb_CL_DMN_A | 0.2038 | 0.0001 | 0.2036 | 0.0001 | 0.1936 | 0.0001 | 0.1998 | 0.0001 |
| 79 | Cb_CL_SMN_B-Cb_CL_DMN_B | 0.1702 | 0.0001 | 0.1709 | 0.0002 | 0.1779 | 0.0001 | 0.1630 | 0.0002 |
| 80 | Cb_CL_DAN_A-Cb_CL_DMN_A | 0.1504 | 0.0005 | 0.1414 | 0.0015 | 0.1512 | 0.0012 | 0.1330 | 0.0030 |
| 81 | Cb_CL_DAN_A-Cb_CL_DMN_B | 0.1576 | 0.0002 | 0.1541 | 0.0006 | 0.1701 | 0.0003 | 0.1525 | 0.0005 |
| 82 | Cb_CL_DAN_B-Cb_CL_FPN_B | 0.1983 | 0.0001 | 0.1996 | 0.0001 | 0.2035 | 0.0001 | 0.2185 | 0.0001 |
| 83 | Cb_CL_DAN_B-Cb_CL_DMN_A | 0.2431 | 0.0001 | 0.2364 | 0.0001 | 0.2409 | 0.0001 | 0.2630 | 0.0001 |
| 84 | Cb_CL_DAN_B-Cb_CL_DMN_B | 0.1953 | 0.0001 | 0.1968 | 0.0001 | 0.2134 | 0.0001 | 0.1982 | 0.0001 |
| 85 | Cb_CL_VAN_A-Cb_CL_LN_A | 0.1802 | 0.0001 | 0.1780 | 0.0001 | 0.1750 | 0.0004 | 0.1680 | 0.0002 |
| 86 | Cb_CL_VAN_A-Cb_CL_FPN_A | 0.1548 | 0.0002 | 0.1471 | 0.0010 | 0.1741 | 0.0003 | 0.1428 | 0.0014 |
| 87 | Cb_CL_VAN_A-Cb_CL_FPN_B | 0.2789 | 0.0001 | 0.2754 | 0.0001 | 0.2720 | 0.0001 | 0.2782 | 0.0001 |
| 88 | Cb_CL_VAN_A-Cb_CL_TPN | -0.1055 | 0.0004 | -0.1051 | 0.0009 | -0.0949 | 0.0015 | -0.0942 | 0.0011 |
| 89 | Cb_CL_VAN_A-Cb_CL_DMN_A | 0.2567 | 0.0001 | 0.2516 | 0.0001 | 0.2575 | 0.0001 | 0.2603 | 0.0001 |
| 90 | Cb_CL_VAN_A-Cb_CL_DMN_B | 0.2339 | 0.0001 | 0.2325 | 0.0001 | 0.2515 | 0.0001 | 0.2285 | 0.0001 |
| 91 | Cb_CL_LN_A-Cb_CL_FPN_A | 0.1869 | 0.0001 | 0.1809 | 0.0001 | 0.2142 | 0.0001 | 0.1960 | 0.0001 |
| 92 | Cb_CL_LN_A-Cb_CL_FPN_B | 0.2427 | 0.0001 | 0.2395 | 0.0001 | 0.2553 | 0.0001 | 0.2244 | 0.0001 |
| 93 | Cb_CL_LN_A-Cb_CL_DMN_A | 0.2099 | 0.0002 | 0.2063 | 0.0001 | 0.1948 | 0.0001 | 0.1828 | 0.0001 |
| 94 | Cb_CL_LN_A-Cb_CL_DMN_B | 0.1975 | 0.0001 | 0.1934 | 0.0001 | 0.2111 | 0.0001 | 0.1768 | 0.0001 |
| 95 | Cb_IL_VN_B-Cb_CL_DAN_B | 0.1109 | 0.0007 | 0.1168 | 0.0004 | 0.1307 | 0.0002 | 0.1116 | 0.0003 |
| 96 | Cb_IL_SMN_A-Cb_CL_DMN_B | 0.1288 | 0.0006 | 0.1317 | 0.0002 | 0.1272 | 0.0010 | 0.1330 | 0.0004 |
| 97 | Cb_IL_SMN_B-Cb_CL_FPN_B | 0.1621 | 0.0004 | 0.1648 | 0.0004 | 0.1716 | 0.0001 | 0.1709 | 0.0003 |
| 98 | Cb_IL_SMN_B-Cb_CL_DMN_B | 0.1581 | 0.0001 | 0.1606 | 0.0002 | 0.1560 | 0.0004 | 0.1594 | 0.0001 |
| 99 | Cb_IL_DAN_B-Cb_CL_FPN_B | 0.1647 | 0.0002 | 0.1680 | 0.0002 | 0.1647 | 0.0006 | 0.1859 | 0.0001 |
| 100 | Cb_IL_DAN_B-Cb_CL_DMN_A | 0.2106 | 0.0001 | 0.2050 | 0.0001 | 0.2114 | 0.0001 | 0.2295 | 0.0001 |
| 101 | Cb_IL_DAN_B-Cb_CL_DMN_B | 0.1865 | 0.0001 | 0.1859 | 0.0002 | 0.1917 | 0.0001 | 0.2028 | 0.0001 |
| 102 | Cb_IL_VAN_A-Cb_CL_FPN_B | 0.2469 | 0.0001 | 0.2487 | 0.0001 | 0.2376 | 0.0001 | 0.2554 | 0.0001 |
| 103 | Cb_IL_VAN_A-Cb_CL_TPN | -0.1215 | 0.0001 | -0.1187 | 0.0002 | -0.1048 | 0.0002 | -0.1122 | 0.0004 |
| 104 | Cb_IL_VAN_A-Cb_CL_DMN_A | 0.2571 | 0.0001 | 0.2501 | 0.0001 | 0.2556 | 0.0002 | 0.2448 | 0.0001 |
| 105 | Cb_IL_VAN_A-Cb_CL_DMN_B | 0.2586 | 0.0001 | 0.2540 | 0.0001 | 0.2667 | 0.0001 | 0.2525 | 0.0001 |
| 106 | Cb_IL_VAN_B-Cb_CL_FPN_B | 0.1802 | 0.0002 | 0.1824 | 0.0003 | 0.1762 | 0.0003 | 0.1826 | 0.0003 |
| 107 | Cb_IL_VAN_B-Cb_CL_TPN | -0.1236 | 0.0005 | -0.1261 | 0.0004 | -0.1015 | 0.0026 | -0.1230 | 0.0008 |
| 108 | Cb_IL_VAN_B-Cb_CL_DMN_A | 0.1728 | 0.0007 | 0.1770 | 0.0007 | 0.2191 | 0.0001 | 0.1951 | 0.0003 |
| 109 | Cb_IL_VAN_B-Cb_CL_DMN_B | 0.2400 | 0.0001 | 0.2435 | 0.0001 | 0.2519 | 0.0001 | 0.2540 | 0.0001 |
| 110 | Cb_IL_LN_A-Cb_CL_DAN_B | 0.1658 | 0.0001 | 0.1657 | 0.0002 | 0.1533 | 0.0001 | 0.1448 | 0.0001 |
| 111 | Cb_IL_LN_A-Cb_CL_VAN_A | 0.1835 | 0.0001 | 0.1765 | 0.0001 | 0.1845 | 0.0003 | 0.1657 | 0.0008 |
| 112 | Cb_IL_LN_A-Cb_CL_FPN_A | 0.1883 | 0.0001 | 0.1816 | 0.0001 | 0.2025 | 0.0001 | 0.1919 | 0.0001 |
| 113 | Cb_IL_LN_A-Cb_CL_FPN_B | 0.2208 | 0.0001 | 0.2155 | 0.0001 | 0.2210 | 0.0001 | 0.2060 | 0.0001 |
| 114 | Cb_IL_LN_A-Cb_CL_DMN_A | 0.1809 | 0.0001 | 0.1767 | 0.0002 | 0.1694 | 0.0005 | 0.1424 | 0.0020 |
| 115 | Cb_IL_LN_A-Cb_CL_DMN_B | 0.2020 | 0.0001 | 0.1924 | 0.0001 | 0.2043 | 0.0001 | 0.1788 | 0.0001 |
| 116 | Cb_IL_FPN_A-Cb_CL_DMN_B | 0.1816 | 0.0003 | 0.1780 | 0.0007 | 0.2194 | 0.0001 | 0.1900 | 0.0006 |
| 117 | Cb_IL_FPN_B-Cb_CL_SMN_B | 0.1753 | 0.0001 | 0.1803 | 0.0001 | 0.1778 | 0.0001 | 0.1858 | 0.0001 |
| 118 | Cb_IL_FPN_B-Cb_CL_VAN_A | 0.1717 | 0.0004 | 0.1658 | 0.0005 | 0.1801 | 0.0003 | 0.1908 | 0.0003 |
| 119 | Cb_IL_TPN-Cb_CL_VN_B | 0.1199 | 0.0001 | 0.1177 | 0.0003 | 0.1194 | 0.0001 | 0.1198 | 0.0001 |
| 120 | Cb_IL_DMN_A-Cb_CL_VN_B | 0.1519 | 0.0001 | 0.1585 | 0.0001 | 0.1715 | 0.0001 | 0.1499 | 0.0001 |
| 121 | Cb_IL_DMN_A-Cb_CL_SMN_B | 0.1352 | 0.0008 | 0.1453 | 0.0004 | 0.1567 | 0.0002 | 0.1448 | 0.0005 |
| 122 | Cb_IL_DMN_A-Cb_CL_DAN_A | 0.1498 | 0.0001 | 0.1465 | 0.0001 | 0.1606 | 0.0001 | 0.1566 | 0.0001 |
| 123 | Cb_IL_DMN_A-Cb_CL_DAN_B | 0.2288 | 0.0001 | 0.2383 | 0.0001 | 0.2637 | 0.0001 | 0.2389 | 0.0001 |
| 124 | Cb_IL_DMN_A-Cb_CL_VAN_A | 0.2346 | 0.0001 | 0.2422 | 0.0001 | 0.2647 | 0.0001 | 0.2434 | 0.0001 |
| 125 | Cb_IL_DMN_A-Cb_CL_FPN_A | 0.1821 | 0.0001 | 0.1786 | 0.0001 | 0.1863 | 0.0002 | 0.1782 | 0.0001 |
| 126 | Ce_IL_SMN_B-Cb_C_VN_B | -0.1240 | 0.0006 | -0.1177 | 0.0011 | -0.1245 | 0.0007 | -0.1262 | 0.0003 |
| 127 | Ce_IL_SMN_B-Cb_C_SMN_B | -0.1802 | 0.0003 | -0.1799 | 0.0004 | -0.1732 | 0.0003 | -0.1757 | 0.0003 |
| 128 | Ce_IL_SMN_B-Cb_C_VAN_A | -0.1764 | 0.0004 | -0.1740 | 0.0004 | -0.1946 | 0.0001 | -0.1861 | 0.0003 |
| 129 | Ce_IL_DAN_B-Cb_C_DAN_B | -0.1435 | 0.0008 | -0.1488 | 0.0009 | -0.1970 | 0.0001 | -0.1534 | 0.0012 |
| 130 | Ce_IL_VAN_A-Cb_C_SMN_A | -0.1536 | 0.0001 | -0.1510 | 0.0005 | -0.1527 | 0.0003 | -0.1428 | 0.0005 |
| 131 | Ce_IL_VAN_A-Cb_C_SMN_B | -0.1715 | 0.0002 | -0.1681 | 0.0001 | -0.1674 | 0.0001 | -0.1687 | 0.0001 |
| 132 | Ce_IL_VAN_A-Cb_C_DAN_A | -0.1295 | 0.0002 | -0.1271 | 0.0004 | -0.1285 | 0.0004 | -0.1260 | 0.0004 |
| 133 | Ce_IL_VAN_A-Cb_C_DAN_B | -0.1742 | 0.0002 | -0.1726 | 0.0003 | -0.2109 | 0.0001 | -0.2130 | 0.0001 |
| 134 | Ce_IL_VAN_A-Cb_C_VAN_A | -0.1919 | 0.0001 | -0.1898 | 0.0001 | -0.2023 | 0.0001 | -0.1897 | 0.0001 |
| 135 | Ce_IL_LN_A-Cb_C_DAN_B | 0.1281 | 0.0007 | 0.1217 | 0.0013 | 0.0823 | 0.0156 | 0.1027 | 0.0051 |
| 136 | Ce_IL_LN_B-Cb_C_SMN_A | 0.1468 | 0.0002 | 0.1504 | 0.0002 | 0.0996 | 0.0047 | 0.1373 | 0.0004 |
| 137 | Ce_IL_FPN_A-Cb_C_FPN_A | -0.2602 | 0.0001 | -0.2637 | 0.0001 | -0.2780 | 0.0001 | -0.2681 | 0.0001 |
| 138 | Ce_IL_DMN_C-Cb_C_VAN_A | 0.1364 | 0.0004 | 0.1383 | 0.0002 | 0.1359 | 0.0003 | 0.1295 | 0.0003 |
| 139 | Ce_IL_VN_A-Cb_I_FPN_C | 0.1313 | 0.0004 | 0.1323 | 0.0007 | 0.0865 | 0.0127 | 0.1280 | 0.0006 |
| 140 | Ce_IL_VN_B-Cb_I_DAN_B | 0.1401 | 0.0005 | 0.1590 | 0.0003 | 0.1359 | 0.0004 | 0.1445 | 0.0001 |
| 141 | Ce_IL_SMN_B-Cb_I_VAN_A | -0.1649 | 0.0004 | -0.1633 | 0.0008 | -0.1581 | 0.0006 | -0.1636 | 0.0004 |
| 142 | Ce_IL_SMN_B-Cb_I_LN_A | -0.1493 | 0.0002 | -0.1360 | 0.0011 | -0.1484 | 0.0006 | -0.1422 | 0.0006 |
| 143 | Ce_IL_VAN_A-Cb_I_DAN_B | -0.1921 | 0.0002 | -0.1952 | 0.0001 | -0.2030 | 0.0002 | -0.2025 | 0.0001 |
| 144 | Ce_IL_VAN_A-Cb_I_VAN_A | -0.1986 | 0.0002 | -0.1991 | 0.0004 | -0.1915 | 0.0002 | -0.1917 | 0.0006 |
| 145 | Ce_IL_VAN_A-Cb_I_VAN_B | -0.1959 | 0.0001 | -0.1974 | 0.0003 | -0.2046 | 0.0002 | -0.2176 | 0.0001 |
| 146 | Ce_IL_VAN_A-Cb_I_LN_A | -0.1384 | 0.0006 | -0.1294 | 0.0014 | -0.1363 | 0.0009 | -0.1317 | 0.0013 |
| 147 | Ce_IL_VAN_B-Cb_I_VAN_B | -0.2239 | 0.0001 | -0.2265 | 0.0001 | -0.2296 | 0.0001 | -0.2354 | 0.0001 |
| 148 | Ce_IL_LN_A-Cb_I_DAN_B | 0.1284 | 0.0006 | 0.1269 | 0.0014 | 0.1280 | 0.0010 | 0.1396 | 0.0007 |
| 149 | Ce_IL_LN_B-Cb_I_DAN_B | 0.1429 | 0.0004 | 0.1484 | 0.0002 | 0.0771 | 0.0185 | 0.1243 | 0.0007 |
| 150 | Ce_IL_FPN_A-Cb_I_DAN_B | -0.1260 | 0.0006 | -0.1236 | 0.0004 | -0.1427 | 0.0003 | -0.0990 | 0.0034 |
| 151 | Ce_IL_FPN_A-Cb_I_FPN_A | -0.1763 | 0.0001 | -0.1769 | 0.0001 | -0.1853 | 0.0001 | -0.1737 | 0.0001 |
| 152 | Ce_IL_DMN_C-Cb_I_SMN_A | 0.1450 | 0.0004 | 0.1430 | 0.0005 | 0.1341 | 0.0014 | 0.1331 | 0.0011 |
| 153 | Ce_IL_DMN_C-Cb_I_DAN_B | 0.1329 | 0.0003 | 0.1323 | 0.0004 | 0.1628 | 0.0001 | 0.1434 | 0.0001 |
| 154 | Ce_IL_DMN_B-Cb_I_DAN_B | 0.1373 | 0.0007 | 0.1367 | 0.0007 | 0.1213 | 0.0014 | 0.1409 | 0.0003 |
| 155 | Ce_CL_VN_A-Cb_C_FPN_C | 0.1493 | 0.0001 | 0.1517 | 0.0001 | 0.1125 | 0.0034 | 0.1320 | 0.0006 |
| 156 | Ce_CL_LN_B-Cb_C_DAN_B | 0.1373 | 0.0003 | 0.1365 | 0.0002 | 0.1075 | 0.0054 | 0.1531 | 0.0003 |
| 157 | Ce_CL_FPN_C-Cb_C_FPN_A | 0.1371 | 0.0006 | 0.1348 | 0.0004 | 0.1445 | 0.0003 | 0.1361 | 0.0004 |
| 158 | Ce_CL_FPN_A-Cb_C_DAN_B | -0.1559 | 0.0006 | -0.1539 | 0.0004 | -0.1604 | 0.0004 | -0.1379 | 0.0007 |
| 159 | Ce_CL_DMN_C-Cb_C_FPN_A | 0.1344 | 0.0006 | 0.1359 | 0.0009 | 0.1487 | 0.0001 | 0.1349 | 0.0012 |
| 160 | Ce_CL_DMN_A-Cb_C_FPN_A | 0.1933 | 0.0001 | 0.1982 | 0.0003 | 0.1953 | 0.0002 | 0.1978 | 0.0001 |
| 161 | Ce_CL_DMN_A-Cb_C_DAN_B | 0.1418 | 0.0003 | 0.1419 | 0.0005 | 0.1300 | 0.0010 | 0.1354 | 0.0008 |
| 162 | Ce_CL_DMN_A-Cb_C_VAN_B | 0.1603 | 0.0007 | 0.1707 | 0.0004 | 0.1855 | 0.0001 | 0.1889 | 0.0001 |
| 163 | Ce_CL_DMN_B-Cb_C_FPN_A | 0.1973 | 0.0001 | 0.1981 | 0.0001 | 0.1951 | 0.0001 | 0.2086 | 0.0001 |
| 164 | Ce_CL_DMN_B-Cb_C_DAN_B | 0.1637 | 0.0005 | 0.1633 | 0.0003 | 0.1576 | 0.0006 | 0.1693 | 0.0004 |
| 165 | Ce_CL_DMN_B-Cb_C_VAN_A | 0.1717 | 0.0005 | 0.1775 | 0.0002 | 0.1828 | 0.0001 | 0.1723 | 0.0002 |
| 166 | Ce_CL_DMN_B-Cb_C_VAN_B | 0.1701 | 0.0001 | 0.1817 | 0.0001 | 0.2030 | 0.0001 | 0.1901 | 0.0001 |
| 167 | Ce_CL_VN_B-Cb_C_FPN_A | 0.1776 | 0.0005 | 0.1797 | 0.0003 | 0.1810 | 0.0005 | 0.1674 | 0.0007 |
| 168 | Ce_CL_VN_B-Cb_C_DAN_B | 0.1428 | 0.0004 | 0.1420 | 0.0006 | 0.1339 | 0.0018 | 0.1233 | 0.0033 |
| 169 | Ce_CL_SMN_A-Cb_C_FPN_B | 0.1456 | 0.0006 | 0.1392 | 0.0006 | 0.1277 | 0.0011 | 0.1696 | 0.0001 |
| 170 | Ce_CL_VAN_A-Cb_C_DAN_B | -0.1541 | 0.0008 | -0.1559 | 0.0007 | -0.1559 | 0.0008 | -0.1697 | 0.0007 |
| 171 | Ce_CL_VAN_B-Cb_C_DMN_A | 0.1439 | 0.0005 | 0.1413 | 0.0020 | 0.1414 | 0.0015 | 0.1673 | 0.0003 |
| 172 | Ce_CL_LN_A-Cb_C_DAN_B | 0.1551 | 0.0006 | 0.1474 | 0.0009 | 0.1663 | 0.0004 | 0.1614 | 0.0012 |
| 173 | Ce_CL_SMN_A-Cb_I_FPN_B | 0.1768 | 0.0001 | 0.1749 | 0.0002 | 0.1460 | 0.0007 | 0.1903 | 0.0001 |
| 174 | Ce_CL_SMN_A-Cb_I_DMN_B | 0.1519 | 0.0001 | 0.1526 | 0.0003 | 0.1138 | 0.0030 | 0.1496 | 0.0001 |
| 175 | Ce_CL_DAN_B-Cb_I_FPN_B | 0.1647 | 0.0001 | 0.1589 | 0.0002 | 0.1394 | 0.0010 | 0.1725 | 0.0001 |
| 176 | Ce_CL_DAN_B-Cb_I_DMN_A | 0.1770 | 0.0002 | 0.1746 | 0.0001 | 0.1664 | 0.0003 | 0.1761 | 0.0001 |
| 177 | Ce_CL_DAN_B-Cb_I_DMN_B | 0.1717 | 0.0001 | 0.1685 | 0.0001 | 0.1561 | 0.0006 | 0.1769 | 0.0001 |
| 178 | Ce_CL_VAN_A-Cb_I_SMN_B | -0.1369 | 0.0008 | -0.1364 | 0.0011 | -0.1323 | 0.0020 | -0.1317 | 0.0016 |
| 179 | Ce_CL_VAN_A-Cb_I_DAN_A | -0.1267 | 0.0007 | -0.1233 | 0.0012 | -0.1172 | 0.0017 | -0.1213 | 0.0012 |
| 180 | Ce_CL_VAN_A-Cb_I_VAN_A | -0.1597 | 0.0004 | -0.1608 | 0.0003 | -0.1735 | 0.0002 | -0.1576 | 0.0004 |
| 181 | Ce_CL_VAN_B-Cb_I_FPN_B | 0.1654 | 0.0002 | 0.1549 | 0.0006 | 0.1369 | 0.0015 | 0.1472 | 0.0007 |
| 182 | Ce_CL_VAN_B-Cb_I_DMN_B | 0.1791 | 0.0001 | 0.1655 | 0.0004 | 0.1592 | 0.0005 | 0.1865 | 0.0001 |
| 183 | Ce_CL_LN_A-Cb_I_DAN_B | 0.1533 | 0.0004 | 0.1473 | 0.0002 | 0.1202 | 0.0017 | 0.1496 | 0.0003 |
| 184 | Ce_CL_FPN_C-Cb_I_VAN_A | 0.1321 | 0.0004 | 0.1284 | 0.0002 | 0.1275 | 0.0004 | 0.1359 | 0.0002 |
| 185 | Ce_CL_FPN_C-Cb_I_FPN_A | 0.2070 | 0.0002 | 0.2074 | 0.0001 | 0.2037 | 0.0001 | 0.2024 | 0.0001 |
| 186 | Ce_CL_FPN_C-Cb_I_FPN_B | 0.1679 | 0.0002 | 0.1692 | 0.0002 | 0.1699 | 0.0001 | 0.1704 | 0.0001 |
| 187 | Ce_CL_FPN_C-Cb_I_DMN_B | 0.1777 | 0.0002 | 0.1749 | 0.0002 | 0.1879 | 0.0003 | 0.1760 | 0.0003 |
| 188 | Ce_CL_FPN_A-Cb_I_DAN_B | -0.1408 | 0.0004 | -0.1418 | 0.0004 | -0.1704 | 0.0001 | -0.1320 | 0.0010 |
| 189 | Ce_CL_DMN_A-Cb_I_VAN_A | 0.1361 | 0.0005 | 0.1382 | 0.0005 | 0.1264 | 0.0009 | 0.1172 | 0.0014 |
| 190 | Ce_CL_DMN_A-Cb_I_FPN_A | 0.1670 | 0.0003 | 0.1749 | 0.0002 | 0.1653 | 0.0005 | 0.1649 | 0.0003 |
| 191 | Ce_CL_DMN_A-Cb_I_TPN | 0.0995 | 0.0004 | 0.1052 | 0.0005 | 0.0951 | 0.0009 | 0.0979 | 0.0006 |
| 192 | Ce_CL_DMN_B-Cb_I_VN_B | 0.1111 | 0.0005 | 0.1153 | 0.0002 | 0.1097 | 0.0004 | 0.1139 | 0.0004 |
| 193 | Ce_CL_DMN_B-Cb_I_FPN_A | 0.1403 | 0.0005 | 0.1415 | 0.0012 | 0.1085 | 0.0058 | 0.1354 | 0.0008 |

Original, original pipeline includes standard preprocessing without the specific modifications tested here. Scrubbing, motion scrubbing with framewise displacement (FD) thresholding at 0.2 mm; GSR, Global Signal Regression; No Lag, without hemodynamic lag correction; Δ, Delta (patients - HC); Ce, cerebrum; Cb, cerebellum; IL, ipsilesional; CL, contralesional; I, ipsilateral; C, contralateral; VN, visual network; SMN, somatomotor network; DAN, dorsal attention network; VAN, ventral attention network; LN, limbic network; FPN, frontoparietal network; TPN, temporal parietal network; DMN, default mode network.

**Table S6. Significant Spearman correlation between connectivity metrics and clinical variables in Stroke.**

|  | **Connections** | **Spearman (Unadjusted)** | | **Spearman (adjusted)** | | **Notes** |
| --- | --- | --- | --- | --- | --- | --- |
|  |  | ***r value*** | ***p value*** | ***r value*** | ***p value*** |  |
| **7 modules** |  |  |  |  |  |  |
|  | **Lesion size** |  |  |  |  |  |
|  | Ce_IL_SMN-Ce_CL_SMN | -0.3380 | 0.0059 | -0.3426 | 0.0074 | remains sig. |
|  | Ce_IL_SMN-Cb_C_SMN | -0.4516 | 0.0002 | -0.4457 | 0.0004 | remains sig. |
|  | Ce_IL_VAN-Cb_C_VAN | -0.3566 | 0.0035 | -0.4051 | 0.0013 | remains sig. |
|  | Ce_IL_SMN-Cb_I_SMN | -0.4433 | 0.0002 | -0.4298 | 0.0006 | remains sig. |
|  | Ce_IL_SMN-Cb_I_VAN | -0.3850 | 0.0015 | -0.3581 | 0.0050 | remains sig. |
|  | **NIHSS score** |  |  |  |  |  |
|  | Cb_IL_VN-Cb_CL_VN | 0.3322 | 0.0069 | 0.3470 | 0.0066 | remains sig. |
| **17 modules** | |  |  |  |  |  |
|  | **Lesion size** |  |  |  |  |  |
|  | Ce_IL_DAN A-Ce_IL_TPN | 0.3761 | 0.0020 | 0.3462 | 0.0067 | remains sig. |
|  | Ce_IL_SMN B-Cb_C_SMN B | -0.4140 | 0.0006 | -0.3860 | 0.0023 | remains sig. |
|  | Ce_IL_VAN A-Cb_C_SMN A | -0.3420 | 0.0053 | -0.3776 | 0.0029 | remains sig. |
|  | Ce_IL_SMN B-Cb_I_VAN A | -0.3848 | 0.0015 | -0.3595 | 0.0048 | remains sig. |
|  | **NIHSS score** |  |  |  |  |  |
|  | Cb_CL_VN B-Cb_CL_FPN B | -0.3734 | 0.0022 | -0.3111 | 0.0155 | trend toward sig. |

This table presents Spearman correlations before and after adjustment for covariates (age, sex, MMSE, education, days since onset), with significance set at *p* < 0.01 (uncorrected). Connections marked "remains sig." sustained significance post-adjustment. Crucially, after applying FDR correction (*p* < 0.05) to all analyses in the "Unadjusted" column, only the two connections highlighted in red remained significant. Ce, cerebrum; Cb, cerebellum; IL, ipsilesional; CL, contralesional; I, ipsilateral; C, contralateral; NIHSS, National Institutes of Health Stroke Scale; VN, visual network; SMN, somatomotor network; DAN, dorsal attention network; VAN, ventral attention network; FPN, frontoparietal network; TPN, temporal parietal network.

**Table S7. Exploratory Spearman correlations between functional connections and clinical variables.**

|  | **connections** | **lesion size** | | | | | **NIHSS score** | | | | |
| --- | --- | --- | --- | --- | --- | --- | --- | --- | --- | --- | --- |
|  |  | ***r*** | **95% CI** | | ***p*** | ***p_*FDR** | ***r*** | **95% CI** | | ***p*** | ***p_*FDR** |
|  |  |  | **lower** | **upper** |  |  |  | **lower** | **upper** |  |  |
| **7 modules** | |  |  |  |  |  |  |  |  |  |  |
| 1 | Ce_IL_VN-Ce_IL_DAN | -0.0342 | -0.2828 | 0.2245 | 0.7869 | 0.8942 | -0.0414 | -0.2767 | 0.2025 | 0.7435 | 1.0000 |
| 2 | Ce_IL_SMN-Ce_IL_DAN | 0.2584 | 0.0102 | 0.4610 | 0.0377 | 0.3139 | -0.0228 | -0.2706 | 0.2240 | 0.8568 | 1.0000 |
| 3 | Ce_IL_SMN-Ce_IL_LN | 0.0139 | -0.2529 | 0.2720 | 0.9126 | 0.9813 | 0.0537 | -0.2284 | 0.3022 | 0.6708 | 1.0000 |
| 4 | Ce_IL_DAN-Ce_IL_DMN | 0.0849 | -0.1727 | 0.3231 | 0.5013 | 0.7712 | -0.1438 | -0.4138 | 0.1262 | 0.2532 | 1.0000 |
| 5 | Ce_IL_VAN-Ce_IL_DMN | 0.0083 | -0.2458 | 0.2917 | 0.9474 | 0.9972 | -0.1692 | -0.4391 | 0.1025 | 0.1780 | 1.0000 |
| 6 | Ce_IL_FPN-Ce_IL_DMN | 0.1322 | -0.1102 | 0.3571 | 0.2937 | 0.6384 | 0.1434 | -0.1061 | 0.3814 | 0.2544 | 1.0000 |
| 7 | Ce_CL_VN-Ce_CL_DAN | -0.0540 | -0.3109 | 0.2076 | 0.6692 | 0.8580 | -0.0752 | -0.3365 | 0.1968 | 0.5515 | 1.0000 |
| 8 | Ce_CL_VN-Ce_CL_VAN | -0.0581 | -0.2974 | 0.2120 | 0.6455 | 0.8494 | 0.1784 | -0.0835 | 0.4214 | 0.1550 | 1.0000 |
| 9 | Ce_CL_VN-Ce_CL_FPN | 0.1119 | -0.0866 | 0.3183 | 0.3747 | 0.6460 | 0.0399 | -0.2240 | 0.2856 | 0.7525 | 1.0000 |
| 10 | Ce_CL_FPN-Ce_CL_DMN | 0.1282 | -0.1147 | 0.3685 | 0.3090 | 0.6437 | 0.1636 | -0.0904 | 0.4009 | 0.1928 | 1.0000 |
| 11 | Ce_IL_SMN-Ce_CL_SMN | -0.3380 | -0.5706 | -0.0817 | 0.0059 | 0.1177 | -0.0312 | -0.2495 | 0.2069 | 0.8052 | 1.0000 |
| 12 | Ce_IL_DAN-Ce_CL_VN | 0.0183 | -0.2348 | 0.2566 | 0.8850 | 0.9620 | 0.1008 | -0.1402 | 0.3332 | 0.4244 | 1.0000 |
| 13 | Ce_IL_DAN-Ce_CL_DAN | -0.3036 | -0.5128 | -0.0676 | 0.0140 | 0.1994 | 0.0402 | -0.1995 | 0.3017 | 0.7504 | 1.0000 |
| 14 | Ce_IL_DAN-Ce_CL_FPN | -0.1387 | -0.3599 | 0.0995 | 0.2704 | 0.6289 | -0.0238 | -0.2822 | 0.2327 | 0.8505 | 1.0000 |
| 15 | Ce_IL_VAN-Ce_CL_DAN | 0.1431 | -0.1199 | 0.3922 | 0.2556 | 0.6390 | 0.0946 | -0.1673 | 0.3340 | 0.4534 | 1.0000 |
| 16 | Ce_IL_VAN-Ce_CL_VAN | -0.2490 | -0.4780 | 0.0088 | 0.0455 | 0.2844 | -0.0647 | -0.3170 | 0.1863 | 0.6088 | 1.0000 |
| 17 | Ce_IL_VAN-Ce_CL_FPN | 0.1394 | -0.0823 | 0.3686 | 0.2681 | 0.6384 | 0.0090 | -0.2391 | 0.2555 | 0.9432 | 1.0000 |
| 18 | Ce_IL_DMN-Ce_CL_DAN | 0.0786 | -0.1771 | 0.3025 | 0.5339 | 0.7627 | 0.0592 | -0.1738 | 0.2987 | 0.6393 | 1.0000 |
| 19 | Ce_IL_DMN-Ce_CL_VAN | 0.0793 | -0.2132 | 0.3120 | 0.5301 | 0.7683 | -0.0730 | -0.3563 | 0.2065 | 0.5635 | 1.0000 |
| 20 | Ce_IL_DMN-Ce_CL_FPN | 0.0396 | -0.2159 | 0.2887 | 0.7540 | 0.8768 | 0.2163 | -0.0438 | 0.4402 | 0.0835 | 1.0000 |
| 21 | Cb_IL_VN-Cb_IL_DAN | 0.2469 | 0.0001 | 0.4735 | 0.0474 | 0.2790 | 0.1411 | -0.1181 | 0.3755 | 0.2624 | 1.0000 |
| 22 | Cb_IL_VN-Cb_IL_FPN | 0.0360 | -0.2452 | 0.3097 | 0.7756 | 0.8915 | -0.0758 | -0.3278 | 0.1905 | 0.5485 | 1.0000 |
| 23 | Cb_IL_SMN-Cb_IL_LN | 0.1212 | -0.1560 | 0.3599 | 0.3364 | 0.6346 | -0.0006 | -0.2853 | 0.2752 | 0.9965 | 0.9965 |
| 24 | Cb_IL_DAN-Cb_IL_LN | -0.1422 | -0.3960 | 0.1191 | 0.2586 | 0.6308 | 0.0142 | -0.2401 | 0.2659 | 0.9108 | 1.0000 |
| 25 | Cb_IL_DAN-Cb_IL_DMN | -0.0452 | -0.2906 | 0.2072 | 0.7210 | 0.8901 | -0.2151 | -0.4380 | 0.0473 | 0.0853 | 1.0000 |
| 26 | Cb_IL_VAN-Cb_IL_LN | 0.1315 | -0.1137 | 0.3602 | 0.2966 | 0.6311 | 0.0059 | -0.2421 | 0.2409 | 0.9629 | 0.9825 |
| 27 | Cb_IL_VAN-Cb_IL_FPN | 0.1333 | -0.1412 | 0.3708 | 0.2897 | 0.6437 | 0.0175 | -0.2221 | 0.2638 | 0.8902 | 1.0000 |
| 28 | Cb_IL_VAN-Cb_IL_DMN | 0.1222 | -0.1415 | 0.3566 | 0.3322 | 0.6389 | -0.0291 | -0.2626 | 0.2185 | 0.8182 | 1.0000 |
| 29 | Cb_IL_LN-Cb_IL_FPN | 0.0279 | -0.2360 | 0.2788 | 0.8256 | 0.9277 | -0.0492 | -0.3052 | 0.2178 | 0.6971 | 1.0000 |
| 30 | Cb_IL_LN-Cb_IL_DMN | 0.1799 | -0.0693 | 0.4033 | 0.1516 | 0.4593 | -0.0349 | -0.2808 | 0.2102 | 0.7825 | 1.0000 |
| 31 | Cb_IL_FPN-Cb_IL_DMN | 0.1098 | -0.1355 | 0.3388 | 0.3838 | 0.6504 | -0.0791 | -0.3561 | 0.1764 | 0.5311 | 1.0000 |
| 32 | Cb_CL_VN-Cb_CL_LN | -0.1345 | -0.3403 | 0.0825 | 0.2855 | 0.6488 | -0.2300 | -0.4811 | 0.0254 | 0.0653 | 1.0000 |
| 33 | Cb_CL_VN-Cb_CL_DMN | 0.0499 | -0.1962 | 0.2780 | 0.6929 | 0.8662 | -0.2282 | -0.4544 | 0.0293 | 0.0676 | 1.0000 |
| 34 | Cb_CL_SMN-Cb_CL_DAN | 0.1822 | -0.0585 | 0.4248 | 0.1464 | 0.4575 | -0.0094 | -0.2847 | 0.2542 | 0.9406 | 1.0000 |
| 35 | Cb_CL_SMN-Cb_CL_FPN | 0.2400 | 0.0123 | 0.4664 | 0.0542 | 0.2851 | -0.0066 | -0.2805 | 0.2556 | 0.9586 | 0.9985 |
| 36 | Cb_CL_SMN-Cb_CL_DMN | 0.1681 | -0.0761 | 0.3916 | 0.1806 | 0.5312 | -0.0723 | -0.3274 | 0.1736 | 0.5671 | 1.0000 |
| 37 | Cb_CL_DAN-Cb_CL_LN | -0.1210 | -0.3553 | 0.1268 | 0.3369 | 0.6238 | -0.1500 | -0.3764 | 0.0785 | 0.2331 | 1.0000 |
| 38 | Cb_CL_DAN-Cb_CL_DMN | -0.0080 | -0.2766 | 0.2617 | 0.9494 | 0.9890 | -0.0865 | -0.3304 | 0.1410 | 0.4934 | 1.0000 |
| 39 | Cb_CL_VAN-Cb_CL_LN | 0.0696 | -0.1689 | 0.2876 | 0.5817 | 0.8079 | 0.0260 | -0.2252 | 0.2802 | 0.8373 | 1.0000 |
| 40 | Cb_CL_VAN-Cb_CL_FPN | 0.1276 | -0.1440 | 0.3739 | 0.3110 | 0.6347 | -0.0800 | -0.3090 | 0.1518 | 0.5265 | 1.0000 |
| 41 | Cb_CL_VAN-Cb_CL_DMN | 0.1259 | -0.1245 | 0.3705 | 0.3176 | 0.6351 | -0.0877 | -0.3063 | 0.1364 | 0.4872 | 1.0000 |
| 42 | Cb_CL_LN-Cb_CL_FPN | 0.0422 | -0.2013 | 0.2820 | 0.7387 | 0.8794 | -0.1126 | -0.3626 | 0.1403 | 0.3720 | 1.0000 |
| 43 | Cb_CL_LN-Cb_CL_DMN | 0.0138 | -0.2373 | 0.2692 | 0.9130 | 0.9713 | -0.0694 | -0.3159 | 0.1794 | 0.5830 | 1.0000 |
| 44 | Cb_IL_VN-Cb_CL_VN | 0.1168 | -0.1303 | 0.3575 | 0.3541 | 0.6212 | 0.3322 | 0.1216 | 0.5063 | 0.0069 | 0.6858 |
| 45 | Cb_IL_VN-Cb_CL_LN | 0.0619 | -0.1912 | 0.3280 | 0.6241 | 0.8433 | 0.1073 | -0.1391 | 0.3583 | 0.3949 | 1.0000 |
| 46 | Cb_IL_VN-Cb_CL_DMN | 0.0813 | -0.1639 | 0.3258 | 0.5195 | 0.7754 | 0.0863 | -0.1608 | 0.3286 | 0.4941 | 1.0000 |
| 47 | Cb_IL_SMN-Cb_CL_DMN | 0.1448 | -0.1144 | 0.3647 | 0.2499 | 0.6408 | -0.0853 | -0.3000 | 0.1468 | 0.4995 | 1.0000 |
| 48 | Cb_IL_DAN-Cb_CL_DMN | 0.0412 | -0.2156 | 0.2795 | 0.7444 | 0.8758 | -0.0859 | -0.3170 | 0.1364 | 0.4963 | 1.0000 |
| 49 | Cb_IL_VAN-Cb_CL_FPN | 0.2752 | 0.0331 | 0.4710 | 0.0265 | 0.2943 | 0.0894 | -0.1649 | 0.3382 | 0.4789 | 1.0000 |
| 50 | Cb_IL_VAN-Cb_CL_DMN | 0.2375 | -0.0021 | 0.4604 | 0.0568 | 0.2839 | 0.0448 | -0.1901 | 0.2701 | 0.7229 | 1.0000 |
| 51 | Cb_IL_LN-Cb_CL_VN | 0.0189 | -0.2407 | 0.2588 | 0.8811 | 0.9682 | -0.0494 | -0.3001 | 0.2074 | 0.6957 | 1.0000 |
| 52 | Cb_IL_LN-Cb_CL_DAN | -0.1067 | -0.3611 | 0.1618 | 0.3975 | 0.6517 | -0.0652 | -0.3041 | 0.1730 | 0.6061 | 1.0000 |
| 53 | Cb_IL_LN-Cb_CL_VAN | 0.1521 | -0.0910 | 0.3745 | 0.2265 | 0.6290 | 0.0952 | -0.1628 | 0.3825 | 0.4504 | 1.0000 |
| 54 | Cb_IL_LN-Cb_CL_FPN | 0.0900 | -0.1785 | 0.3230 | 0.4760 | 0.7437 | -0.0183 | -0.2487 | 0.1892 | 0.8851 | 1.0000 |
| 55 | Cb_IL_LN-Cb_CL_DMN | 0.1030 | -0.1531 | 0.3464 | 0.4141 | 0.6574 | -0.0447 | -0.2747 | 0.1983 | 0.7237 | 1.0000 |
| 56 | Cb_IL_FPN-Cb_CL_LN | 0.1961 | -0.0444 | 0.4057 | 0.1175 | 0.4050 | 0.0535 | -0.1991 | 0.2898 | 0.6724 | 1.0000 |
| 57 | Cb_IL_DMN-Cb_CL_VN | 0.1545 | -0.1030 | 0.3880 | 0.2192 | 0.6264 | -0.1392 | -0.3786 | 0.1363 | 0.2688 | 1.0000 |
| 58 | Cb_IL_DMN-Cb_CL_SMN | 0.2207 | 0.0052 | 0.4315 | 0.0773 | 0.3514 | 0.0959 | -0.1625 | 0.3222 | 0.4472 | 1.0000 |
| 59 | Cb_IL_DMN-Cb_CL_DAN | 0.1246 | -0.1056 | 0.3340 | 0.3228 | 0.6330 | 0.0589 | -0.1641 | 0.2912 | 0.6409 | 1.0000 |
| 60 | Cb_IL_DMN-Cb_CL_VAN | 0.2105 | -0.0140 | 0.4384 | 0.0924 | 0.4019 | 0.0875 | -0.1465 | 0.3232 | 0.4880 | 1.0000 |
| 61 | Ce_IL_SMN-Cb_C_VN | -0.2103 | -0.4462 | 0.0434 | 0.0926 | 0.3859 | 0.1018 | -0.1613 | 0.3558 | 0.4195 | 1.0000 |
| 62 | Ce_IL_SMN-Cb_C_SMN | -0.4516 | -0.6124 | -0.2595 | 0.0002 | 0.0160 | -0.0102 | -0.2567 | 0.2494 | 0.9354 | 1.0000 |
| 63 | Ce_IL_SMN-Cb_C_VAN | -0.3160 | -0.5148 | -0.0907 | 0.0103 | 0.1723 | -0.0605 | -0.3299 | 0.1832 | 0.6320 | 1.0000 |
| 64 | Ce_IL_DAN-Cb_C_DAN | -0.2262 | -0.4235 | -0.0087 | 0.0700 | 0.3334 | 0.0050 | -0.2338 | 0.2294 | 0.9682 | 0.9780 |
| 65 | Ce_IL_VAN-Cb_C_SMN | -0.2933 | -0.4914 | -0.0641 | 0.0177 | 0.2219 | -0.0383 | -0.2971 | 0.2160 | 0.7621 | 1.0000 |
| 66 | Ce_IL_VAN-Cb_C_DAN | -0.1830 | -0.3913 | 0.0449 | 0.1446 | 0.4664 | -0.0448 | -0.2820 | 0.1970 | 0.7231 | 1.0000 |
| 67 | Ce_IL_VAN-Cb_C_VAN | -0.3566 | -0.5664 | -0.1250 | 0.0035 | 0.0887 | -0.1005 | -0.3402 | 0.1386 | 0.4257 | 1.0000 |
| 68 | Ce_IL_FPN-Cb_C_DAN | -0.2577 | -0.4941 | -0.0076 | 0.0382 | 0.2939 | -0.1627 | -0.3959 | 0.0736 | 0.1954 | 1.0000 |
| 69 | Ce_IL_FPN-Cb_C_FPN | -0.2563 | -0.4681 | -0.0420 | 0.0393 | 0.2622 | -0.0910 | -0.3401 | 0.1341 | 0.4708 | 1.0000 |
| 70 | Ce_IL_DMN-Cb_C_VN | -0.2401 | -0.4548 | -0.0214 | 0.0540 | 0.3002 | -0.1225 | -0.3449 | 0.1164 | 0.3311 | 1.0000 |
| 71 | Ce_IL_DMN-Cb_C_DAN | 0.0042 | -0.2398 | 0.2650 | 0.9734 | 0.9832 | -0.0237 | -0.2834 | 0.2288 | 0.8515 | 1.0000 |
| 72 | Ce_IL_VN-Cb_I_DAN | -0.1179 | -0.3556 | 0.1176 | 0.3495 | 0.6241 | -0.0809 | -0.3246 | 0.1600 | 0.5218 | 1.0000 |
| 73 | Ce_IL_SMN-Cb_I_VN | -0.2615 | -0.4699 | -0.0251 | 0.0354 | 0.3215 | -0.0633 | -0.2976 | 0.2053 | 0.6163 | 1.0000 |
| 74 | Ce_IL_SMN-Cb_I_SMN | -0.4433 | -0.6092 | -0.2579 | 0.0002 | 0.0108 | 0.0083 | -0.2396 | 0.2389 | 0.9478 | 0.9977 |
| 75 | Ce_IL_SMN-Cb_I_VAN | -0.3850 | -0.5698 | -0.1752 | 0.0015 | 0.0513 | -0.0245 | -0.2939 | 0.2312 | 0.8462 | 1.0000 |
| 76 | Ce_IL_VAN-Cb_I_VN | -0.2025 | -0.4399 | 0.0606 | 0.1058 | 0.4068 | 0.0603 | -0.1612 | 0.2747 | 0.6334 | 1.0000 |
| 77 | Ce_IL_VAN-Cb_I_DAN | -0.2563 | -0.4493 | -0.0262 | 0.0393 | 0.2807 | -0.0180 | -0.2554 | 0.2373 | 0.8866 | 1.0000 |
| 78 | Ce_IL_VAN-Cb_I_VAN | -0.1973 | -0.4370 | 0.0480 | 0.1152 | 0.4114 | 0.0395 | -0.2219 | 0.3115 | 0.7548 | 1.0000 |
| 79 | Ce_IL_VAN-Cb_I_LN | -0.2697 | -0.5090 | -0.0097 | 0.0298 | 0.2982 | -0.1511 | -0.3833 | 0.1007 | 0.2296 | 1.0000 |
| 80 | Ce_IL_LN-Cb_I_SMN | -0.1993 | -0.4485 | 0.0579 | 0.1115 | 0.4129 | 0.0647 | -0.1835 | 0.3170 | 0.6085 | 1.0000 |
| 81 | Ce_IL_LN-Cb_I_DAN | -0.0448 | -0.2742 | 0.2100 | 0.7229 | 0.8816 | -0.0063 | -0.2564 | 0.2353 | 0.9602 | 0.9899 |
| 82 | Ce_IL_DMN-Cb_I_DAN | 0.0051 | -0.2308 | 0.2364 | 0.9678 | 0.9875 | -0.0894 | -0.3250 | 0.1558 | 0.4789 | 1.0000 |
| 83 | Ce_IL_DMN-Cb_I_VAN | 0.0819 | -0.1898 | 0.3403 | 0.5168 | 0.7830 | -0.0315 | -0.2693 | 0.2112 | 0.8030 | 1.0000 |
| 84 | Ce_CL_VN-Cb_C_DAN | 0.0627 | -0.1899 | 0.3007 | 0.6196 | 0.8488 | 0.0092 | -0.2682 | 0.2662 | 0.9421 | 1.0000 |
| 85 | Ce_CL_SMN-Cb_C_VN | 0.1063 | -0.1506 | 0.3432 | 0.3992 | 0.6438 | 0.0162 | -0.2178 | 0.2612 | 0.8978 | 1.0000 |
| 86 | Ce_CL_SMN-Cb_C_FPN | 0.0611 | -0.2214 | 0.3058 | 0.6287 | 0.8383 | 0.0658 | -0.1936 | 0.3392 | 0.6024 | 1.0000 |
| 87 | Ce_CL_VAN-Cb_C_DAN | -0.1473 | -0.3929 | 0.0998 | 0.2415 | 0.6527 | -0.0766 | -0.3231 | 0.1787 | 0.5440 | 1.0000 |
| 88 | Ce_CL_VAN-Cb_C_VAN | -0.0231 | -0.2573 | 0.2147 | 0.8551 | 0.9501 | 0.0175 | -0.2441 | 0.2768 | 0.8899 | 1.0000 |
| 89 | Ce_CL_LN-Cb_C_SMN | -0.2034 | -0.4435 | 0.0492 | 0.1042 | 0.4168 | -0.0278 | -0.2655 | 0.2161 | 0.8257 | 1.0000 |
| 90 | Ce_CL_DMN-Cb_C_DAN | 0.0054 | -0.2562 | 0.2405 | 0.9658 | 0.9957 | -0.1132 | -0.3488 | 0.1212 | 0.3695 | 1.0000 |
| 91 | Ce_CL_DMN-Cb_C_VAN | 0.0427 | -0.2305 | 0.3031 | 0.7354 | 0.8860 | 0.0091 | -0.2330 | 0.2633 | 0.9429 | 1.0000 |
| 92 | Ce_CL_DAN-Cb_I_DMN | 0.1455 | -0.1202 | 0.4074 | 0.2476 | 0.6515 | 0.1698 | -0.0821 | 0.3905 | 0.1764 | 1.0000 |
| 93 | Ce_CL_VAN-Cb_I_DAN | -0.0530 | -0.2710 | 0.1697 | 0.6748 | 0.8541 | -0.1108 | -0.3372 | 0.1125 | 0.3797 | 1.0000 |
| 94 | Ce_CL_VAN-Cb_I_DMN | -0.1086 | -0.3409 | 0.1407 | 0.3893 | 0.6488 | 0.0338 | -0.2308 | 0.2665 | 0.7890 | 1.0000 |
| 95 | Ce_CL_LN-Cb_I_DAN | 0.0007 | -0.2572 | 0.2402 | 0.9959 | 0.9959 | -0.0102 | -0.2294 | 0.2095 | 0.9358 | 1.0000 |
| 96 | Ce_CL_LN-Cb_I_VAN | -0.1189 | -0.3750 | 0.1513 | 0.3457 | 0.6285 | 0.0313 | -0.2191 | 0.2780 | 0.8047 | 1.0000 |
| 97 | Ce_CL_FPN-Cb_I_DMN | 0.0777 | -0.1709 | 0.3066 | 0.5386 | 0.7586 | 0.2060 | -0.0344 | 0.4357 | 0.0996 | 1.0000 |
| 98 | Ce_CL_DMN-Cb_I_VN | 0.0555 | -0.1885 | 0.2798 | 0.6608 | 0.8582 | -0.0242 | -0.2659 | 0.2144 | 0.8483 | 1.0000 |
| 99 | Ce_CL_DMN-Cb_I_DAN | 0.1850 | -0.0745 | 0.4080 | 0.1401 | 0.4671 | 0.0474 | -0.2080 | 0.3090 | 0.7078 | 1.0000 |
| 100 | Ce_CL_DMN-Cb_I_VAN | 0.0793 | -0.1770 | 0.3158 | 0.5299 | 0.7793 | 0.1347 | -0.1149 | 0.3550 | 0.2848 | 1.0000 |
| **17 modules** | |  |  |  |  |  |  |  |  |  |  |
| 1 | Ce_IL_VN_A-Ce_IL_DAN_A | 0.0662 | -0.1983 | 0.3086 | 0.6002 | 0.8275 | 0.1010 | -0.1240 | 0.3198 | 0.4234 | 1.0000 |
| 2 | Ce_IL_VN_A-Ce_IL_DAN_B | -0.0251 | -0.3009 | 0.2325 | 0.8425 | 0.9135 | -0.1108 | -0.3425 | 0.1323 | 0.3798 | 1.0000 |
| 3 | Ce_IL_VN_A-Ce_IL_LN_B | -0.0012 | -0.2542 | 0.2747 | 0.9924 | 1.0000 | 0.0512 | -0.1925 | 0.2970 | 0.6855 | 0.9873 |
| 4 | Ce_IL_VN_A-Ce_IL_DMN_C | -0.1206 | -0.3814 | 0.1304 | 0.3385 | 0.7596 | 0.0900 | -0.1533 | 0.3061 | 0.4757 | 1.0000 |
| 5 | Ce_IL_VN_B-Ce_IL_DAN_A | -0.1485 | -0.3998 | 0.1028 | 0.2377 | 0.7646 | -0.0402 | -0.2635 | 0.1842 | 0.7508 | 0.9925 |
| 6 | Ce_IL_VN_B-Ce_IL_DMN_C | 0.0256 | -0.2132 | 0.2753 | 0.8395 | 0.9154 | -0.0531 | -0.2910 | 0.2065 | 0.6744 | 1.0000 |
| 7 | Ce_IL_SMN_A-Ce_IL_DAN_B | 0.1644 | -0.0861 | 0.4000 | 0.1906 | 0.7663 | 0.1648 | -0.0973 | 0.4046 | 0.1897 | 1.0000 |
| 8 | Ce_IL_SMN_A-Ce_IL_FPN_B | 0.1978 | -0.0595 | 0.4273 | 0.1142 | 0.5955 | -0.0549 | -0.2995 | 0.2005 | 0.6642 | 1.0000 |
| 9 | Ce_IL_SMN_B-Ce_IL_TPN | 0.1503 | -0.1201 | 0.4077 | 0.2322 | 0.7726 | -0.0807 | -0.3333 | 0.1844 | 0.5228 | 1.0000 |
| 10 | Ce_IL_DAN_A-Ce_IL_TPN | 0.3761 | 0.1724 | 0.5414 | 0.0020 | 0.1298 | -0.0411 | -0.3049 | 0.2562 | 0.7454 | 0.9990 |
| 11 | Ce_IL_VAN_A-Ce_IL_TPN | 0.0838 | -0.1937 | 0.3324 | 0.5071 | 0.8022 | -0.1583 | -0.4028 | 0.0994 | 0.2079 | 1.0000 |
| 12 | Ce_IL_VAN_A-Ce_IL_DMN_A | 0.0337 | -0.2296 | 0.3073 | 0.7899 | 0.9074 | -0.0945 | -0.3412 | 0.1790 | 0.4541 | 1.0000 |
| 13 | Ce_IL_VAN_A-Ce_IL_DMN_B | -0.0033 | -0.2761 | 0.2695 | 0.9789 | 1.0000 | -0.1819 | -0.4201 | 0.0806 | 0.1471 | 1.0000 |
| 14 | Ce_IL_VAN_B-Ce_IL_DMN_B | -0.0420 | -0.2843 | 0.1848 | 0.7397 | 0.8923 | 0.0240 | -0.2437 | 0.2965 | 0.8495 | 0.9998 |
| 15 | Ce_IL_FPN_A-Ce_IL_DMN_A | 0.2090 | -0.0310 | 0.4347 | 0.0948 | 0.5382 | 0.1520 | -0.1384 | 0.4079 | 0.2268 | 1.0000 |
| 16 | Ce_IL_DMN_C-Ce_IL_DMN_A | -0.0154 | -0.2418 | 0.2268 | 0.9033 | 0.9475 | -0.1197 | -0.3441 | 0.1318 | 0.3422 | 1.0000 |
| 17 | Ce_CL_VN_A-Ce_CL_DAN_A | 0.1212 | -0.1349 | 0.3823 | 0.3360 | 0.7629 | -0.0753 | -0.3014 | 0.1749 | 0.5509 | 1.0000 |
| 18 | Ce_CL_VN_A-Ce_CL_DAN_B | -0.2527 | -0.4861 | -0.0081 | 0.0422 | 0.3705 | -0.2468 | -0.4746 | -0.0115 | 0.0475 | 1.0000 |
| 19 | Ce_CL_VN_A-Ce_CL_VAN_B | -0.1235 | -0.3240 | 0.0999 | 0.3270 | 0.7603 | -0.0993 | -0.3476 | 0.1547 | 0.4315 | 1.0000 |
| 20 | Ce_CL_VN_A-Ce_CL_FPN_A | -0.0227 | -0.2600 | 0.2318 | 0.8577 | 0.9247 | -0.0990 | -0.3678 | 0.1971 | 0.4327 | 1.0000 |
| 21 | Ce_CL_VN_B-Ce_CL_DAN_A | 0.2194 | -0.0148 | 0.4599 | 0.0791 | 0.4925 | 0.0933 | -0.1740 | 0.3228 | 0.4597 | 1.0000 |
| 22 | Ce_CL_VN_B-Ce_CL_VAN_B | 0.0370 | -0.2092 | 0.2687 | 0.7700 | 0.9006 | 0.1021 | -0.1632 | 0.3567 | 0.4182 | 1.0000 |
| 23 | Ce_CL_VN_B-Ce_CL_FPN_A | 0.0774 | -0.1511 | 0.3133 | 0.5401 | 0.8143 | -0.0089 | -0.2878 | 0.2605 | 0.9439 | 1.0000 |
| 24 | Ce_CL_VAN_B-Ce_CL_DMN_A | 0.1237 | -0.1269 | 0.3446 | 0.3261 | 0.7675 | -0.0447 | -0.3046 | 0.2161 | 0.7239 | 1.0000 |
| 25 | Ce_CL_VAN_B-Ce_CL_DMN_B | 0.0335 | -0.2403 | 0.2876 | 0.7912 | 0.9036 | -0.0923 | -0.3304 | 0.1497 | 0.4648 | 1.0000 |
| 26 | Ce_CL_FPN_A-Ce_CL_DMN_A | 0.1332 | -0.1155 | 0.3655 | 0.2900 | 0.7564 | 0.1633 | -0.0908 | 0.3817 | 0.1937 | 1.0000 |
| 27 | Ce_IL_VN_A-Ce_CL_VN_B | -0.2298 | -0.4534 | 0.0152 | 0.0656 | 0.4868 | 0.1081 | -0.1237 | 0.3471 | 0.3914 | 1.0000 |
| 28 | Ce_IL_SMN_A-Ce_CL_SMN_A | -0.3119 | -0.5351 | -0.0377 | 0.0114 | 0.3673 | -0.0689 | -0.2991 | 0.1631 | 0.5855 | 1.0000 |
| 29 | Ce_IL_SMN_A-Ce_CL_VAN_A | -0.0784 | -0.3106 | 0.1502 | 0.5349 | 0.8129 | -0.0406 | -0.2883 | 0.2016 | 0.7482 | 0.9958 |
| 30 | Ce_IL_SMN_B-Ce_CL_SMN_A | -0.1187 | -0.3561 | 0.1396 | 0.3465 | 0.7513 | -0.0079 | -0.2488 | 0.2169 | 0.9499 | 1.0000 |
| 31 | Ce_IL_SMN_B-Ce_CL_DAN_B | -0.1167 | -0.3474 | 0.1673 | 0.3546 | 0.7358 | 0.0301 | -0.2174 | 0.2940 | 0.8121 | 1.0000 |
| 32 | Ce_IL_DAN_A-Ce_CL_VN_A | 0.0339 | -0.2204 | 0.2875 | 0.7887 | 0.9114 | 0.1069 | -0.1305 | 0.3251 | 0.3965 | 1.0000 |
| 33 | Ce_IL_DAN_A-Ce_CL_FPN_A | -0.0609 | -0.3031 | 0.1851 | 0.6298 | 0.8383 | 0.0981 | -0.1657 | 0.3687 | 0.4367 | 1.0000 |
| 34 | Ce_IL_DAN_B-Ce_CL_VN_A | -0.0712 | -0.3085 | 0.1916 | 0.5731 | 0.8133 | -0.0046 | -0.2655 | 0.2463 | 0.9712 | 1.0000 |
| 35 | Ce_IL_DAN_B-Ce_CL_DAN_B | -0.1507 | -0.3885 | 0.1057 | 0.2309 | 0.7959 | -0.0515 | -0.3128 | 0.2065 | 0.6837 | 0.9921 |
| 36 | Ce_IL_DAN_B-Ce_CL_VAN_B | -0.1505 | -0.3654 | 0.0871 | 0.2315 | 0.7838 | -0.0724 | -0.3471 | 0.1965 | 0.5668 | 1.0000 |
| 37 | Ce_IL_DAN_B-Ce_CL_FPN_A | -0.2084 | -0.4349 | 0.0185 | 0.0957 | 0.5277 | -0.1117 | -0.3515 | 0.1464 | 0.3758 | 1.0000 |
| 38 | Ce_IL_VAN_A-Ce_CL_DAN_B | 0.0482 | -0.1957 | 0.3134 | 0.7031 | 0.8869 | 0.0300 | -0.2192 | 0.2864 | 0.8127 | 0.9990 |
| 39 | Ce_IL_VAN_A-Ce_CL_VAN_A | -0.2533 | -0.4754 | 0.0118 | 0.0418 | 0.3841 | 0.0171 | -0.2591 | 0.2878 | 0.8925 | 0.9957 |
| 40 | Ce_IL_VAN_A-Ce_CL_VAN_B | -0.1070 | -0.3486 | 0.1458 | 0.3963 | 0.7498 | 0.0230 | -0.2385 | 0.2776 | 0.8556 | 0.9947 |
| 41 | Ce_IL_VAN_A-Ce_CL_FPN_A | 0.1613 | -0.0720 | 0.3887 | 0.1993 | 0.7691 | 0.0443 | -0.1964 | 0.2910 | 0.7258 | 1.0000 |
| 42 | Ce_IL_FPN_C-Ce_CL_FPN_C | 0.0420 | -0.2041 | 0.2849 | 0.7397 | 0.8868 | 0.0701 | -0.1716 | 0.3128 | 0.5791 | 1.0000 |
| 43 | Ce_IL_FPN_C-Ce_CL_TPN | 0.0939 | -0.1693 | 0.3674 | 0.4568 | 0.7872 | 0.2003 | -0.0349 | 0.4155 | 0.1096 | 1.0000 |
| 44 | Ce_IL_FPN_A-Ce_CL_FPN_A | -0.0956 | -0.3418 | 0.1703 | 0.4486 | 0.7870 | -0.0800 | -0.3193 | 0.1614 | 0.5265 | 1.0000 |
| 45 | Ce_IL_TPN-Ce_CL_TPN | 0.0166 | -0.2385 | 0.2655 | 0.8954 | 0.9495 | 0.0982 | -0.1331 | 0.3447 | 0.4364 | 1.0000 |
| 46 | Ce_IL_TPN-Ce_CL_DMN_B | -0.1103 | -0.3451 | 0.1324 | 0.3819 | 0.7444 | 0.1091 | -0.1167 | 0.3287 | 0.3872 | 1.0000 |
| 47 | Ce_IL_DMN_C-Ce_CL_VN_A | -0.1888 | -0.4322 | 0.0943 | 0.1320 | 0.6368 | 0.0031 | -0.2441 | 0.2611 | 0.9802 | 1.0000 |
| 48 | Ce_IL_DMN_C-Ce_CL_DMN_C | 0.0723 | -0.2203 | 0.3630 | 0.5672 | 0.8170 | -0.1231 | -0.3511 | 0.1304 | 0.3286 | 1.0000 |
| 49 | Ce_IL_DMN_C-Ce_CL_DMN_A | 0.0735 | -0.2069 | 0.3339 | 0.5608 | 0.8200 | -0.1422 | -0.3646 | 0.1091 | 0.2584 | 1.0000 |
| 50 | Ce_IL_DMN_A-Ce_CL_DAN_B | -0.0002 | -0.2240 | 0.2276 | 0.9990 | 1.0000 | -0.0718 | -0.3132 | 0.1664 | 0.5700 | 1.0000 |
| 51 | Ce_IL_DMN_A-Ce_CL_VAN_B | 0.0890 | -0.1749 | 0.3261 | 0.4807 | 0.8068 | 0.0329 | -0.2272 | 0.3081 | 0.7946 | 1.0000 |
| 52 | Ce_IL_DMN_A-Ce_CL_FPN_A | 0.1437 | -0.1036 | 0.3879 | 0.2533 | 0.7522 | 0.1702 | -0.0876 | 0.3865 | 0.1753 | 1.0000 |
| 53 | Ce_IL_DMN_A-Ce_CL_DMN_B | 0.0484 | -0.1982 | 0.2912 | 0.7018 | 0.8970 | 0.2287 | 0.0131 | 0.4402 | 0.0669 | 1.0000 |
| 54 | Ce_IL_DMN_B-Ce_CL_DAN_B | 0.0703 | -0.2103 | 0.3101 | 0.5780 | 0.8083 | -0.1388 | -0.3968 | 0.1157 | 0.2700 | 1.0000 |
| 55 | Ce_IL_DMN_B-Ce_CL_VAN_B | 0.0872 | -0.1845 | 0.3284 | 0.4897 | 0.8147 | 0.0364 | -0.2109 | 0.3003 | 0.7735 | 0.9887 |
| 56 | Cb_IL_VN_B-Cb_IL_DAN_B | -0.0352 | -0.2540 | 0.1940 | 0.7810 | 0.9080 | 0.0002 | -0.2598 | 0.2532 | 0.9989 | 0.9989 |
| 57 | Cb_IL_VN_B-Cb_IL_FPN_A | 0.1168 | -0.1481 | 0.3721 | 0.3543 | 0.7432 | -0.0173 | -0.2806 | 0.2547 | 0.8914 | 1.0000 |
| 58 | Cb_IL_DAN_B-Cb_IL_LN_A | -0.1322 | -0.3686 | 0.1287 | 0.2938 | 0.7560 | -0.0383 | -0.2907 | 0.2008 | 0.7619 | 0.9935 |
| 59 | Cb_IL_DAN_B-Cb_IL_DMN_A | -0.0009 | -0.2635 | 0.2564 | 0.9941 | 1.0000 | 0.0253 | -0.2264 | 0.2671 | 0.8413 | 0.9962 |
| 60 | Cb_IL_VAN_A-Cb_IL_LN_A | 0.1157 | -0.1270 | 0.3504 | 0.3588 | 0.7368 | 0.0299 | -0.2043 | 0.2673 | 0.8132 | 0.9933 |
| 61 | Cb_IL_VAN_A-Cb_IL_FPN_B | 0.1447 | -0.0807 | 0.3762 | 0.2501 | 0.7543 | 0.0017 | -0.2198 | 0.2312 | 0.9895 | 1.0000 |
| 62 | Cb_IL_VAN_A-Cb_IL_DMN_A | 0.1543 | -0.1012 | 0.4036 | 0.2197 | 0.7852 | 0.1665 | -0.0575 | 0.3874 | 0.1850 | 1.0000 |
| 63 | Cb_IL_VAN_B-Cb_IL_FPN_B | 0.0569 | -0.1845 | 0.2846 | 0.6524 | 0.8565 | -0.1713 | -0.3858 | 0.0869 | 0.1725 | 1.0000 |
| 64 | Cb_IL_VAN_B-Cb_IL_DMN_A | 0.1410 | -0.0858 | 0.3709 | 0.2626 | 0.7678 | 0.0599 | -0.1930 | 0.3246 | 0.6355 | 1.0000 |
| 65 | Cb_IL_VAN_B-Cb_IL_DMN_B | 0.1142 | -0.1322 | 0.3436 | 0.3651 | 0.7340 | -0.0881 | -0.3378 | 0.1421 | 0.4854 | 1.0000 |
| 66 | Cb_IL_LN_A-Cb_IL_FPN_A | -0.0933 | -0.3485 | 0.1790 | 0.4596 | 0.7850 | -0.0673 | -0.3030 | 0.1914 | 0.5942 | 1.0000 |
| 67 | Cb_IL_LN_A-Cb_IL_FPN_B | 0.0483 | -0.2193 | 0.2968 | 0.7023 | 0.8917 | -0.1056 | -0.3490 | 0.1512 | 0.4023 | 1.0000 |
| 68 | Cb_IL_FPN_A-Cb_IL_FPN_B | 0.0956 | -0.1514 | 0.3477 | 0.4488 | 0.7803 | -0.2124 | -0.4596 | 0.0512 | 0.0894 | 1.0000 |
| 69 | Cb_IL_FPN_A-Cb_IL_DMN_A | 0.1224 | -0.1278 | 0.3528 | 0.3313 | 0.7612 | -0.0973 | -0.3492 | 0.1652 | 0.4408 | 1.0000 |
| 70 | Cb_IL_FPN_A-Cb_IL_DMN_B | 0.0966 | -0.1392 | 0.3228 | 0.4439 | 0.7933 | -0.0917 | -0.3155 | 0.1528 | 0.4676 | 1.0000 |
| 71 | Cb_CL_VN_B-Cb_CL_LN_A | -0.0834 | -0.3347 | 0.1542 | 0.5090 | 0.7986 | -0.1513 | -0.3831 | 0.0789 | 0.2290 | 1.0000 |
| 72 | Cb_CL_VN_B-Cb_CL_FPN_B | -0.0820 | -0.3150 | 0.1795 | 0.5161 | 0.8033 | -0.3734 | -0.5833 | -0.1275 | 0.0022 | 0.4225 |
| 73 | Cb_CL_VN_B-Cb_CL_DMN_A | 0.0534 | -0.1658 | 0.3079 | 0.6727 | 0.8773 | -0.2209 | -0.4662 | 0.0331 | 0.0769 | 1.0000 |
| 74 | Cb_CL_VN_B-Cb_CL_DMN_B | 0.1584 | -0.0827 | 0.3984 | 0.2076 | 0.7707 | -0.0825 | -0.3622 | 0.2127 | 0.5134 | 1.0000 |
| 75 | Cb_CL_SMN_A-Cb_CL_FPN_B | 0.2561 | 0.0196 | 0.4782 | 0.0395 | 0.3808 | 0.0294 | -0.2384 | 0.2950 | 0.8162 | 0.9907 |
| 76 | Cb_CL_SMN_A-Cb_CL_DMN_A | 0.2110 | -0.0601 | 0.4328 | 0.0916 | 0.5355 | 0.0628 | -0.2040 | 0.3327 | 0.6194 | 1.0000 |
| 77 | Cb_CL_SMN_B-Cb_CL_FPN_B | 0.0922 | -0.1888 | 0.3461 | 0.4650 | 0.7872 | -0.0976 | -0.3371 | 0.1458 | 0.4391 | 1.0000 |
| 78 | Cb_CL_SMN_B-Cb_CL_DMN_A | 0.2581 | -0.0079 | 0.4842 | 0.0379 | 0.3852 | -0.0530 | -0.2995 | 0.1688 | 0.6748 | 1.0000 |
| 79 | Cb_CL_SMN_B-Cb_CL_DMN_B | 0.0403 | -0.2331 | 0.2895 | 0.7498 | 0.8933 | -0.1046 | -0.3377 | 0.1535 | 0.4069 | 1.0000 |
| 80 | Cb_CL_DAN_A-Cb_CL_DMN_A | 0.0324 | -0.2199 | 0.2999 | 0.7979 | 0.9058 | -0.1032 | -0.3175 | 0.1169 | 0.4132 | 1.0000 |
| 81 | Cb_CL_DAN_A-Cb_CL_DMN_B | -0.0855 | -0.3420 | 0.1715 | 0.4982 | 0.8013 | -0.0013 | -0.2368 | 0.2340 | 0.9920 | 1.0000 |
| 82 | Cb_CL_DAN_B-Cb_CL_FPN_B | -0.0531 | -0.2875 | 0.1795 | 0.6745 | 0.8737 | -0.0692 | -0.2944 | 0.1871 | 0.5840 | 1.0000 |
| 83 | Cb_CL_DAN_B-Cb_CL_DMN_A | 0.1185 | -0.1164 | 0.3514 | 0.3470 | 0.7441 | 0.0149 | -0.2232 | 0.2426 | 0.9060 | 0.9935 |
| 84 | Cb_CL_DAN_B-Cb_CL_DMN_B | 0.0445 | -0.1943 | 0.3152 | 0.7251 | 0.9029 | -0.0595 | -0.2975 | 0.1807 | 0.6378 | 1.0000 |
| 85 | Cb_CL_VAN_A-Cb_CL_LN_A | 0.0731 | -0.1488 | 0.2956 | 0.5629 | 0.8169 | 0.1276 | -0.1260 | 0.3893 | 0.3109 | 1.0000 |
| 86 | Cb_CL_VAN_A-Cb_CL_FPN_A | 0.1318 | -0.1161 | 0.3677 | 0.2954 | 0.7404 | -0.1404 | -0.3766 | 0.1285 | 0.2648 | 1.0000 |
| 87 | Cb_CL_VAN_A-Cb_CL_FPN_B | 0.0760 | -0.1904 | 0.3465 | 0.5474 | 0.8127 | -0.0228 | -0.2647 | 0.2138 | 0.8569 | 0.9904 |
| 88 | Cb_CL_VAN_A-Cb_CL_TPN | -0.0422 | -0.2708 | 0.2220 | 0.7384 | 0.8963 | -0.0589 | -0.3205 | 0.1927 | 0.6411 | 1.0000 |
| 89 | Cb_CL_VAN_A-Cb_CL_DMN_A | 0.1678 | -0.0504 | 0.3897 | 0.1816 | 0.7788 | -0.0237 | -0.2428 | 0.1966 | 0.8516 | 0.9961 |
| 90 | Cb_CL_VAN_A-Cb_CL_DMN_B | 0.1070 | -0.1449 | 0.3748 | 0.3961 | 0.7568 | -0.1301 | -0.3526 | 0.1220 | 0.3017 | 1.0000 |
| 91 | Cb_CL_LN_A-Cb_CL_FPN_A | -0.0493 | -0.3332 | 0.2265 | 0.6964 | 0.8960 | -0.2130 | -0.4714 | 0.0585 | 0.0885 | 1.0000 |
| 92 | Cb_CL_LN_A-Cb_CL_FPN_B | -0.0291 | -0.2831 | 0.2119 | 0.8181 | 0.9233 | -0.1499 | -0.4104 | 0.1081 | 0.2334 | 1.0000 |
| 93 | Cb_CL_LN_A-Cb_CL_DMN_A | 0.1597 | -0.0762 | 0.4080 | 0.2038 | 0.7712 | -0.0219 | -0.2589 | 0.2244 | 0.8624 | 0.9908 |
| 94 | Cb_CL_LN_A-Cb_CL_DMN_B | -0.0260 | -0.2837 | 0.2262 | 0.8372 | 0.9286 | -0.1392 | -0.3634 | 0.1158 | 0.2688 | 1.0000 |
| 95 | Cb_IL_VN_B-Cb_CL_DAN_B | 0.1268 | -0.1421 | 0.3671 | 0.3142 | 0.7675 | 0.0520 | -0.1958 | 0.2859 | 0.6808 | 0.9955 |
| 96 | Cb_IL_SMN_A-Cb_CL_DMN_B | 0.1153 | -0.1236 | 0.3518 | 0.3606 | 0.7326 | -0.1489 | -0.3847 | 0.0990 | 0.2364 | 1.0000 |
| 97 | Cb_IL_SMN_B-Cb_CL_FPN_B | 0.1921 | -0.0713 | 0.4150 | 0.1253 | 0.6202 | -0.0441 | -0.2822 | 0.1758 | 0.7271 | 0.9953 |
| 98 | Cb_IL_SMN_B-Cb_CL_DMN_B | 0.1390 | -0.1064 | 0.3785 | 0.2695 | 0.7649 | -0.0961 | -0.3243 | 0.1532 | 0.4463 | 1.0000 |
| 99 | Cb_IL_DAN_B-Cb_CL_FPN_B | 0.1259 | -0.1096 | 0.3551 | 0.3176 | 0.7566 | -0.0003 | -0.2342 | 0.2456 | 0.9979 | 1.0000 |
| 100 | Cb_IL_DAN_B-Cb_CL_DMN_A | 0.0195 | -0.1994 | 0.2719 | 0.8772 | 0.9406 | -0.0042 | -0.2184 | 0.2107 | 0.9735 | 1.0000 |
| 101 | Cb_IL_DAN_B-Cb_CL_DMN_B | 0.0021 | -0.2361 | 0.2626 | 0.9866 | 1.0000 | -0.1117 | -0.3389 | 0.1075 | 0.3755 | 1.0000 |
| 102 | Cb_IL_VAN_A-Cb_CL_FPN_B | 0.2678 | 0.0384 | 0.4832 | 0.0310 | 0.3989 | 0.1489 | -0.0874 | 0.3588 | 0.2364 | 1.0000 |
| 103 | Cb_IL_VAN_A-Cb_CL_TPN | 0.0641 | -0.1700 | 0.2953 | 0.6120 | 0.8318 | -0.0924 | -0.3339 | 0.1380 | 0.4642 | 1.0000 |
| 104 | Cb_IL_VAN_A-Cb_CL_DMN_A | 0.2210 | -0.0218 | 0.4435 | 0.0769 | 0.4947 | 0.0370 | -0.2122 | 0.2753 | 0.7699 | 0.9906 |
| 105 | Cb_IL_VAN_A-Cb_CL_DMN_B | 0.2332 | -0.0253 | 0.4685 | 0.0616 | 0.4954 | 0.0779 | -0.1328 | 0.2917 | 0.5375 | 1.0000 |
| 106 | Cb_IL_VAN_B-Cb_CL_FPN_B | 0.1650 | -0.0593 | 0.3788 | 0.1891 | 0.7765 | 0.0214 | -0.2185 | 0.2699 | 0.8658 | 0.9829 |
| 107 | Cb_IL_VAN_B-Cb_CL_TPN | 0.1090 | -0.1553 | 0.3471 | 0.3873 | 0.7475 | -0.0324 | -0.2789 | 0.1988 | 0.7980 | 0.9937 |
| 108 | Cb_IL_VAN_B-Cb_CL_DMN_A | 0.1198 | -0.1072 | 0.3329 | 0.3420 | 0.7587 | -0.1103 | -0.3306 | 0.1637 | 0.3817 | 1.0000 |
| 109 | Cb_IL_VAN_B-Cb_CL_DMN_B | 0.1192 | -0.1260 | 0.3567 | 0.3441 | 0.7547 | -0.0811 | -0.3329 | 0.1594 | 0.5207 | 1.0000 |
| 110 | Cb_IL_LN_A-Cb_CL_DAN_B | -0.1718 | -0.4133 | 0.0973 | 0.1712 | 0.7509 | -0.0622 | -0.3028 | 0.2073 | 0.6227 | 1.0000 |
| 111 | Cb_IL_LN_A-Cb_CL_VAN_A | 0.1525 | -0.0829 | 0.3938 | 0.2254 | 0.7909 | 0.1354 | -0.1176 | 0.3651 | 0.2824 | 1.0000 |
| 112 | Cb_IL_LN_A-Cb_CL_FPN_A | -0.0162 | -0.2616 | 0.2415 | 0.8983 | 0.9473 | -0.0989 | -0.3067 | 0.1270 | 0.4332 | 1.0000 |
| 113 | Cb_IL_LN_A-Cb_CL_FPN_B | 0.0971 | -0.1604 | 0.3619 | 0.4416 | 0.7966 | 0.0131 | -0.2210 | 0.2201 | 0.9175 | 1.0000 |
| 114 | Cb_IL_LN_A-Cb_CL_DMN_A | 0.0870 | -0.1802 | 0.3410 | 0.4906 | 0.8093 | -0.0069 | -0.2647 | 0.2377 | 0.9565 | 1.0000 |
| 115 | Cb_IL_LN_A-Cb_CL_DMN_B | 0.1493 | -0.0908 | 0.3953 | 0.2352 | 0.7694 | -0.0797 | -0.3102 | 0.1737 | 0.5279 | 1.0000 |
| 116 | Cb_IL_FPN_A-Cb_CL_DMN_B | 0.0596 | -0.1963 | 0.3303 | 0.6375 | 0.8427 | 0.0858 | -0.1645 | 0.3178 | 0.4968 | 1.0000 |
| 117 | Cb_IL_FPN_B-Cb_CL_SMN_B | 0.2315 | -0.0164 | 0.4535 | 0.0635 | 0.4904 | 0.0499 | -0.1754 | 0.2771 | 0.6931 | 0.9909 |
| 118 | Cb_IL_FPN_B-Cb_CL_VAN_A | 0.2634 | 0.0389 | 0.4810 | 0.0340 | 0.3649 | 0.0358 | -0.2188 | 0.2616 | 0.7769 | 0.9864 |
| 119 | Cb_IL_TPN-Cb_CL_VN_B | 0.0616 | -0.1780 | 0.2996 | 0.6261 | 0.8392 | -0.0496 | -0.3200 | 0.2284 | 0.6945 | 0.9856 |
| 120 | Cb_IL_DMN_A-Cb_CL_VN_B | 0.0961 | -0.1565 | 0.3370 | 0.4465 | 0.7906 | -0.1597 | -0.3988 | 0.0928 | 0.2038 | 1.0000 |
| 121 | Cb_IL_DMN_A-Cb_CL_SMN_B | 0.1759 | -0.0835 | 0.3700 | 0.1609 | 0.7223 | 0.2634 | -0.0090 | 0.4818 | 0.0340 | 1.0000 |
| 122 | Cb_IL_DMN_A-Cb_CL_DAN_A | 0.1005 | -0.1567 | 0.3445 | 0.4259 | 0.7754 | 0.0833 | -0.1726 | 0.3276 | 0.5093 | 1.0000 |
| 123 | Cb_IL_DMN_A-Cb_CL_DAN_B | 0.0861 | -0.1667 | 0.3265 | 0.4950 | 0.8097 | 0.0261 | -0.2108 | 0.2584 | 0.8367 | 0.9968 |
| 124 | Cb_IL_DMN_A-Cb_CL_VAN_A | 0.1452 | -0.0872 | 0.3596 | 0.2486 | 0.7617 | 0.1813 | -0.0683 | 0.3947 | 0.1485 | 1.0000 |
| 125 | Cb_IL_DMN_A-Cb_CL_FPN_A | 0.2918 | 0.0469 | 0.4964 | 0.0183 | 0.3538 | 0.2237 | 0.0177 | 0.4301 | 0.0732 | 1.0000 |
| 126 | Ce_IL_SMN_B-Cb_C_VN_B | -0.1634 | -0.3888 | 0.0971 | 0.1933 | 0.7613 | 0.1147 | -0.1482 | 0.3479 | 0.3630 | 1.0000 |
| 127 | Ce_IL_SMN_B-Cb_C_SMN_B | -0.4140 | -0.5968 | -0.1987 | 0.0006 | 0.1173 | -0.0794 | -0.3295 | 0.1871 | 0.5298 | 1.0000 |
| 128 | Ce_IL_SMN_B-Cb_C_VAN_A | -0.2781 | -0.4875 | -0.0568 | 0.0249 | 0.4003 | -0.0977 | -0.3446 | 0.1520 | 0.4389 | 1.0000 |
| 129 | Ce_IL_DAN_B-Cb_C_DAN_B | -0.2288 | -0.4288 | 0.0060 | 0.0668 | 0.4603 | -0.1266 | -0.3472 | 0.1222 | 0.3149 | 1.0000 |
| 130 | Ce_IL_VAN_A-Cb_C_SMN_A | -0.3420 | -0.5368 | -0.1238 | 0.0053 | 0.2553 | -0.1014 | -0.3409 | 0.1418 | 0.4217 | 1.0000 |
| 131 | Ce_IL_VAN_A-Cb_C_SMN_B | -0.2749 | -0.4858 | -0.0233 | 0.0267 | 0.3959 | -0.0156 | -0.2549 | 0.1992 | 0.9018 | 1.0000 |
| 132 | Ce_IL_VAN_A-Cb_C_DAN_A | -0.1652 | -0.3831 | 0.0506 | 0.1884 | 0.7906 | 0.0389 | -0.2059 | 0.2843 | 0.7581 | 0.9954 |
| 133 | Ce_IL_VAN_A-Cb_C_DAN_B | -0.2280 | -0.4493 | 0.0027 | 0.0678 | 0.4511 | -0.0522 | -0.3091 | 0.1827 | 0.6798 | 1.0000 |
| 134 | Ce_IL_VAN_A-Cb_C_VAN_A | -0.3068 | -0.5075 | -0.0662 | 0.0129 | 0.3567 | -0.0521 | -0.2822 | 0.2015 | 0.6804 | 1.0000 |
| 135 | Ce_IL_LN_A-Cb_C_DAN_B | -0.1172 | -0.3408 | 0.1430 | 0.3527 | 0.7480 | -0.0880 | -0.3391 | 0.1740 | 0.4857 | 1.0000 |
| 136 | Ce_IL_LN_B-Cb_C_SMN_A | -0.2938 | -0.4937 | -0.0666 | 0.0175 | 0.3763 | -0.1033 | -0.3573 | 0.1398 | 0.4127 | 1.0000 |
| 137 | Ce_IL_FPN_A-Cb_C_FPN_A | -0.2702 | -0.4802 | -0.0299 | 0.0295 | 0.4070 | 0.0124 | -0.2301 | 0.2548 | 0.9219 | 0.9996 |
| 138 | Ce_IL_DMN_C-Cb_C_VAN_A | -0.0636 | -0.3111 | 0.1980 | 0.6146 | 0.8296 | 0.0420 | -0.2329 | 0.2982 | 0.7398 | 0.9985 |
| 139 | Ce_IL_VN_A-Cb_I_FPN_C | 0.0793 | -0.1813 | 0.3245 | 0.5300 | 0.8183 | 0.1835 | -0.0570 | 0.4092 | 0.1435 | 1.0000 |
| 140 | Ce_IL_VN_B-Cb_I_DAN_B | -0.0861 | -0.3067 | 0.1643 | 0.4954 | 0.8034 | -0.1221 | -0.3899 | 0.1383 | 0.3325 | 1.0000 |
| 141 | Ce_IL_SMN_B-Cb_I_VAN_A | -0.3848 | -0.5536 | -0.1765 | 0.0015 | 0.1495 | -0.0553 | -0.2946 | 0.1875 | 0.6615 | 1.0000 |
| 142 | Ce_IL_SMN_B-Cb_I_LN_A | -0.2841 | -0.5153 | -0.0284 | 0.0218 | 0.3830 | -0.1193 | -0.3561 | 0.1210 | 0.3439 | 1.0000 |
| 143 | Ce_IL_VAN_A-Cb_I_DAN_B | -0.2018 | -0.4399 | 0.0365 | 0.1069 | 0.5732 | -0.0492 | -0.2978 | 0.1967 | 0.6972 | 0.9822 |
| 144 | Ce_IL_VAN_A-Cb_I_VAN_A | -0.2297 | -0.4344 | 0.0122 | 0.0657 | 0.4696 | 0.0075 | -0.2503 | 0.2807 | 0.9527 | 1.0000 |
| 145 | Ce_IL_VAN_A-Cb_I_VAN_B | -0.1375 | -0.3401 | 0.0798 | 0.2748 | 0.7686 | 0.1002 | -0.1522 | 0.3546 | 0.4272 | 1.0000 |
| 146 | Ce_IL_VAN_A-Cb_I_LN_A | -0.3053 | -0.5190 | -0.0534 | 0.0134 | 0.3236 | -0.1694 | -0.3826 | 0.0903 | 0.1773 | 1.0000 |
| 147 | Ce_IL_VAN_B-Cb_I_VAN_B | -0.1370 | -0.3737 | 0.1220 | 0.2764 | 0.7514 | -0.0216 | -0.2635 | 0.2594 | 0.8646 | 0.9874 |
| 148 | Ce_IL_LN_A-Cb_I_DAN_B | 0.0072 | -0.2342 | 0.2286 | 0.9545 | 0.9905 | 0.0641 | -0.1921 | 0.3077 | 0.6119 | 1.0000 |
| 149 | Ce_IL_LN_B-Cb_I_DAN_B | -0.1319 | -0.3757 | 0.1221 | 0.2948 | 0.7487 | -0.1021 | -0.3473 | 0.1588 | 0.4182 | 1.0000 |
| 150 | Ce_IL_FPN_A-Cb_I_DAN_B | 0.0443 | -0.1778 | 0.2739 | 0.7259 | 0.8981 | 0.0969 | -0.1425 | 0.3565 | 0.4424 | 1.0000 |
| 151 | Ce_IL_FPN_A-Cb_I_FPN_A | -0.0708 | -0.3192 | 0.2073 | 0.5753 | 0.8104 | 0.1033 | -0.1230 | 0.3035 | 0.4130 | 1.0000 |
| 152 | Ce_IL_DMN_C-Cb_I_SMN_A | -0.0261 | -0.2891 | 0.2296 | 0.8365 | 0.9332 | 0.0620 | -0.1927 | 0.3272 | 0.6239 | 1.0000 |
| 153 | Ce_IL_DMN_C-Cb_I_DAN_B | -0.1128 | -0.3745 | 0.1314 | 0.3711 | 0.7384 | 0.0441 | -0.1891 | 0.2630 | 0.7274 | 0.9887 |
| 154 | Ce_IL_DMN_B-Cb_I_DAN_B | 0.1032 | -0.1508 | 0.3566 | 0.4133 | 0.7745 | -0.0808 | -0.3061 | 0.1562 | 0.5221 | 1.0000 |
| 155 | Ce_CL_VN_A-Cb_C_FPN_C | 0.1107 | -0.1518 | 0.3305 | 0.3799 | 0.7481 | 0.0563 | -0.1896 | 0.3135 | 0.6562 | 1.0000 |
| 156 | Ce_CL_LN_B-Cb_C_DAN_B | -0.1814 | -0.3849 | 0.0636 | 0.1482 | 0.6809 | -0.0456 | -0.2949 | 0.2242 | 0.7181 | 1.0000 |
| 157 | Ce_CL_FPN_C-Cb_C_FPN_A | 0.2661 | 0.0478 | 0.4756 | 0.0321 | 0.3875 | -0.0276 | -0.2950 | 0.2175 | 0.8275 | 0.9982 |
| 158 | Ce_CL_FPN_A-Cb_C_DAN_B | 0.1972 | -0.0487 | 0.3970 | 0.1154 | 0.5863 | -0.0606 | -0.3230 | 0.1939 | 0.6314 | 1.0000 |
| 159 | Ce_CL_DMN_C-Cb_C_FPN_A | 0.2110 | -0.0212 | 0.4624 | 0.0915 | 0.5521 | 0.0056 | -0.2893 | 0.2614 | 0.9650 | 1.0000 |
| 160 | Ce_CL_DMN_A-Cb_C_FPN_A | 0.1260 | -0.1381 | 0.3838 | 0.3171 | 0.7651 | 0.0840 | -0.1503 | 0.3257 | 0.5057 | 1.0000 |
| 161 | Ce_CL_DMN_A-Cb_C_DAN_B | 0.0193 | -0.2351 | 0.2624 | 0.8789 | 0.9371 | -0.1450 | -0.3851 | 0.0949 | 0.2491 | 1.0000 |
| 162 | Ce_CL_DMN_A-Cb_C_VAN_B | -0.0027 | -0.2671 | 0.2475 | 0.9828 | 1.0000 | 0.0673 | -0.1733 | 0.2988 | 0.5943 | 1.0000 |
| 163 | Ce_CL_DMN_B-Cb_C_FPN_A | 0.1289 | -0.1118 | 0.3555 | 0.3063 | 0.7580 | 0.0528 | -0.1997 | 0.2775 | 0.6761 | 1.0000 |
| 164 | Ce_CL_DMN_B-Cb_C_DAN_B | 0.0434 | -0.2096 | 0.2966 | 0.7315 | 0.8992 | -0.0851 | -0.3194 | 0.1455 | 0.5003 | 1.0000 |
| 165 | Ce_CL_DMN_B-Cb_C_VAN_A | 0.0840 | -0.1888 | 0.3430 | 0.5058 | 0.8068 | 0.0593 | -0.1997 | 0.2867 | 0.6390 | 1.0000 |
| 166 | Ce_CL_DMN_B-Cb_C_VAN_B | 0.0001 | -0.2519 | 0.2623 | 0.9994 | 0.9994 | -0.0189 | -0.2855 | 0.2290 | 0.8811 | 0.9944 |
| 167 | Ce_CL_VN_B-Cb_C_FPN_A | 0.1561 | -0.0772 | 0.3958 | 0.2143 | 0.7803 | -0.0987 | -0.3383 | 0.1562 | 0.4339 | 1.0000 |
| 168 | Ce_CL_VN_B-Cb_C_DAN_B | 0.0654 | -0.2002 | 0.3037 | 0.6047 | 0.8277 | -0.1317 | -0.3921 | 0.1459 | 0.2957 | 1.0000 |
| 169 | Ce_CL_SMN_A-Cb_C_FPN_B | 0.1345 | -0.1288 | 0.3862 | 0.2853 | 0.7648 | 0.0265 | -0.2107 | 0.2650 | 0.8338 | 0.9995 |
| 170 | Ce_CL_VAN_A-Cb_C_DAN_B | -0.0673 | -0.3249 | 0.1895 | 0.5943 | 0.8252 | -0.1108 | -0.3640 | 0.1521 | 0.3796 | 1.0000 |
| 171 | Ce_CL_VAN_B-Cb_C_DMN_A | 0.1008 | -0.1627 | 0.3392 | 0.4243 | 0.7798 | -0.0153 | -0.2673 | 0.2580 | 0.9036 | 0.9966 |
| 172 | Ce_CL_LN_A-Cb_C_DAN_B | -0.1464 | -0.3934 | 0.1019 | 0.2447 | 0.7742 | 0.0078 | -0.2322 | 0.2597 | 0.9507 | 1.0000 |
| 173 | Ce_CL_SMN_A-Cb_I_FPN_B | 0.0714 | -0.1648 | 0.3175 | 0.5718 | 0.8175 | 0.0550 | -0.1782 | 0.2857 | 0.6633 | 1.0000 |
| 174 | Ce_CL_SMN_A-Cb_I_DMN_B | 0.0434 | -0.1781 | 0.2701 | 0.7316 | 0.8937 | -0.0912 | -0.3202 | 0.1664 | 0.4699 | 1.0000 |
| 175 | Ce_CL_DAN_B-Cb_I_FPN_B | 0.1334 | -0.1236 | 0.3751 | 0.2895 | 0.7654 | 0.0867 | -0.1627 | 0.3129 | 0.4921 | 1.0000 |
| 176 | Ce_CL_DAN_B-Cb_I_DMN_A | 0.1463 | -0.0631 | 0.3753 | 0.2449 | 0.7624 | 0.0888 | -0.1505 | 0.3258 | 0.4817 | 1.0000 |
| 177 | Ce_CL_DAN_B-Cb_I_DMN_B | 0.1847 | -0.0545 | 0.4089 | 0.1407 | 0.6624 | -0.0569 | -0.2985 | 0.2042 | 0.6528 | 1.0000 |
| 178 | Ce_CL_VAN_A-Cb_I_SMN_B | -0.0759 | -0.2953 | 0.1691 | 0.5479 | 0.8072 | -0.0609 | -0.3184 | 0.1812 | 0.6297 | 1.0000 |
| 179 | Ce_CL_VAN_A-Cb_I_DAN_A | -0.0097 | -0.2648 | 0.2382 | 0.9389 | 0.9795 | -0.0092 | -0.2560 | 0.2255 | 0.9421 | 1.0000 |
| 180 | Ce_CL_VAN_A-Cb_I_VAN_A | -0.1013 | -0.3239 | 0.1264 | 0.4220 | 0.7832 | 0.0026 | -0.2449 | 0.2553 | 0.9837 | 1.0000 |
| 181 | Ce_CL_VAN_B-Cb_I_FPN_B | 0.0382 | -0.2345 | 0.2980 | 0.7627 | 0.9031 | 0.0695 | -0.1934 | 0.3192 | 0.5820 | 1.0000 |
| 182 | Ce_CL_VAN_B-Cb_I_DMN_B | 0.0477 | -0.2031 | 0.2950 | 0.7061 | 0.8850 | 0.1044 | -0.1434 | 0.3362 | 0.4077 | 1.0000 |
| 183 | Ce_CL_LN_A-Cb_I_DAN_B | -0.0790 | -0.3108 | 0.1674 | 0.5315 | 0.8141 | -0.0655 | -0.2798 | 0.1694 | 0.6041 | 1.0000 |
| 184 | Ce_CL_FPN_C-Cb_I_VAN_A | 0.0257 | -0.2486 | 0.2785 | 0.8392 | 0.9203 | 0.0372 | -0.2281 | 0.3141 | 0.7687 | 0.9957 |
| 185 | Ce_CL_FPN_C-Cb_I_FPN_A | 0.2652 | 0.0060 | 0.5031 | 0.0327 | 0.3716 | 0.1463 | -0.1312 | 0.4075 | 0.2448 | 1.0000 |
| 186 | Ce_CL_FPN_C-Cb_I_FPN_B | 0.1404 | -0.1164 | 0.3935 | 0.2646 | 0.7622 | 0.1266 | -0.1316 | 0.3763 | 0.3151 | 1.0000 |
| 187 | Ce_CL_FPN_C-Cb_I_DMN_B | 0.0766 | -0.1904 | 0.3350 | 0.5441 | 0.8140 | 0.1636 | -0.0967 | 0.4201 | 0.1927 | 1.0000 |
| 188 | Ce_CL_FPN_A-Cb_I_DAN_B | 0.0371 | -0.2230 | 0.2875 | 0.7692 | 0.9052 | -0.1005 | -0.3273 | 0.1781 | 0.4256 | 1.0000 |
| 189 | Ce_CL_DMN_A-Cb_I_VAN_A | 0.0282 | -0.2300 | 0.2877 | 0.8233 | 0.9239 | 0.1010 | -0.1805 | 0.3781 | 0.4233 | 1.0000 |
| 190 | Ce_CL_DMN_A-Cb_I_FPN_A | 0.3138 | 0.0638 | 0.5251 | 0.0109 | 0.4217 | 0.2669 | 0.0063 | 0.5009 | 0.0316 | 1.0000 |
| 191 | Ce_CL_DMN_A-Cb_I_TPN | 0.0258 | -0.2328 | 0.2609 | 0.8382 | 0.9244 | 0.0326 | -0.2476 | 0.2840 | 0.7963 | 0.9979 |
| 192 | Ce_CL_DMN_B-Cb_I_VN_B | 0.1373 | -0.1362 | 0.3829 | 0.2753 | 0.7591 | 0.1444 | -0.1216 | 0.3862 | 0.2511 | 1.0000 |
| 193 | Ce_CL_DMN_B-Cb_I_FPN_A | 0.2367 | -0.0152 | 0.4534 | 0.0576 | 0.4833 | 0.1767 | -0.0486 | 0.4029 | 0.1592 | 1.0000 |

NIHSS, National Institutes of Health Stroke Scale; *p*, uncorrected *p*-value; *p*_FDR, FDR-corrected *p*-value (q < 0.05); CI, confidence interval; Ce, cerebrum; Cb, cerebellum; IL, ipsilesional; CL, contralesional; I, ipsilateral; C, contralateral; VN, visual network; SMN, somatomotor network; DAN, dorsal attention network; VAN, ventral attention network; LN, limbic network; FPN, frontoparietal network; TPN, temporal parietal network; DMN, default mode network.
